# Mapping the obesity problems scale to the SF-6D: results based on the Scandinavian Obesity Surgery Registry (SOReg)

**DOI:** 10.1007/s10198-022-01473-7

**Published:** 2022-05-20

**Authors:** Sun Sun, Erik Stenberg, Yang Cao, Lars Lindholm, Klas-Göran Salén, Karl A. Franklin, Nan Luo

**Affiliations:** 1 Department of Epidemiology and Global Health, Umeå University, 90185 Umeå, Sweden; 2 Research Group Health Outcomes and Economic Evaluation, Department of Learning, Informatics, Management and Ethics, Karolinska Instiutet, Solna, Sweden; 3 Department of Surgery, Faculty of Medicine and Health, Örebro University, Örebro, Sweden; 4 Clinical Epidemiology and Biostatistics, School of Medical Sciences, Örebro University, Örebro, Sweden; 5 Department of Surgical and Perioperative Sciences, Surgery, Umeå University, Umeå, Sweden; 6 NUS Saw Swee Hock School of Public Health, National University of Singapore, Singapore, Singapore

**Keywords:** Mapping, Quality of life (QOL), Obesity-problem scale (OP), SF-6D, Obesity, Health utility, Cross-walk

## Abstract

**Background:**

*Obesity Problem Scale* (OP) is a widely applied instrument for obesity, however currently calculation of health utility based on *OP* is not feasible as it is not a *preference-based measure*. Using data from the *Scandinavian Obesity Surgery Registry (SOReg)*, we sought to develop a mapping algorithm to estimate *SF-6D utility* from *OP.* Furthermore, to test whether the mapping algorithm is robust to the effect of surgery.

**Method:**

The source data *SOReg* (*n* = 36 706) contains both *OP* and *SF-36,* collected at pre-surgery and at 1, 2 and 5 years post-surgery. The *Ordinary Least Square (OLS)*, *beta-regression* and *Tobit regression* were used to predict the SF-6D utility for different time points respectively. Besides the main effect model, different combinations of patient characteristics (age, sex, Body Mass Index, obesity-related comorbidities) were tested. Both internal validation (split-sample validation) and validation with testing the mapping algorithm on a dataset from other time points were carried out. A multi-stage model selection process was used, accessing model *consistency, parsimony*, *goodness-of-fit* and *predictive accuracy*. Models with the best performance were selected as the final mapping algorithms.

**Results:**

The final mapping algorithms were based on OP summary score using OLS models, for pre- and post-surgery respectively. Mapping algorithms with different combinations of patients’ characteristics were presented, to satisfy the user with a different need.

**Conclusion:**

This study makes available algorithms enabling crosswalk from the *Obesity Problem Scale* to the *SF-6D* utility. Different mapping algorithms are recommended for the mapping of pre- and post-operative data.

**Supplementary Information:**

The online version contains supplementary material available at 10.1007/s10198-022-01473-7.

## Introduction

Obesity is associated with significant mortality, reduced *quality of life* and increased risk of developing diseases such as diabetes mellitus, cardiovascular disorders and cancers [1–3]. Europe has the world’s second highest obesity rate (women 25%, men 22%) after North America (women 30%, men 24%) [4]. Management of obesity involves variety of treatment options: the first-line *lifestyle modification*, includes diet, physical activity, and behavioral therapy [5, 6]. This may be supplemented with adjunct *pharmacotherapy* [5, 7]. When these conventional treatments are partially efficacious in achieving sustained weight loss, *bariatric surgery* will be introduced [5, 7].

To make treatments comparisons, *cost-utility analysis* are required, particularly by reimbursement agencies and national advisory bodies such as the *National Institute for Health and Clinical Excellence (NICE)* in the UK [8], *the Dental and Pharmaceutical Benefits Agency* in Sweden [9] which put a request on health utility data to be collected in clinical studies. *Health utility* is often obtained through a *preference-based measure (PBM)* [10, 11]. The most commonly used PBM are EQ-5D [1], SF-6D [2], and Health Utilities Index (HUI) [3]. For obesity, the most commonly applied PBM is SF-6D [2].

However, not all clinical studies contain a PBM. Similarly, in obesity studies, quite often only *none-preference-based-measures (NPMB)* were used [12], such as *Obesity Problem Scale (OP)*, *Obesity and weight-loss Quality of life,* and *weight-related symptom measure (WRSM)*. The OP scale has been mostly applied in Scandinavia [13], but recently, has been recognized by the *American Society for Metabolic and Bariatric Surgery* [14]*.* When in the absence of PBM, it may be possible to map utility values indirectly from a NPBM as a solution [15].

Mapping is a relative new research area with most papers published after 2000 [16, 17]. For obesity, mapping algorithms have been estimated from *Moorehead-Ardelt II questionnaire (MA-II)* to SF-6D and EQ-5D [18], from *Weight on Quality of Life-Lite to SF-6D* [19]*.* However, to the best of our knowledge, currently there is no mapping algorithm for OP. In the *Scandinavian Region,* as both SF-36 and OP have been applied in the *Swedish Obesity Subjects* trial between 1987 and 2001 [20], as well as in the large national register for bariatric surgery in Sweden, the *Scandinavian Obesity Surgery Registry* since 2007 [20–22], which enables constructing a mapping algorithm from OP to SF-6D utility index. As the OP mainly measures the impact of obesity on psychosocial function [23], and the obesity level will be significantly reduced after bariatric surgery [24], we assume that the relationship between OP and SF-46 might be different for pre- and post-surgery.

The aim of the study is to provide a mapping algorithm to estimate SF-6D utility values from the *Obesity Problem Scale*, which can be used to estimate utilities in subsequent analyses, such as economic evaluations reliant on data sets that include only Obesity Problem Scale. Additionally, we explored different mapping models and to test whether the mapping algorithm was robust to the effect of bariatric surgery.

## Method

### Data source and study population

The *Scandinavian Obesity Surgery Registry (SOReg)* is a national research and quality registry for bariatric surgery in Sweden (> 97% national coverage), and is validated regularly and has been shown to have high data quality [21]. SOReg contains information on patient socio-demographic characteristics, provider characteristics, details regarding the procedure, and health outcomes including HRQoL assessed by SF-36 and OP. HRQoL data are reported by the patients at baseline and at 1, 2, and 5 years by filling in a questionnaire on paper. Nurses take anthropometric data and collect the applicable questionnaires. Trained personnel perform data inputting. For the current study, all subjects who received bariatric surgery from January 2011 to March 2019 with complete answers on OP scale and SF-6D were included (*n* = 36 706), with no other exclusion criteria being applied. The Ethics Authority in Sweden granted ethical permission for this study (reference number: 2019–03,666).

The development and validation of the mapping algorithm followed guidelines from ISPOR [25] and TRIPOD checklist [26] (Supplementary material Table S8). For internal cross-validation, data at each wave (baseline, 1–, 2– and 5-year follow-ups) were randomly split into two parts: 80% of the data were used as a training dataset for building models, and the remaining 20% were used as a validation dataset, thus resulting in totally eight datasets (training and validation datasets at each time point (baseline, 29,365 and 7342; 1-year, 27,125 and 5425; 2-year, 13,911 and 3478; 5-year, 5945 and 1488) (Supplementary material, Table S3. No significant differences in patient characteristics were found between the training and validation datasets. To know if the performance of the mapping algorithm differs between pre- and post-surgery, we also tested the mapping algorithm from one wave on datasets from all time points. For example, for baseline data, validations were carried out on baseline, 1-, 2-, and 5-year data, respectively. The mapping model with the best predictive performance was selected as the final model.

### Health outcomes measure

#### Short Form-36 (SF-36/RAND) and SF-6D

SF-36 measures HRQoL in eight domains (social functioning, physical function, role-physical, bodily-pain, general health, vitality, social functioning, role-emotional and mental health) [27, 28], and the SF-36-v1 has been applied in the SOReg. The *short form six-dimensions (SF-6D)* was developed to derive a preference-based score from the SF-36 [29] or its 12-item version (SF-12) [30], using a *standard gamble* method. The six SF-6D domains include pain, mental health, physical functioning, social functioning, role limitations, and vitality, and each is described into four to six functional levels. The SF-6D utility scores in the current study were calculated using the UK tariff [29] since there is a local tariff in Sweden. Details regarding SF-6D domains and relevant SF-36 items could be found in the supplemental material (S1 and S2).

#### Obesity problem scale

*Obesity problem scale (OP)* is a validated disease-specific instrument, which assesses the impact of obesity on psychosocial functioning [20, 23]. The instrument comprises eight items (private gatherings at home; private gatherings at a friend’s/relative’s home; going to restaurants; participation in community activities; holidays away from home; trying on and buying clothes; bathing in public places; intimate relations) on a four-point scale (significant difficulties; some difficulties; limited difficulties; no difficulties). Based on responses on the OP dimensions, an OP summary score can be calculated ranging from 0 to 100, with a higher score indicating more psychosocial dysfunction [20].

### Statistical methods for mapping

Descriptive analyses were used to examine the sample characteristics and the responses to the SF-6D and OP measures (proportions for discrete variables, mean and standard deviation, plus median and inter quart range for continuous variables).

We applied multivariate analysis to predict the values of the SF-6D utility score from OP summary score and items, with and without other covariates. Besides the commonly used Ordinary Least Square (OLS) method, both beta-regression (accounting for the fact that the SF-36 utility score is bounded between 0 and 1) [31] and Tobit regression (accounting for the fact that SF-6D index were centred at 0.301 and 1) [32] were used. In beta regression, to decide whether or not including a link function and which link function to use, we computed AIC and BIC for those with and without link functions, based on Model 1 (Supplementary materials Tables S9). Model with *Cauchit link function* performed the best, and was applied in all beta-regression analyses in the study. In order to make comparisons across OLS, Tobit and Beta regression, as well as for easily interpreting the results, both transformed OP summary score $\left( {\frac{{100 - OP_{{{\text{raw}}}} }}{{100}} \times \frac{{\left( {N - 1} \right) + 0.5}}{N},\left( {N = 500} \right)} \right)$ and transformed SF-6D index $\left( {SF - 6D_{{{\text{raw}}}} \frac{{\left( {N - 1} \right) + 0.5}}{N}\left( {N = 500} \right)} \right)$ were used, as beta-regression does not allow the value of the dependant variable and variable used in the link function to be 0 or 1 [33]. Both transformed SF-6D index and OP summary score ranged between 0 and 1, with a higher value indicating better health.

Five sets of modes were tested, OP as the main effect (Model 1); including age and sex (Model 2); including age, sex and BMI (model 3); including age, sex and comorbidities (Model 4); including age, sex, BMI and comorbidities (Model 5). All the models were run on the baseline, 1, 2, and 5 years follow-up datasets, respectively.

Two types of independent variables were constructed for the OP measure: Type A is a simple additive model, where the transformed OP summary score was used as the independent variable. In Type B modelling, the item responses for each OP dimensions were used as independent variables and three dummy variables (reference: “no difficulty”) were created for levels “not so bothered”, “mostly bothered” and “definitely bothered”, respectively. As there are eight OP dimensions, totally 24 dummy variables were included in the models.

### Model selection

Model goodness-of-fit was assessed using adjusted/pseudo R^2^ statistics in ordinary least squares (OLS)/Beta regression, *Bayesian information criteria (BIC),* and *Alkaike information criteria (AIC)* statistics. Lower BIC and AIC values would indicate a better fitting model. To examine the predictive performance of the model, the differences between the predicted and observed SF-6D value at the individual level were used to compute the *mean absolute error (MAE)* and *root-mean-square error (RMSE)*. Smaller error values were indicative of better-performing models. All analyses were conducted using R.4.0.2 [34].

## Results

### Patient characteristics

Patient’s characteristics were reported in Table 1. More than 76% of the patients were female, and the mean age was 41 at the baseline. About 10% of the patients were current smokers. Mean BMI was 42 at the baseline, and decreased to 29 at follow-ups. In general, the presence of obesity-related comorbidities (sleep apnoea, hypertension, diabetes, dyslipidaemia and depression) has decreased overtime, lowest at 1-year follow-up, followed by 2- and 5-year follow-ups.

**Table 1:** Socio-demographic characteristics, at baseline, 1, 2 and 5 years follow-ups

	Baseline (*n* = 36,706)		1 year (*n* = 27,125)		2 year (*n* = 17,389)		5 year (*n* = 7437)	
	*n*	%	*n*	%	*n*	%	*n*	%
*Age*								
18–35 yrs	11,994	32.7	7448	27.5	3966	22.8	1179	15.9
36–45 yrs	11,168	30.4	8066	29.7	4876	28.0	1810	24.3
46–55 yrs	9655	26.3	7877	29.0	5405	31.1	2426	32.6
56–65 yrs	3662	10.0	3468	12.8	2817	16.2	1664	22.4
65 +	227	0.6	266	1.0	325	1.9	358	4.8
Mean (SD)	41.02 (11.2)		42.73 (11.18)	44.59 (11.22)	47.92 (11.17)			
Median (Q1, Q2)	41 (32, 49)		43 (34, 51)		45 (37, 53)		48 (40, 56)	
*Sex*								
Men	8637	23.5	6439	23.7	4032	23.2	1662	22.3
Women	28,069	76.5	20,686	76.3	13,357	76.8	5775	77.7
*Educational level*								
Missing	22,450	61.2	15,909	58.7	8920	51.3	3802	51.1
Less than 9 school years	7627	20.8	5999	22.1	4565	26.3	1954	26.3
9–12 school years	1588	4.3	1203	4.4	944	5.4	414	5.6
More 12 school years	5041	13.7	4014	14.8	2960	17.0	1267	17.0
*Smoking*								
Yes	3746	10.2	2669	9.8	1592	9.2	755	10.2
No	22,670	61.8	16,812	62.0	10,293	59.2	4469	60.1
Don't know	3992	10.9	2824	10.4	2270	13.1	910	12.2
Occationally	5626	15.3	4414	16.3	3013	17.3	1272	17.1
Quit before operation	670	1.8	406	1.5	221	1.3	31	0.4
*BMI*								
Mean(SD)	41.57(5.63)		28.55(4.56)		28.41(4.65)		29.86(4.93)	
Median(Q1, Q2)	40.8(37.7, 44.6)	27.9(25.3, 31.1)	27.8(25.1, 30.9)	29.3(26.3, 32.6)				
*Sleep aponea*								
Missing	2	0.0	206	0.8	333	1.9	222	3.0
No	32,913	89.7	26,046	96.0	16,556	95.2	7039	94.6
Yes	3791	10.3	873	3.2	500	2.9	176	2.4
*Hypertension*								
No	27,554	75.1	22,321	82.3	13,911	80.0	5643	75.9
Yes	9150	24.9	4598	17.0	3145	18.1	1572	21.1
Diabetes								
Missing	2	0.0	206	0.8	333	1.9	222	3.0
No	32,003	87.2	25,709	94.8	16,154	92.9	6744	90.7
Yes	4701	12.8	1210	4.5	902	5.2	471	6.3
*Dyslipidemia*								
Missing	2	0.0	206	0.8	333	1.9	222	3.0
No	33,213	90.5	25,440	93.8	16,018	92.1	6719	90.3
Yes	3491	9.5	1479	5.5	1038	6.0	496	6.7
*Depression*								
Missing	2	0.0	206	0.8	333	1.9	222	3.0
No	30,707	83.7	23,257	85.7	14,521	83.5	5959	80.1
Yes	5997	16.3	3662	13.5	2535	14.6	1256	16.9
*Year*								
2011	5625	15.3	4721	17.4	3582	20.6	2991	40.2
2012	5426	14.8	4439	16.4	3047	17.5	2189	29.4
2013	5399	14.7	4373	16.1	3199	18.4	1628	21.9
2014	4886	13.3	3865	14.2	2738	15.7	578	7.8
2015	4462	12.2	3734	13.8	2165	12.5	19	0.3
2016	4142	11.3	2697	9.9	1871	10.8	17	0.2
2017	3225	8.8	2245	8.3	778	4.5	7	0.1
2018	2522	6.9	1046	3.9	6	0.0	7	0.1
2019	1019	2.8	5	0.0	3	0.0	1	0.0

### Patient-reported health outcomes

Details regarding reporting on OP and SF-6D were reported in Table 2. HRQoL improved after surgery, with the highest improvements observed at 1-year follow-up, followed by 2- and 5-year follow-ups. The SF-6D index was close to normal distribution at the baseline but left-skewed at the follow-ups (Supplementary material Figure S1 and S2). There were moderate (0.4–0.59) and high correlations (≥ 0.6) between the SF-6D index and OP summary score at all time wave, as well as with most of the OP dimensions (Supplementary material Table S4).

**Table 2:** Reporting of Obesity problem scale (dimension, summary score) and SF-6D index score, at baseline, 1, 2 and 5 years follow-ups

	Baseline (*n* = 36,706)		1 year (*n* = 27,125)		2 year (*n* = 17,389)		5 year (*n* = 7437)	
	*n*	*%*	*n*	*%*	*n*	*%*	*n*	*%*
*Obesity problem scale dimensions*								
*Private gatherings in my own home (OP1)*								
No difficulties	7723	*21.0*	20,841	*76.8*	12,840	*73.8*	5029	*67.6*
Limited difficulties	7846	*21.4*	3978	*14.7*	2633	*15.1*	1266	*17.0*
Some difficulties	13,276	*36.2*	1736	*6.4*	1429	*8.2*	809	*10.9*
Significant difficulties	7861	*21.4*	570	*2.1*	487	*2.8*	333	*4.5*
*Private gatherings in a friend’s or relative’s home (OP2)*								
No difficulties	4924	*13.4*	19,773	*72.9*	12,158	*69.9*	4675	*62.9*
Limited difficulties	5020	*13.7*	4303	*15.9*	2703	*15.5*	1308	*17.6*
Some difficulties	13,036	*35.5*	2344	*8.6*	1872	*10.8*	1036	*13.9*
Significant difficulties	13,726	*37.4*	705	*2.6*	656	*3.8*	418	*5.6*
*Going to a restaurant (OP3*								
No difficulties	6563	*17.9*	19,362	*71.4*	12,337	*70.9*	5055	*68.0*
Limited difficulties	7811	*21.3*	4427	*16.3*	2753	*15.8*	1229	*16.5*
Some difficulties	14,045	*38.3*	2703	*10.0*	1776	*10.2*	888	*11.9*
Significant difficulties	8287	*22.6*	633	*2.3*	523	*3.0*	265	*3.6*
*Going to community activities, courses etc. (OP4)*								
No difficulties	6647	*18.1*	20,869	*76.9*	12,887	*74.1*	5086	*68.4*
Limited difficulties	7759	*21.1*	3964	*14.6*	2645	*15.2*	1253	*16.8*
Some difficulties	13,596	*37.0*	1714	*6.3*	1367	*7.9*	778	*10.5*
Significant difficulties	8704	*23.7*	578	*2.1*	490	*2.8*	320	*4.3*
*Vacations away from home (OP5)*								
No difficulties	6344	*17.3*	20,615	*76.0*	12,656	*72.8*	5084	*68.4*
Limited difficulties	6378	*17.4*	3614	*13.3*	2412	*13.9*	1120	*15.1*
Some difficulties	13,446	*36.6*	2121	*7.8*	1625	*9.3*	862	*11.6*
Significant difficulties	10,538	*28.7*	775	*2.9*	696	*4.0*	371	*5.0*
*Trying on and buying clothes (OP6)*								
No difficulties	1555	*4.2*	18,101	*66.7*	10,729	*61.7*	3947	*53.1*
Limited difficulties	1810	*4.9*	4506	*16.6*	2886	*16.6*	1335	*18.0*
Some difficulties	7317	*19.9*	3349	*12.3*	2564	*14.7*	1380	*18.6*
Significant difficulties	26,024	*70.9*	1169	*4.3*	1210	*7.0*	775	*10.4*
*Bathing in public places (beach, public pool, etc.) (OP7)*								
No difficulties	2367	*6.4*	12,576	*46.4*	7909	*45.5*	3219	*43.3*
Limited difficulties	2413	*6.6*	4485	*16.5*	2539	*14.6*	1198	*16.1*
Some difficulties	7585	*20.7*	5861	*21.6*	3697	*21.3*	1575	*21.2*
Significant difficulties	24,341	*66.3*	4203	*15.5*	3244	*18.7*	1445	*19.4*
*Intimate relations (OP8)*								
No difficulties	4909	*13.4*	15,013	*55.3*	9189	*52.8*	3795	*51.0*
Limited difficulties	5606	*15.3*	4637	*17.1*	2778	*16.0*	1278	*17.2*
Some difficulties	12,711	*34.6*	4711	*17.4*	3176	*18.3*	1373	*18.5*
Significant difficulties	13,480	*36.7*	2764	*10.2*	2246	*12.9*	991	*13.3*
*OP summary score*								
Mean (SD)	65.11 (26.06)		18 (21.86)		20.37 (24.32)	23.61 (26.74)		
Median (Q1, Q2)	70.8 (50, 83.3)		8.3 (0, 29.2)		12.5 (0, 33.3)		12.5 (0, 41.7)	
*Transformed OP summary score*								
Mean (SD)	0.35 (0.26)		0.82 (0.22)		0.8 (0.24)		0.76 (0.27)	0.35 (0.26)
Median (Q1, Q2)	0.29 (0.17, 0.5)		0.92 (0.71, 1)		0.87 (0.67, 1)		0.87 (0.58, 1)	0.29 (0.17, 0.5)
Mean (SD)	0.66 (0.13)		0.80 (0.14)		0.78 (0.15)		0.75 (0.15)	
Median (Q1, Q2)	0.65 (0.57, 0.75)		0.84 (0.71, 0.89)		0.82 (0.67, 0.89)		0.79 (0.63, 0.89)	
*Transformed SF-6D index*								
Mean (SD)	0.66 (0.13)		0.80 (0.14)		0.78 (0.15)		0.75 (0.15)	
Median (Q1, Q2)	0.65 (0.57, 0.75)		0.84 (0.71, 0.89)		0.82 (0.67, 0.89)		0.79 (0.63, 0.89)	

### Initial model development

Results for model goodness of fit and prediction accuracy for the initial model development at each time point (baseline, 1, 2, 5-year follow-up) are reported in Table 3, details can be found in Supplementary materials, for OLS (Table S5A and S5B), for Tobit regression (S6A-S6B) and beta regression (S7A and S7B). Four main issues were investigated: whether using OP summary score or item as a predictor? Whether including other covariates such as age, sex, BMI and comorbidity? Whether using a separate mapping algorithm for pre- and post-surgery? Whether using OLS, Tobit or beta regression?

**Table 3:**
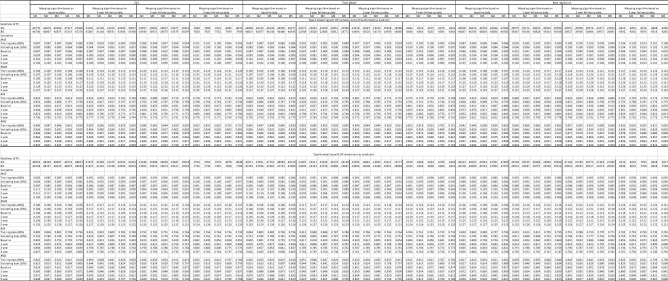
Comparison of model goodness of fit and prediction accuracy, transformed SF-6D index^a^ and OP Summary Score ^b^ used

^a^$${\text{SF}} - 6D_{\text{transformed}} = {\text{SF}} - 6D_{\text{raw}} \times \frac{{\left( {N - 1} \right) + 0.5}}{N} \left( {N = 500} \right)$$

^b^$${\text{OPS}}_{{{\text{transformed}}}} = \frac{{100 - {\text{OPS}}_{{{\text{raw}}}} }}{{100}} \times \frac{{\left( {N - 1} \right) + 0.5}}{N},~\left( {N = 500} \right)$$

#### OP summary score or item as predictor (Type A or B model)

Across the OLS, Tobit and beta regression, the application of OP dimensions instead of the OP summary score did not increase the model performance, as it had little impact on goodness-of-fit and prediction power. Furthermore, for OLS models, inconsistency was found for the dimension *bathing in public places (beach, public pool, OP7),* with positive coefficients at baseline.

#### Inclusion of age, sex BMI and comorbidity as predictors

Conclusions from beta regression and Tobit regression were similar to OLS models, that the inclusion of age, sex, BMI and comorbidity variables increased model performance: in terms of goodness-of-fit, an increased R^2^ and decreased AIC and BIC across Mode 1 to 5 for each wave of data; in terms of prediction power, decreased MAE and RMSE for model validations were also observed across Model 1 to 5.

#### OLS, Tobit or beta regression

Results for the goodness of fit and prediction power are presented in Table 3 In terms of goodness of fit, OLS yielded lowest AIC and BIC values for mapping algorithm from baseline and 2-year follow-up, while Beta regression gave the lowest AIC and BIC values for mapping algorithm from 1- and 5-year follow-ups. In terms of prediction power, results were similar for OLS and Tobit models, both yielded lower MAE and RAE values and higher RMSE and RRSE values relative to beta regressions at almost all time points; The performance of OLS and Tobit models were rather similar, both yielded better results than beta-regression.

#### Comparison between Pre- and post-surgery algorithms

Coefficients for pre- and post-surgery algorithms showed different patterns: coefficients for the OP summary score differed between baseline (0.26) and follow-ups (0.32). Coefficients for age groups were rather stable across all the models. The coefficient for male was higher than the coefficient for female at follow-ups, but not at baseline. At baseline, coefficients for BMI were significant; however, at follow-ups, not all coefficients for BMI were significant. Coefficients for comorbidities were relatively stable from baseline to 2-year follow-up, with depression associated with the largest effect, followed by sleep apnoea and diabetes. Hypertension and dyslipidaemia had a very low impact. At the 5-year follow-up, only depression was significant.

### Final mapping algorithm

Based on the above findings, we conclude that using OP summary score as the main predictor, including age, sex, BMI and comorbidities, using OLS model, and separate analyses for pre- and post- surgery. For comorbidities, sleep apnoea, diabetes and depression were included as those were with significant coefficients and also confirmed by the clinicians as the most important obesity-related comorbidities. We include BMI into the algorithm for pre-surgery prediction, but exclude BMI for post-surgery prediction as it led to inconsistency (higher BMI was not associated with lower SF-6D index). We ran Model 1 to 5 for baseline data, and Model 1, 2 and 4 for post-surgery data (Table 4). As beta regression was not used for deriving the final mapping algorithms, it was not necessary to use the transformed SF-6D and OP summary score. Therefore, we ran OLS model with the raw OP summary score (ranged 0–100, with a higher value indicating worse health) as the predictor and raw SF-6D index (ranged 0–1, with a higher value indicating better health). We recommend Model 5 for mapping with pre-surgery data, and Model 4 for post-surgery data. When not all information of predictors are available, one may choose any algorithm from Model 1–4 for pre-surgery data, and Model 1 or 2 for post-surgery based on their own need or preferences.

**Table 4:** Mapping algorithm based on OLS model, untransformed SF-6D index and OP summary score used, baseline and post-surgery data, respectively

	Baseline										Post-surgery (1,2,5 years)									
	Model 1		Model 2		Model 3		Model 4		Model 5		Model 1		Model 2		Model 3		Model 4		Model 5	
	Coefficient	*p*-Value	Coefficient	*p*-Value	Coefficient	*p*-Value	Coefficient	*p*-Value	Coefficient	*p*-Value	Coefficient	*p*-Value	Coefficient	*p*-Value	Coefficient	*p*-Value	Coefficient	*p*-Value	Coefficient	*p*-Value
Intercept	0.831	0.000	0.841	0.000	0.847	0.000	0.842	0.000	0.848	0.000	0.852	0.000	0.866	0.000	0.867	0.000	0.872	0.000	0.872	0.000
OP summary score	– 0.003	0.000	– 0.003	0.000	– 0.003	0.000	– 0.003	0.000	– 0.003	0.000	– 0.003	0.000	– 0.003	0.000	– 0.003	0.000	– 0.003	0.000	– 0.003	0.000
*Patient characteristics*																				
*Age*^*a*^																				
36–45 yrs	–	–	0.001	0.547	0.000	0.818	0.004	0.015	0.003	0.105	–	–	– 0.013	0.000	– 0.013	0.000	– 0.009	0.000	– 0.009	0.000
46–55 yrs	–	–	– 0.009	0.000	– 0.011	0.000	– 0.004	0.028	– 0.006	0.001	–	–	– 0.022	0.000	– 0.022	0.000	– 0.017	0.000	– 0.017	0.000
56–65 yrs	–	–	– 0.017	0.000	– 0.019	0.000	– 0.010	0.000	– 0.012	0.000	–	–	– 0.033	0.000	– 0.033	0.000	– 0.028	0.000	– 0.028	0.000
65 +	–	–	– 0.018	0.033	– 0.020	0.015	– 0.010	0.232	-0.013	0.135	–	–	– 0.034	0.000	– 0.034	0.000	– 0.030	0.000	– 0.029	0.000
*Man*^*b*^	–	–	– 0.007	0.000	– 0.005	0.001	– 0.006	0.000	-0.004	0.011	–	–	0.010	0.000	0.011	0.000	0.007	0.000	0.007	0.000
*BMI*^*c*^																				
40–44	–	–	–	–	-0.007	0.000	-	-	– 0.007	0.000	–	–	–	–	– 0.020	0.000	-	–	– 0.015	0.001
45–49	–	–	–	–	-0.013	0.000	-	-	– 0.012	0.000	–	–	–	–	– 0.023	0.018	-	–	– 0.015	0.111
50 +	–	–	–	–	-0.022	0.000	-	-	– 0.020	0.000	–	–	–	–	– 0.014	0.416	-	–	– 0.017	0.318
*Comobidity*^*d*^																				
Sleep apnea	–	–	–	–	–	–	– 0.015	0.000	– 0.014	0.000	–	–	–	–	–	–	– 0.019	0.000	– 0.018	0.000
Diabetes	–	–	–	–	–	–	– 0.011	0.000	– 0.011	0.000	–	–	–	–	–	–	– 0.013	0.000	– 0.012	0.000
Depression	–	–	–	–	–	–	– 0.033	0.000	– 0.033	0.000	–	–	–	–	–	–	– 0.078	0.000	– 0.078	0.000
*Goodness-of fit*																				
Adj-Rsq	0.280		0.282		0.285		0.293		0.295		0.290		0.296		0.296		0.333		0.332	
AIC	– 46,770		– 46,874		– 46,966		– 47,327		– 47,409		– 57,683		– 58,060		– 57,218		– 59,492		– 59,387	
BIC	– 46,746		– 46,807		– 46,875		– 47,236		– 47,293		– 57,657		– 57,991		– 57,123		– 59,397		– 59,267	
*Validation*																				
*MAE*																				
Training dataset	0.087		0.087		0.087		0.086		0.086		0.096		0.096		0.095		0.092		0.092	0.087
Cross-validation	0.085		0.085		0.085		0.084		0.084		0.097		0.097		0.096		0.094		0.094	0.085
Baseline	0.087		0.087		0.087		0.086		0.086		0.088		0.088		0.090		0.088		0.089	0.087
1-year	0.099		0.098		0.097		0.096		0.095		0.095		0.094		0.094		0.091		0.091	0.099
2-year	0.101		0.101		0.100		0.098		0.097		0.097		0.097		0.097		0.094		0.094	0.101
5-year	0.103		0.103		0.102		0.100		0.100		0.100		0.099		0.099		0.095		0.095	0.103
*RMSE*																				
Training dataset	0.109		0.109		0.109		0.108		0.108		0.121		0.121		0.120		0.117		0.117	0.109
Cross-validation	0.107		0.107		0.106		0.106		0.106		0.122		0.121		0.121		0.118		0.118	0.107
Baseline	0.109		0.109		0.108		0.108		0.108		0.112		0.112		0.114		0.111		0.113	0.109
1-year	0.120		0.120		0.119		0.118		0.117		0.119		0.118		0.118		0.115		0.115	0.120
2-year	0.124		0.123		0.123		0.121		0.121		0.123		0.122		0.122		0.119		0.119	0.124
5-year	0.127		0.127		0.127		0.124		0.125		0.127		0.126		0.125		0.122		0.122	0.127
*RAE*																				
Training dataset	0.823		0.821		0.819		0.814		0.813		0.799		0.795		0.796		0.771		0.771	0.823
Cross-validation	0.810		0.806		0.806		0.797		0.797		0.815		0.812		0.812		0.790		0.790	0.810
Baseline	0.820		0.818		0.817		0.811		0.810		0.833		0.832		0.841		0.832		0.838	0.820
1-year	0.844		0.840		0.838		0.825		0.822		0.821		0.818		0.818		0.796		0.796	0.844
2-year	0.822		0.820		0.819		0.802		0.801		0.798		0.796		0.796		0.770		0.770	0.822
5-year	0.796		0.793		0.793		0.776		0.775		0.771		0.767		0.767		0.740		0.739	0.796
*RRSE*																				
Training dataset	0.849		0.847		0.846		0.841		0.839		0.843		0.839		0.839		0.817		0.817	0.849
Cross-validation	0.836		0.833		0.832		0.826		0.825		0.851		0.848		0.848		0.828		0.828	0.836
Baseline	0.846		0.844		0.843		0.838		0.836		0.861		0.861		0.865		0.860		0.861	0.846
1-year	0.866		0.864		0.862		0.849		0.848		0.858		0.855		0.855		0.835		0.835	0.866
2-year	0.849		0.849		0.848		0.832		0.832		0.843		0.841		0.841		0.817		0.817	0.849
5-year	0.832		0.831		0.832		0.815		0.815		0.827		0.822		0.822		0.798		0.797	0.832

Reference group

^a^18–34 years

^b^Women

^c^BMI <  =  39

^d^No disease

## Discussion

This study explored mapping algorithms from OP to SF-6D index using a large patient register. Conceptual overlap between the source measure and the target PBM should be considered before mapping can be undertaken [35]. The OP has been developed as a condition-specific instrument to measure the impact of obesity on psychosocial function [23]. Although the focus of OP was on mental health and role function mental, dimensions such as *Vacations away from home, Trying on and buying clothes, Bathing in public places (beach, public pool, *etc.*), and Intimate relations* would also indicate the impact of obesity on physical health and pain. Therefore, we considered that there was a reasonable overlapping between OP and SF-6D, which was also indicating by the *R*^2^ in the mapping algorithm (0.3).

One important finding of our study was that the mapping algorithm should be different for data collected before and after bariatric surgery, which is in line with a recent study [36]. We found that the effect of the OP summary score increased while the effect of gender decreased after surgery and that the effect of BMI disappeared after the surgery. Possible explanation could be that pre-surgery patients were associated with very high BMI, and the there were remained effects of BMI on SF-6D utility even after controlling for OP; However, patients who underwent bariatric surgery lost weight significantly [24], and those with higher pre-operative BMI tend to lose a higher percentage of their total weight [37], thus all the effects of BMI were picked up by OP already. This finding suggests that mapping algorithm might differ at baseline and follow-ups for bariatric surgery, and one should be cautious to merge pre-operative and post-operative data to construct mapping algorithms, or to use follow-up data to examine the prediction power of mapping algorithm based on baseline data, or vice versa. To the best of our knowledge, this is the first evidence showing that clinical interventions may affect the crosswalk between an NPBM and a PBM among patients received bariatric surgery. Further research using data from other disease/intervention populations is needed to assess its generalizability.

We have chosen a simple additive model (with the OP summary score as the main predictor) for constructing the final mapping algorithm. This model assumed that the dimensions of the OP were equally important, and all levels carried equal weight; and response choices to each item lie on a similar interval scale. The models including all individual OP dimensions have a large number of independent variables; however, in terms of prediction ability, those did not outperform the simple additive models. Moreover, some of the coefficients were non-significant or non-monotonic. These findings were in line with previous studies using item response models or adding interaction and other terms [16]. Furthermore, in most published clinical studies, only the OP summary scores were reported. Therefore, we recommend using the simple additive model to map OP data to the SF-6D index scores.

The distributional characteristics of the SF-6D health utility data (UK v1 tariff) posed a challenge for modelling analysis, for example, the values being bounded between 0.301 and 1, skewness, multimodality, and gaps in the values [16, 17, 38]. In our study, we have tested OLS, Tobit, and beta-regression. The performance of OLS and Tobit was quite similar, both were superior to beta-regression. One possible explanation might be that SF-6D index does not suffer from the ceiling effect as much as the EQ-5D index, and in our study, the mean and median of SF-6D were rather close at baseline. In a study which was focused on the application of beta-regression on SF-6D index, the author claimed that the confidence intervals were overlapping across OLS and beta-regressions, suggesting that no model was superior to the others [33]. Although OLS has been criticized for not being appropriate for none-normally distributed data and might underestimate health utility associated with mild health states and overestimated utility for more severe health states [25, 38], there was no obvious evidence that OLS performed worse than other more complicated statistical models. The easy understanding and application of OLS made it a popular choice for deriving mapping algorithm. The *ISPOR guideline for mapping* does not advocate any specific statistical methods, with the reasons being *“… the performance of different methods will vary according to the characteristics of the target utility measure, the disease and patient population in question, the nature of the explanatory clinical variables, and the form of intended use in the CEA*[15]*.*” Like many investigators of mapping studies, we would recommend using the OLS model in this study.

Age and sex were commonly included as a predictor in mapping algorithms, and clinical outcomes such as BMI were also frequently included [16]. In the current study, we observed that in terms of goodness-of-fit and prediction power, mapping algorithms containing more predictors performed better than those with the fewer predictor. However, to satisfy the user with a different need, we presented algorithms with different combinations of predictors.

For estimating mapping algorithms, clinical trials were the most common source of data [17]. However, it is debatable whether it is optimal to use trial data for deriving mapping algorithms. Comparing with registry data, trial data are often derived from smaller, more homogeneous patients samples, thus limiting the generalizability of the resultant mapping algorithms to the real world [17].

Although many mapping studies applied split-sample validation, it is questioned that this approach might reduce the sample size used in the mapping estimation and might have no proven benefit [25]. However, it is quite often the case that there is no external dataset available for external validation. Furthermore, unlike the majority of the mapping studies using data from clinical trials, our study is based on a clinical registry with a rather large sample size, we still consider it appropriate to apply split-sample validation.

The main strength of our study was the use of real-world data from a large national patient register and the provision of multiple mapping algorithms using different combinations of predictors. The main limitation of the study was that some surgical centres had a low response rate HRQoL. Since most centres in Sweden have similar characteristics in patient cohorts, this is unlikely to have a significant impact on the representativeness of our study sample. Moreover, lost to follow-up at 5 year was higher relative to 1-, and 2- year, which might explain the insignificant results in some of the analyses. The implication of missing data needs to be investigated in future studies [39].

## Conclusion

This study makes available algorithms enabling crosswalk from the Obesity Problem Scale to the SF-6D for cost-utility analyses of interventions in obesity treatment. Different mapping algorithms are recommended for the mapping of pre-operative and post-operative data.

## Supplementary Information

Below is the link to the electronic supplementary material.

Supplementary file1 (PDF 640 KB)

## Data Availability

Data sharing is not possible according to Swedish law.

## References

[1] Kolotkin RL, Crosby RD, Williams GR, Hartley GG, Nicol S (2001). The relationship between health-related quality of life and weight loss. Obes. Res..

[2] Lindekilde N, Gladstone BP, Lübeck M, Nielsen J, Clausen L, Vach W, Jones A (2015). The impact of bariatric surgery on quality of life: a systematic review and meta-analysis. Obes. Rev..

[3] Megías Á, González-Cutre D, Beltrán-Carrillo VJ, Gomis-Díaz JM, Cervelló E, Bartholomew KJ (2018). The impact of living with morbid obesity on psychological need frustration: A study with bariatric patients. Stress Health.

[4] Branca, F., Nikogosian, H., & Lobstein, T. (Eds.). (n.d.).: The challenge of obesity in the WHO European Region and the strategies for response: summary.

[5] Jensen MD, Ryan DH, Donato KA, Apovian CM, Ard JD, Comuzzie AG (2014). Executive summary: Guidelines (2013) for the management of overweight and obesity in adults. Obesity.

[6] National Institute for Health and Care Excellence (NICE). (n.d.). Obesity: guidance on the prevention, identification, assessment and management of overweight and obesity in adults and children (NICE clinical guideline No. CG43). Retrieved from https://www.nice.org.uk/guidance/cg43

[7] American Association of Clinical Endocrinologists, The Obesity Society, and American Society for Metabolic & Bariatric Surgery. Clinical practice guidelines for the perioperative nutritional, Metabolic, and nonsurgical support of the bariatric surgery patient -- 2013 update. Surg. Obes. Relat. Dis. 9(2): 159 (2013). 10.1016/j.soard.2012.12.010 23537696

[8] National Institute of Health and Clinical Excellence (NICE). (2008). Guide to the Methods of Technology Appraisal. London: National Institute of Health and Clinical Excellence. Retrieved from https://www.nice.org.uk/process/pmg9/resources/guide-to-the-methods-of-technology-appraisal-2013-pdf-2007975843781 27905712

[9] TLV. (n.d.). The Dental and Pharmaceutical Benefits Agency(Tandvårds- och läkemedelsförmånsverket TLV). text. Retrieved May 6, 2021, from https://www.tlv.se/in-english.html

[10] Spiker B, Revicki D, Spiker B (1996). Taxonomy of quality of life. Quality of life and pharmacoeconomics in clinical trials.

[11] Fayers PM, Machin D (2006). Quality of life: the assessment, analysis and interpretation of patient-reported outcomes.

[12] Hachem A, Brennan L (2016). Quality of life outcomes of bariatric surgery: a systematic review. Obes. Surg..

[13] Hedenbro JL, Näslund E, Boman L, Lundegårdh G, Bylund A, Ekelund M, Näslund I (2015). Formation of the Scandinavian Obesity Surgery Registry. SOReg. Obes. Surg..

[14] Greene, M., Goldman, R., Chang, D., Hutter, M.: A5180 - The development of patient reported outcomes for national implementation in the MBSAQIP lessons learned from the PCORI funded LOBSTER PROMs Alpha Pilot. Surg. Obes. Relat. Dis. (2017). 13(10, Supplement), S147–S148. 10.1016/j.soard.2017.09.324

[15] Hermandez M, Marc B, Busschbach JJV (2017). Mapping to estimate health-state utility from non-preference-based outcome measures. Value Health.

[16] Brazier JE, Yang Y, Tsuchiya A, Rowen DL (2010). A review of studies mapping (or cross walking) non-preference based measures of health to generic preference-based measures. Eur. J. Health Econ..

[17] Mukuria C, Rowen D, Harnan S, Rawdin A, Wong R, Ara R, Brazier J (2019). An updated systematic review of studies mapping (or cross-walking) measures of health-related quality of life to generic preference-based measures to generate utility values. Appl. Health Econ. Health Policy.

[18] Stefan S, Sylvia W, Karin D, Luigi A, Masdevall NC, Manuel G-C, Marc I (2009). Mapping utility scores from a disease-specific quality-of-life measure in bariatric surgery patients. Value Health.

[19] Brazier JE, Kolotkin RL, Crosby RD, Williams GR (2004). Estimating a preference-based single Index for the Impact of Weight on Quality of Life-Lite (IWQOL-Lite) Instrument from the SF-6D. Value Health.

[20] Karlsson J, Sjöström L, Sullivan M (1998). Swedish obese subjects (SOS): an intervention study of obesity. Two-year follow-up of health-related quality of life (HRQL) and eating behavior after gastric surgery for severe obesity. Int. J. Obes. Relat. Metabol. Disord..

[21] Sundbom M, Näslund I, Näslund E, Ottosson J (2021). High acquisition rate and internal validity in the Scandinavian Obesity Surgery Registry. Surg. Obes. Relat. Dis..

[22] Karlsson J, Taft C, Rydén A, Sjöström L, Sullivan M (2007). Ten-year trends in health-related quality of life after surgical and conventional treatment for severe obesity: the SOS intervention study. Int. J. Obes..

[23] Karlsson J, Taft C, Sjöström L, Torgerson JS, Sullivan M (2003). Psychosocial functioning in the obese before and after weight reduction: construct validity and responsiveness of the Obesity-related Problems scale. Int. J. Obes..

[24] Garb J, Welch G, Zagarins S, Kuhn J, Romanelli J (2009). Bariatric surgery for the treatment of morbid obesity: a meta-analysis of weight loss outcomes for laparoscopic adjustable gastric banding and laparoscopic gastric bypass. Obes. Surg..

[25] Wailoo AJ, Hernandez-Alava M, Manca A, Mejia A, Ray J, Crawford B, Busschbach J (2017). Mapping to estimate health-state utility from non–preference-based outcome measures: An ISPOR good practices for outcomes research task force report. Value Health.

[26] Transparent reporting of a multivariable prediction model for individual prognosis or diagnosis (TRIPOD): The TRIPOD statement | The EQUATOR Network. (n.d.). Retrieved January 13, 2022, from https://www.equator-network.org/reporting-guidelines/tripod-statement/10.1186/s12916-014-0241-z PMC4284921 25563062

[27] Ware JE, Gandek B (1998). Overview of the SF-36 health survey and the international quality of life assessment (IQOLA) project. J. Clin. Epidemiol..

[28] Monica, 1776 Main Street Santa, & California 90401–3208. (n.d.). 36-Item Short Form Survey from the RAND Medical Outcomes Study. Retrieved March 29, 2018, from https://www.rand.org/health/surveys_tools/mos/36-item-short-form.html

[29] Brazier J, Roberts J, Deverill M (2002). The estimation of a preference-based measure of health from the SF-36. J. Health Econ..

[30] Brazier JE, Roberts J (2004). The estimation of a preference-based measure of health from the SF-12. Med. Care.

[31] Cribari-Neto, F., Zeileis, A.: Beta regression in R. J. Statist. Software. (2010). 10.18637/jss.v034.i02

[32] Kleiber, C., & Zeileis, A. (2020). *AER: Applied Econometrics with R*. Retrieved from https://CRAN.R-project.org/package=AER

[33] Hunger M, Baumert J, Holle R (2011). Analysis of SF-6D index data: is beta regression appropriate?. Value Health.

[34] R Core Team. (2019). R: A language and environment for statistical computing. Vienna, Austria: R Foundation for Statistical Computing. Retrieved from https://www.R-project.org/

[35] Round J, Hawton A (2017). Statistical alchemy: conceptual validity and mapping to generate health state utility values. PharmacoEconom. Open.

[36] Hernández Alava M, Wailoo A, Pudney S, Gray L, Manca A (2020). Mapping clinical outcomes to generic preference-based outcome measures: development and comparison of methods. Health Technol. Assess..

[37] Stenberg E, Näslund I, Persson C, Szabo E, Sundbom M, Ottosson J, Näslund E (2020). The association between socioeconomic factors and weight loss 5 years after gastric bypass surgery. Int. J. Obes..

[38] Wailoo A (2015). Modeling health state utility values in ankylosing spondylitis: comparisons of direct and indirect methods. Value in Health.

[39] Kedestig J, Stenberg E (2019). Loss to follow-up after laparoscopic gastric bypass surgery - a post hoc analysis of a randomized clinical trial. Surg. Obes. Relat. Dis..

