# Supplementary material for: Mapping the obesity problems scale to the SF-6D: results based on the Scandinavian Obesity Surgery Registry (SOReg)
Source: Eur J Health Econ. 2022 May 20;24(2):279–92. doi: 10.1007/s10198-022-01473-7 (PMC9985564; doi:10.1007/s10198-022-01473-7)
Supplement: Supplementary file 1 — Supplementary file1 (PDF 640 KB) [file 10198_2022_1473_MOESM1_ESM.pdf]

# Supplementary Material

Mapping the Obesity Problems Scale to the SF-6D: results based on the Scandinavian Obesity Surgery Registry (SOReg)

**S1. The short form-6D (SF-6D)[a]**

| | |
|---|---|
| **Physical functioning** | |
| 1 | Your health does not limit you in vigorous activities |
| 2 | Your health limits you a little in vigorous activities |
| 3 | Your health limits you a little in moderate activities |
| 4 | Your health limits you a lot in moderate activities |
| 5 | Your health limits you a little in bathing and dressing |
| 6 | Your health limits you a lot in bathing and dressing |
| **Role limitations** | |
| 1 | You have no problems with your work or other regular daily activities as a result of your physical health or any emotional problems |
| 2 | You are limited in the kind of work or other activities as a result of your physical health |
| 3 | You accomplish less than you would like as a result of emotional problems |
| 4 | You are limited in the kind of work or other activities as a result of your physical health and accomplish less than you would like as a result of emotional problems |
| **Social functioning** | |
| 1 | Your health limits your social activities none of the time |
| 2 | Your health limits your social activities a little of the time |
| 3 | Your health limits your social activities some of the time |
| 4 | Your health limits your social activities most of the time |
| 5 | Your health limits your social activities all of the time |
| **Pain** | |
| 1 | You have no pain |
| 2 | You have pain but it does not interfere with your normal work (both outside the home and housework) |
| 3 | You have pain that interferes with your normal work (both outside the home and housework) a little bit |
| 4 | You have pain that interferes with your normal work (both outside the home and housework) moderately |
| 5 | You have pain that interferes with your normal work (both outside the home and housework) quite a bit |
| 6 | You have pain that interferes with your normal work (both outside the home and housework) extremely |
| **Mental health** | |
| 1 | You feel tense or downhearted and low none of the time |
| 2 | You feel tense or downhearted and low a little of the time |
| 3 | You feel tense or downhearted and low some of the time |
| 4 | You feel tense or downhearted and low most of the time |
| 5 | You feel tense or downhearted and low all of the time |
| **Vitality** | |
| 1 | You have a lot of energy all of the time |
| 2 | You have a lot of energy most of the time |
| 3 | You have a lot of energy some of the time |
| 4 | You have a lot of energy a little of the time |
| 5 | You have a lot of energy none of the time |

a The SF-36 items used to construct the SF-6D are as follows: physical functioning items 1, 2 and 10; role limitation due to physical problems item 3; role limitation due to emotional problems item 2; social functioning item 2; both bodily pain items; mental health items 1 (alternate version) and 4; and vitality item 2.

S2. SF-36 items used to construct the SF-6D

| SF-6D domain | SF-36 item | |
|---|---|---|
| **Physical function** | **pf1** | The following items are about activities you might do during a typical day. Does your health now limit you in these activities? If so, how much?<br>Vigorous activities, such as running, lifting heavy objects, participating in strenuous sports. |
| | **pf2** | The following items are about activities you might do during a typical day. Does your health now limit you in these activities? If so, how much?<br>Moderate activities, such as moving a table, pushing a vacuum cleaner, bowling, or playing golf |
| | **pf10** | The following items are about activities you might do during a typical day. Does your health now limit you in these activities? If so, how much?<br>Bathing or dressing yourself |
| **Role participation** | **rp3** | During the past 4 weeks, have you had any of the following problems with your work or other regular daily activities as a result of your physical health?<br>Were limited in the kind of work or other activities |
| | **re2** | During the past 4 weeks, have you had any of the following problems with your work or other regular daily activities as a result of any emotional problems (such as feeling depressed or anxious)?<br>Accomplished less than you would like |
| **Social function** | **sf2** | During the past 4 weeks, how much of the time has your physical health or emotional problems interfered with your social activities (like visiting with friends, relatives, etc.) |
| **Bodily pain** | **bp1** | How much bodily pain have you had during the past 4 weeks? |
| | **bp2** | During the past 4 weeks, how much did pain interfere with your normal work (including both work outside the home and housework)? |
| **Mental health** | **mh1** | These questions are about how you feel and how things have been with you during the past 4 weeks. For each question, please give the one answer that comes closest to the way you have been feeling. How much of the time during the past 4 weeks…Have you been a very nervous person? |
| | **mh4** | These questions are about how you feel and how things have been with you during the past 4 weeks. For each question, please give the one answer that comes closest to the way you have been feeling. How much of the time during the past 4 weeks…Have you felt downhearted and blue? |
| **Vitality** | **vt2** | These questions are about how you feel and how things have been with you during the past 4 weeks. For each question, please give the one answer that comes closest to the way you have been feeling. How much of the time during the past 4 weeks…Did you have a lot of energy? |

Table S3. Socio-demographic characteristics, at baseline, 1, 2 and 5 years follow-ups, for training and validating datasets, respectively

| | Baseline data | | | | 1-year follow-up data | | | | 2-year follow-up data | | | | 5-year follow-up data | | | |
|---|---|---|---|---|---|---|---|---|---|---|---|---|---|---|---|---|
| | Training dataset | | Validating dataset | | Training dataset | | Validating dataset | | Training dataset | | Validating dataset | | Training dataset | | Validating dataset | |
| | n | % | n | % | n | % | n | % | n | % | n | % | n | % | n | % |
| **Age** | | | | | | | | | | | | | | | | |
| 18-35 yrs | 9592 | 32.7 | 2402 | 32.7 | 5896 | 27.2 | 1552 | 28.6 | 3186 | 22.9 | 780 | 22.4 | 940 | 15.8 | 239 | 16.1 |
| 36-45 yrs | 8979 | 30.6 | 2190 | 29.8 | 6451 | 29.7 | 1615 | 29.8 | 3942 | 28.3 | 934 | 26.9 | 1458 | 24.5 | 352 | 23.7 |
| 46-55 yrs | 7702 | 26.2 | 1953 | 26.6 | 6344 | 29.2 | 1533 | 28.3 | 4309 | 31 | 1096 | 31.5 | 1911 | 32.1 | 515 | 34.6 |
| 56-65 yrs | 2922 | 10 | 740 | 10.1 | 2789 | 12.9 | 679 | 12.5 | 2214 | 15.9 | 603 | 17.3 | 1349 | 22.7 | 315 | 21.2 |
| 65+ | 170 | 0.6 | 57 | 0.8 | 220 | 1 | 46 | 0.8 | 260 | 1.9 | 65 | 1.9 | 291 | 4.9 | 67 | 4.5 |
| *Mean(SD)* | 40.99(0.13) | | 41.15(0.14) | | 42.82(0.15) | | 42.37(0.15) | | 44.53(0.13) | | 44.85(0.14) | | 47.95(0.15) | | 47.81(0.15) | |
| *Median(Q1, Q2)* | 41(32, 49) | | 42(32, 50) | | 43(35, 51) | | 43(34, 51) | | 45(36, 53) | | 46(37, 53) | | 48(40, 56) | | 48(40, 56) | |
| **Sex** | | | | | | | | | | | | | | | | |
| Men | 22427 | 76.4 | 5643 | 76.9 | 16540 | 76.2 | 4146 | 76.4 | 10685 | 76.8 | 2672 | 76.8 | 4599 | 77.3 | 1176 | 79 |
| Women | 6938 | 23.6 | 1699 | 23.1 | 5160 | 23.8 | 1279 | 23.6 | 3226 | 23.2 | 806 | 23.2 | 1350 | 22.7 | 312 | 21 |
| **BMI** | | | | | | | | | | | | | | | | |
| *Mean(SD)* | 41.57(0.13) | | 41.56(0.14) | | 28.56(0.15) | | 28.54(0.15) | | 28.39(0.13) | | 28.5(0.14) | | 29.86(0.15) | | 29.85(0.15) | |
| *Median(Q1, Q2)* | 40.8(37.7, 44.6) | | 40.8(37.6, 44.6) | | 27.9(25.3, 31.1) | | 28(25.3, 31) | | 27.7(25.1, 30.9) | | 28(25.3, 31.1) | | 29.3(26.3, 32.7) | | 29.3(26.5, 32.4) | |
| **Comorbidities** | | | | | | | | | | | | | | | | |
| Sleep apnea | 3010 | 10.3 | 781 | 10.6 | 713 | 3.3 | 160 | 3 | 398 | 2.9 | 102 | 3 | 149 | 2.6 | 27 | 1.9 |
| Hypertension | 7310 | 24.9 | 1841 | 25.1 | 3704 | 17.2 | 894 | 16.6 | 2515 | 18.4 | 630 | 18.5 | 1271 | 22 | 301 | 20.8 |
| Diabetes | 3765 | 12.8 | 937 | 12.8 | 971 | 4.5 | 239 | 4.4 | 726 | 5.3 | 176 | 5.2 | 378 | 6.6 | 93 | 6.4 |
| Dyslipidemia | 2774 | 9.4 | 717 | 9.8 | 1195 | 5.5 | 284 | 5.3 | 830 | 6.1 | 208 | 6.1 | 411 | 7.1 | 85 | 5.9 |
| Dyspepsia | 3163 | 10.8 | 785 | 10.7 | 1628 | 7.6 | 377 | 7 | 1148 | 8.4 | 312 | 9.2 | 539 | 9.3 | 131 | 9.1 |
| Diarrhea | 473 | 1.6 | 114 | 1.6 | 265 | 1.2 | 73 | 1.4 | 225 | 1.6 | 71 | 2.1 | 175 | 3 | 32 | 2.2 |
| Depression | 4817 | 16.4 | 1181 | 16.1 | 2962 | 13.8 | 700 | 13 | 2034 | 14.9 | 501 | 14.7 | 1003 | 17.4 | 253 | 17.5 |
| **OP summary score** | | | | | | | | | | | | | | | | |
| *Mean(SD)* | 65.06(0.13) | | 65.28(0.14) | | 18.1(0.15) | | 17.6(0.15) | | 20.36(0.13) | | 20.41(0.14) | | 23.54(0.15) | | 23.89(0.15) | |
| *Median(Q1, Q2)* | 70.8(50, 83.3) | | 70.8(50, 87.5) | | 8.3(0, 29.2) | | 8.3(0, 29.2) | | 12.5(0, 33.3) | | 12.5(0, 33.3) | | 12.5(0, 37.5) | | 12.5(0, 41.7) | |
| **SF-6D index** | | | | | | | | | | | | | | | | |
| *Mean(SD)* | 0.66(0.13) | | 0.66(0.14) | | 0.80(0.15) | | 0.80(0.15) | | 0.78(0.13) | | 0.78(0.14) | | 0.75(0.15) | | 0.76(0.15) | |
| *Median(Q1, Q2)* | 0.7(0.6, 0.8) | | 0.6(0.6, 0.8) | | 0.8(0.7, 0.9) | | 0.8(0.7, 0.9) | | 0.8(0.7, 0.9) | | 0.8(0.7, 0.9) | | 0.8(0.6, 0.9) | | 0.8(0.6, 0.9) | |

Table S4. Correlation between Obesity Proble scale and SF-6D index

| | Baseline data | 1-year follow-up data |
|---|---|---|
| **OP summary score** [a] | -0.533 | -0.515 |
| **OP dimensions** [b] | | |
| *Private gatherings in my own home (OP1)* | -0.492 | -0.408 |
| *Private gatherings in a friend's or relative's home (OP2)* | -0.468 | -0.428 |
| *Going to a restaurant (OP3)* | -0.429 | -0.358 |
| *Going to community activities, courses etc (OP4)* | -0.487 | -0.427 |
| *Vacations away from home (OP5)* | -0.434 | -0.405 |
| *Trying on and buying clothes (OP6)* | -0.312 | -0.387 |
| *Bathing in public places (beach, public pool, etc) (OP7)* | -0.316 | -0.335 |
| *Intimate relations (OP8)* | -0.400 | -0.367 |

Moderate correlation (0.4-0.59)

Highe correlation (>=0.6)

a Pearson correlation coefficient

b Spearman correlation coefficient

| 2-year follow-up data | 5-year follow-up data | Total |
|---|---|---|
| **SF-6D index** | | |
| -0.538 | -0.568 | -0.637 |
| | | |
| -0.449 | -0.480 | -0.587 |
| -0.470 | -0.499 | -0.599 |
| -0.401 | -0.448 | -0.548 |
| -0.464 | -0.497 | -0.600 |
| -0.441 | -0.473 | -0.574 |
| -0.422 | -0.464 | -0.555 |
| -0.371 | -0.412 | -0.498 |
| -0.403 | -0.451 | -0.514 |

## Table S5A. Mapping algorithm based on OLS models, Type A,  transfored SF-6D index applied

**Mapping algorithm based on baseline data**

| | Model 1 | | Model 2 | | Model 3 | | Model 4 | | Model 5 | |
|---|---|---|---|---|---|---|---|---|---|---|
| | coefficient | p-Value | coefficient | p-Value | coefficient | p-Value | coefficient | p-Value | coefficient | p-Value |
| **Intercept** | 0.570 | 0.000 | 0.573 | 0.000 | 0.580 | 0.000 | 0.580 | 0.000 | 0.587 | 0.000 |
| **OP summary score (transformed)** | 0.261 | 0.000 | 0.268 | 0.000 | 0.268 | 0.000 | 0.261 | 0.000 | 0.261 | 0.000 |
| **Log scale, Tobit model** | - | - | - | - | - | - | - | - | - | - |
| **phi (beta regression)** | | | | | | | | | | |
| *phi_Intercept* | - | - | - | - | - | - | - | - | - | - |
| *phi_OPS_transform* | - | - | - | - | - | - | - | - | - | - |
| *Patient characteristics* | - | - | - | - | - | - | - | - | - | - |
| *Age* [a] | | | | | | | | | | |
| 36-45 yrs | - | - | 0.001 | 0.547 | 0.000 | 0.818 | 0.004 | 0.009 | 0.003 | 0.084 |
| 46-55 yrs | - | - | -0.009 | 0.000 | -0.011 | 0.000 | -0.003 | 0.103 | -0.005 | 0.005 |
| 56-65 yrs | - | - | -0.017 | 0.000 | -0.019 | 0.000 | -0.008 | 0.002 | -0.010 | 0.000 |
| 65+ | - | - | -0.018 | 0.033 | -0.020 | 0.015 | -0.008 | 0.329 | -0.011 | 0.192 |
| Man [b] | - | - | -0.007 | 0.000 | -0.005 | 0.001 | -0.005 | 0.001 | -0.004 | 0.019 |
| *BMI* [c] | | | | | | | | | | |
| 40-44 | - | - | - | - | -0.007 | 0.000 | - | - | -0.007 | 0.000 |
| 45-49 | - | - | - | - | -0.013 | 0.000 | - | - | -0.012 | 0.000 |
| 50+ | - | - | - | - | -0.022 | 0.000 | - | - | -0.020 | 0.000 |
| *Comobidity* [d] | | | | | | | | | | |
| Sleep apnea | - | - | - | - | - | - | -0.015 | 0.000 | -0.014 | 0.000 |
| Hypertension | - | - | - | - | - | - | -0.002 | 0.278 | -0.001 | 0.554 |
| Diabetes | - | - | - | - | - | - | -0.009 | 0.000 | -0.010 | 0.000 |
| Dyslipidemia | - | - | - | - | - | - | -0.003 | 0.197 | -0.004 | 0.105 |
| Depression | - | - | - | - | - | - | -0.033 | 0.000 | -0.033 | 0.000 |
| **Goodness of fit** | | | | | | | | | | |
| Adj-Rsq, or pseudo R2 | 0.282 | | 0.285 | | 0.293 | | 0.295 | | 0.263 | |
| AIC | -46770 | | -46874 | | -46966 | | -47327 | | -47408 | |
| BIC | -46746 | | -46807 | | -46875 | | -47219 | | -47276 | |
| **Validation** | | | | | | | | | | |
| *MAE* | | | | | | | | | | |
| Training data (80%) | 0.087 | | 0.087 | | 0.087 | | 0.086 | | 0.086 | |
| Validating data (20%) | 0.085 | | 0.085 | | 0.085 | | 0.084 | | 0.084 | |
| Baseline | 0.087 | | 0.087 | | 0.087 | | 0.086 | | 0.086 | |
| 1-year | 0.099 | | 0.098 | | 0.097 | | 0.096 | | 0.095 | |
| 2-year | 0.101 | | 0.101 | | 0.100 | | 0.098 | | 0.097 | |
| 5-year | 0.103 | | 0.103 | | 0.102 | | 0.100 | | 0.100 | |
| RMSE | | | | | | | | | | |
| Training data (80%) | 0.109 | | 0.109 | | 0.109 | | 0.108 | | 0.108 | |
| Validating data (20%) | 0.107 | | 0.107 | | 0.106 | | 0.106 | | 0.106 | |
| Baseline | 0.109 | | 0.109 | | 0.108 | | 0.108 | | 0.108 | |
| 1-year | 0.120 | | 0.120 | | 0.119 | | 0.118 | | 0.117 | |
| 2-year | 0.124 | | 0.123 | | 0.123 | | 0.121 | | 0.121 | |
| 5-year | 0.127 | | 0.127 | | 0.127 | | 0.124 | | 0.125 | |
| RAE | | | | | | | | | | |
| Training data (80%) | 0.823 | | 0.821 | | 0.819 | | 0.814 | | 0.813 | |
| Validating data (20%) | 0.810 | | 0.806 | | 0.806 | | 0.797 | | 0.796 | |
| Baseline | 0.820 | | 0.818 | | 0.817 | | 0.810 | | 0.810 | |
| 1-year | 0.844 | | 0.840 | | 0.838 | | 0.824 | | 0.822 | |
| 2-year | 0.822 | | 0.820 | | 0.819 | | 0.802 | | 0.801 | |
| 5-year | 0.796 | | 0.793 | | 0.793 | | 0.776 | | 0.775 | |
| RRSE | | | | | | | | | | |
| Training data (80%) | 0.849 | | 0.847 | | 0.846 | | 0.841 | | 0.839 | |
| Validating data (20%) | 0.836 | | 0.833 | | 0.832 | | 0.826 | | 0.825 | |
| Baseline | 0.846 | | 0.844 | | 0.843 | | 0.838 | | 0.836 | |
| 1-year | 0.866 | | 0.864 | | 0.862 | | 0.849 | | 0.847 | |
| 2-year | 0.849 | | 0.849 | | 0.848 | | 0.832 | | 0.832 | |
| 5-year | 0.832 | | 0.831 | | 0.832 | | 0.815 | | 0.815 | |

**Mapping algorithm based on 1-year follow-up data**

| | Model 1 | | Model 2 | | Model 3 | | Model 4 | | Model 5 | |
|---|---|---|---|---|---|---|---|---|---|---|
| | coefficient | p-Value | coefficient | p-Value | coefficient | p-Value | coefficient | p-Value | coefficient | p-Value |
| **Intercept** | 0.536 | 0.000 | 0.543 | 0.000 | 0.544 | 0.000 | 0.567 | 0.000 | 0.568 | 0.000 |
| **OP summary score (transformed)** | 0.322 | 0.000 | 0.325 | 0.000 | 0.325 | 0.000 | 0.308 | 0.000 | 0.307 | 0.000 |
| **Log scale, Tobit model** | - | - | - | - | - | - | - | - | - | - |
| **phi (beta regression)** | | | | | | | | | | |
| *phi_Intercept* | - | - | - | - | - | - | - | - | - | - |
| *phi_OPS_transform* | - | - | - | - | - | - | - | - | - | - |
| *Patient characteristics* | - | - | - | - | - | - | - | - | - | - |
| *Age* [a] | | | | | | | | | | |
| 36-45 yrs | - | - | -0.010 | 0.000 | -0.010 | 0.000 | -0.006 | 0.006 | -0.006 | 0.005 |
| 46-55 yrs | - | - | -0.018 | 0.000 | -0.018 | 0.000 | -0.012 | 0.000 | -0.012 | 0.000 |
| 56-65 yrs | - | - | -0.028 | 0.000 | -0.028 | 0.000 | -0.019 | 0.000 | -0.019 | 0.000 |
| 65+ | - | - | -0.020 | 0.015 | -0.019 | 0.018 | -0.011 | 0.190 | -0.011 | 0.178 |
| Man [b] | - | - | 0.010 | 0.000 | 0.011 | 0.000 | 0.008 | 0.000 | 0.008 | 0.000 |
| *BMI* [c] | | | | | | | | | | |
| 40-44 | - | - | - | - | -0.015 | 0.017 | - | - | -0.009 | 0.163 |
| 45-49 | - | - | - | - | -0.033 | 0.030 | - | - | -0.023 | 0.124 |
| 50+ | - | - | - | - | 0.009 | 0.699 | - | - | 0.011 | 0.633 |
| *Comobidity* [d] | | | | | | | | | | |
| Sleep apnea | - | - | - | - | - | - | -0.019 | 0.000 | -0.019 | 0.000 |
| Hypertension | - | - | - | - | - | - | -0.009 | 0.000 | -0.009 | 0.000 |
| Diabetes | - | - | - | - | - | - | -0.012 | 0.004 | -0.012 | 0.004 |
| Dyslipidemia | - | - | - | - | - | - | -0.001 | 0.829 | -0.001 | 0.829 |
| Depression | - | - | - | - | - | - | -0.072 | 0.000 | -0.072 | 0.000 |
| **Goodness of fit** | | | | | | | | | | |
| Adj-Rsq, or pseudo R2 | 0.268 | | 0.269 | | 0.303 | | 0.302 | | 0.285 | |
| AIC | -31091 | | -31228 | | -31019 | | -32049 | | -32027 | |
| BIC | -31067 | | -31164 | | -30931 | | -31945 | | -31900 | |
| **Validation** | | | | | | | | | | |
| *MAE* | | | | | | | | | | |
| Training data (80%) | 0.093 | | 0.093 | | 0.093 | | 0.090 | | 0.090 | |
| Validating data (20%) | 0.094 | | 0.094 | | 0.093 | | 0.091 | | 0.091 | |
| Baseline | 0.087 | | 0.087 | | 0.088 | | 0.087 | | 0.088 | |
| 1-year | 0.093 | | 0.093 | | 0.093 | | 0.090 | | 0.090 | |
| 2-year | 0.097 | | 0.096 | | 0.096 | | 0.093 | | 0.093 | |
| 5-year | 0.100 | | 0.099 | | 0.099 | | 0.096 | | 0.095 | |
| RMSE | | | | | | | | | | |
| Training data (80%) | 0.118 | | 0.118 | | 0.118 | | 0.115 | | 0.115 | |
| Validating data (20%) | 0.118 | | 0.118 | | 0.118 | | 0.115 | | 0.115 | |
| Baseline | 0.111 | | 0.111 | | 0.112 | | 0.110 | | 0.111 | |
| 1-year | 0.118 | | 0.118 | | 0.118 | | 0.115 | | 0.115 | |
| 2-year | 0.123 | | 0.122 | | 0.122 | | 0.119 | | 0.119 | |
| 5-year | 0.129 | | 0.127 | | 0.127 | | 0.123 | | 0.123 | |
| RAE | | | | | | | | | | |
| Training data (80%) | 0.823 | | 0.819 | | 0.819 | | 0.796 | | 0.796 | |
| Validating data (20%) | 0.822 | | 0.817 | | 0.817 | | 0.797 | | 0.797 | |
| Baseline | 0.823 | | 0.823 | | 0.834 | | 0.824 | | 0.829 | |
| 1-year | 0.823 | | 0.819 | | 0.818 | | 0.796 | | 0.796 | |
| 2-year | 0.800 | | 0.796 | | 0.797 | | 0.771 | | 0.771 | |
| 5-year | 0.777 | | 0.770 | | 0.770 | | 0.744 | | 0.744 | |
| RRSE | | | | | | | | | | |
| Training data (80%) | 0.859 | | 0.856 | | 0.855 | | 0.835 | | 0.835 | |
| Validating data (20%) | 0.854 | | 0.850 | | 0.850 | | 0.831 | | 0.831 | |
| Baseline | 0.856 | | 0.857 | | 0.862 | | 0.855 | | 0.857 | |
| 1-year | 0.858 | | 0.855 | | 0.854 | | 0.834 | | 0.834 | |
| 2-year | 0.844 | | 0.841 | | 0.841 | | 0.817 | | 0.818 | |
| 5-year | 0.830 | | 0.825 | | 0.825 | | 0.801 | | 0.800 | |

**Mapping algorithm based on 2-year follow-up data**

| | Model 1 | | Model 2 | | Model 3 | | Model 4 | | Model 5 | |
|---|---|---|---|---|---|---|---|---|---|---|
| | coefficient | p-Value | coefficient | p-Value | coefficient | p-Value | coefficient | p-Value | coefficient | p-Value |
| **Intercept** | 0.531 | 0.000 | 0.539 | 0.000 | 0.542 | 0.000 | 0.565 | 0.000 | 0.567 | 0.000 |
| **OP summary score (transformed)** | 0.318 | 0.000 | 0.321 | 0.000 | 0.319 | 0.000 | 0.301 | 0.000 | 0.299 | 0.000 |
| **Log scale, Tobit model** | - | - | - | - | - | - | - | - | - | - |
| **phi (beta regression)** | | | | | | | | | | |
| *phi_Intercept* | - | - | - | - | - | - | - | - | - | - |
| *phi_OPS_transform* | - | - | - | - | - | - | - | - | - | - |
| *Patient characteristics* | - | - | - | - | - | - | - | - | - | - |
| *Age* [a] | | | | | | | | | | |
| 36-45 yrs | - | - | -0.013 | 0.000 | -0.013 | 0.000 | -0.007 | 0.010 | -0.008 | 0.008 |
| 46-55 yrs | - | - | -0.019 | 0.000 | -0.018 | 0.000 | -0.010 | 0.001 | -0.010 | 0.001 |
| 56-65 yrs | - | - | -0.026 | 0.000 | -0.026 | 0.000 | -0.016 | 0.000 | -0.017 | 0.000 |
| 65+ | - | - | -0.021 | 0.007 | -0.023 | 0.005 | -0.012 | 0.120 | -0.013 | 0.104 |
| Man [b] | - | - | 0.012 | 0.000 | 0.012 | 0.000 | 0.009 | 0.001 | 0.009 | 0.000 |
| *BMI* [c] | | | | | | | | | | |
| 40-44 | - | - | - | - | -0.026 | 0.002 | - | - | -0.019 | 0.021 |
| 45-49 | - | - | - | - | -0.024 | 0.167 | - | - | -0.014 | 0.388 |
| 50+ | - | - | - | - | -0.101 | 0.003 | - | - | -0.105 | 0.001 |
| *Comobidity* [d] | | | | | | | | | | |
| Sleep apnea | - | - | - | - | - | - | -0.019 | 0.002 | -0.017 | 0.007 |
| Hypertension | - | - | - | - | - | - | -0.008 | 0.006 | -0.008 | 0.008 |
| Diabetes | - | - | - | - | - | - | -0.009 | 0.058 | -0.010 | 0.049 |
| Dyslipidemia | - | - | - | - | - | - | -0.005 | 0.276 | -0.005 | 0.298 |
| Depression | - | - | - | - | - | - | -0.080 | 0.000 | -0.079 | 0.000 |
| **Goodness of fit** | | | | | | | | | | |
| Adj-Rsq, or pseudo R2 | 0.289 | | 0.287 | | 0.327 | | 0.326 | | 0.322 | |
| AIC | -18957 | | -19032 | | -18657 | | -19477 | | -19419 | |
| BIC | -18934 | | -18972 | | -18575 | | -19379 | | -19299 | |
| **Validation** | | | | | | | | | | |
| *MAE* | | | | | | | | | | |
| Training data (80%) | 0.098 | | 0.098 | | 0.097 | | 0.094 | | 0.094 | |
| Validating data (20%) | 0.098 | | 0.098 | | 0.097 | | 0.094 | | 0.094 | |
| Baseline | 0.088 | | 0.088 | | 0.092 | | 0.088 | | 0.092 | |
| 1-year | 0.095 | | 0.095 | | 0.095 | | 0.092 | | 0.092 | |
| 2-year | 0.098 | | 0.098 | | 0.097 | | 0.094 | | 0.094 | |
| 5-year | 0.100 | | 0.099 | | 0.099 | | 0.096 | | 0.095 | |
| RMSE | | | | | | | | | | |
| Training data (80%) | 0.123 | | 0.122 | | 0.122 | | 0.119 | | 0.119 | |
| Validating data (20%) | 0.122 | | 0.122 | | 0.121 | | 0.118 | | 0.118 | |
| Baseline | 0.111 | | 0.112 | | 0.117 | | 0.111 | | 0.116 | |
| 1-year | 0.119 | | 0.118 | | 0.118 | | 0.116 | | 0.116 | |
| 2-year | 0.123 | | 0.122 | | 0.122 | | 0.119 | | 0.119 | |
| 5-year | 0.127 | | 0.126 | | 0.126 | | 0.122 | | 0.122 | |
| RAE | | | | | | | | | | |
| Training data (80%) | 0.803 | | 0.800 | | 0.802 | | 0.775 | | 0.775 | |
| Validating data (20%) | 0.792 | | 0.790 | | 0.787 | | 0.758 | | 0.758 | |
| Baseline | 0.828 | | 0.829 | | 0.858 | | 0.831 | | 0.859 | |
| 1-year | 0.824 | | 0.821 | | 0.821 | | 0.798 | | 0.798 | |
| 2-year | 0.801 | | 0.798 | | 0.799 | | 0.771 | | 0.772 | |
| 5-year | 0.774 | | 0.770 | | 0.771 | | 0.741 | | 0.741 | |
| RRSE | | | | | | | | | | |
| Training data (80%) | 0.846 | | 0.843 | | 0.844 | | 0.820 | | 0.820 | |
| Validating data (20%) | 0.832 | | 0.830 | | 0.827 | | 0.802 | | 0.802 | |
| Baseline | 0.857 | | 0.858 | | 0.877 | | 0.858 | | 0.878 | |
| 1-year | 0.858 | | 0.855 | | 0.855 | | 0.835 | | 0.836 | |
| 2-year | 0.843 | | 0.841 | | 0.840 | | 0.817 | | 0.817 | |
| 5-year | 0.827 | | 0.822 | | 0.823 | | 0.798 | | 0.797 | |

**Mapping algorithm based on 5-year follow-up data**

| | Model 1 | | Model 2 | | Model 3 | | Model 4 | | Model 5 | |
|---|---|---|---|---|---|---|---|---|---|---|
| | coefficient | p-Value | coefficient | p-Value | coefficient | p-Value | coefficient | p-Value | coefficient | p-Value |
| **Intercept** | 0.506 | 0.000 | 0.521 | 0.000 | 0.523 | 0.000 | 0.556 | 0.000 | 0.557 | 0.000 |
| **OP summary score (transformed)** | 0.323 | 0.000 | 0.325 | 0.000 | 0.324 | 0.000 | 0.298 | 0.000 | 0.297 | 0.000 |
| **Log scale, Tobit model** | - | - | - | - | - | - | - | - | - | - |
| **phi (beta regression)** | | | | | | | | | | |
| *phi_Intercept* | - | - | - | - | - | - | - | - | - | - |
| *phi_OPS_transform* | - | - | - | - | - | - | - | - | - | - |
| *Patient characteristics* | - | - | - | - | - | - | - | - | - | - |
| *Age* [a] | | | | | | | | | | |
| 36-45 yrs | - | - | -0.019 | 0.000 | -0.020 | 0.000 | -0.017 | 0.001 | -0.016 | 0.002 |
| 46-55 yrs | - | - | -0.023 | 0.000 | -0.023 | 0.000 | -0.017 | 0.001 | -0.016 | 0.001 |
| 56-65 yrs | - | - | -0.033 | 0.000 | -0.033 | 0.000 | -0.025 | 0.000 | -0.026 | 0.000 |
| 65+ | - | - | -0.031 | 0.000 | -0.032 | 0.000 | -0.026 | 0.003 | -0.026 | 0.002 |
| Man [b] | - | - | 0.020 | 0.000 | 0.021 | 0.000 | 0.017 | 0.000 | 0.017 | 0.000 |
| *BMI* [c] | | | | | | | | | | |
| 40-44 | - | - | - | - | -0.017 | 0.081 | - | - | -0.013 | 0.175 |
| 45-49 | - | - | - | - | -0.017 | 0.372 | - | - | -0.010 | 0.586 |
| 50+ | - | - | - | - | 0.055 | 0.165 | - | - | 0.062 | 0.107 |
| *Comobidity* [d] | | | | | | | | | | |
| Sleep apnea | - | - | - | - | - | - | 0.000 | 0.976 | 0.000 | 0.991 |
| Hypertension | - | - | - | - | - | - | -0.008 | 0.058 | -0.008 | 0.058 |
| Diabetes | - | - | - | - | - | - | -0.013 | 0.079 | -0.011 | 0.122 |
| Dyslipidemia | - | - | - | - | - | - | 0.000 | 0.982 | 0.001 | 0.908 |
| Depression | - | - | - | - | - | - | -0.086 | 0.000 | -0.086 | 0.000 |
| **Goodness of fit** | | | | | | | | | | |
| Adj-Rsq, or pseudo R2 | 0.329 | | 0.328 | | 0.371 | | 0.373 | | -0.264 | |
| AIC | -7840 | | -7890 | | -7625 | | -8045 | | -8015 | |
| BIC | -7820 | | -7837 | | -7552 | | -7959 | | -7909 | |
| **Validation** | | | | | | | | | | |
| *MAE* | | | | | | | | | | |
| Training data (80%) | 0.101 | | 0.101 | | 0.100 | | 0.096 | | 0.096 | |
| Validating data (20%) | 0.101 | | 0.100 | | 0.100 | | 0.098 | | 0.098 | |
| Baseline | 0.093 | | 0.092 | | 0.094 | | 0.091 | | 0.092 | |
| 1-year | 0.101 | | 0.099 | | 0.099 | | 0.096 | | 0.096 | |
| 2-year | 0.102 | | 0.101 | | 0.101 | | 0.098 | | 0.098 | |
| 5-year | 0.101 | | 0.101 | | 0.100 | | 0.097 | | 0.096 | |
| RMSE | | | | | | | | | | |
| Training data (80%) | 0.125 | | 0.125 | | 0.124 | | 0.121 | | 0.120 | |
| Validating data (20%) | 0.125 | | 0.124 | | 0.124 | | 0.121 | | 0.121 | |
| Baseline | 0.118 | | 0.117 | | 0.119 | | 0.115 | | 0.117 | |
| 1-year | 0.122 | | 0.121 | | 0.121 | | 0.118 | | 0.118 | |
| 2-year | 0.124 | | 0.124 | | 0.123 | | 0.120 | | 0.120 | |
| 5-year | 0.125 | | 0.125 | | 0.124 | | 0.121 | | 0.120 | |
| RAE | | | | | | | | | | |
| Training data (80%) | 0.778 | | 0.773 | | 0.773 | | 0.742 | | 0.741 | |
| Validating data (20%) | 0.768 | | 0.764 | | 0.764 | | 0.747 | | 0.746 | |
| Baseline | 0.858 | | 0.853 | | 0.873 | | 0.852 | | 0.867 | |
| 1-year | 0.833 | | 0.827 | | 0.827 | | 0.805 | | 0.805 | |
| 2-year | 0.809 | | 0.805 | | 0.806 | | 0.779 | | 0.780 | |
| 5-year | 0.776 | | 0.771 | | 0.771 | | 0.743 | | 0.742 | |
| RRSE | | | | | | | | | | |
| Training data (80%) | 0.823 | | 0.819 | | 0.819 | | 0.792 | | 0.791 | |
| Validating data (20%) | 0.823 | | 0.818 | | 0.820 | | 0.803 | | 0.803 | |
| Baseline | 0.870 | | 0.871 | | 0.887 | | 0.871 | | 0.886 | |
| 1-year | 0.865 | | 0.861 | | 0.861 | | 0.842 | | 0.842 | |
| 2-year | 0.847 | | 0.844 | | 0.844 | | 0.821 | | 0.821 | |
| 5-year | 0.823 | | 0.819 | | 0.819 | | 0.794 | | 0.793 | |

**Reference group**
**a 18-34 years**
**b Women**
**c <=39**
**d No disease**

# Table S6A. Mapping algorithm based on Tobit models, Type A,  transfored SF-6D index applied

| | Mapping algorithm based on baseline data | | | | | | | | | |
|---|---|---|---|---|---|---|---|---|---|---|
| | Model 1 | | Model 2 | | Model 3 | | Model 4 | | Model 5 | |
| | coefficient | p-Value | coefficient | p-Value | coefficient | p-Value | coefficient | p-Value | coefficient | p-Value |
| **Intercept** | 0.570 | 0.000 | 0.573 | 0.000 | 0.580 | 0.000 | 0.580 | 0.000 | 0.586 | 0.000 |
| **OP summary score (transformed)** | 0.262 | 0.000 | 0.268 | 0.000 | 0.268 | 0.000 | 0.262 | 0.000 | 0.262 | 0.000 |
| **Log scale, Tobit model** | -2.212 | 0.000 | -2.214 | 0.000 | -2.216 | 0.000 | -2.222 | 0.000 | -2.223 | 0.000 |
| **phi (beta regression)** | | | | | | | | | | |
| *phi_Intercept* | | | | | | | | | | |
| *phi_OPS_transform* | | | | | | | | | | |
| *Patient characteristics* | | | | | | | | | | |
| *Age* [a] | | | | | | | | | | |
| 36-45 yrs | - | - | 0.001 | 0.557 | 0.000 | 0.807 | 0.004 | 0.010 | 0.003 | 0.086 |
| 46-55 yrs | - | - | -0.009 | 0.000 | -0.011 | 0.000 | -0.003 | 0.105 | -0.005 | 0.005 |
| 56-65 yrs | - | - | -0.017 | 0.000 | -0.019 | 0.000 | -0.008 | 0.002 | -0.010 | 0.000 |
| 65+ | - | - | -0.018 | 0.032 | -0.021 | 0.015 | -0.008 | 0.327 | -0.011 | 0.191 |
| Man [b] | - | - | -0.007 | 0.000 | -0.005 | 0.001 | -0.005 | 0.001 | -0.004 | 0.021 |
| *BMI* [c] | | | | | | | | | | |
| 40-44 | - | - | - | - | -0.007 | 0.000 | - | - | -0.007 | 0.000 |
| 45-49 | - | - | - | - | -0.012 | 0.000 | - | - | -0.012 | 0.000 |
| 50+ | - | - | - | - | -0.022 | 0.000 | - | - | -0.020 | 0.000 |
| *Comobidity* [d] | | | | | | | | | | |
| Sleep apnea | - | - | - | - | - | - | -0.015 | 0.000 | -0.014 | 0.000 |
| Hypertension | - | - | - | - | - | - | -0.002 | 0.273 | -0.001 | 0.546 |
| Diabetes | - | - | - | - | - | - | -0.009 | 0.000 | -0.010 | 0.000 |
| Dyslipidemia | - | - | - | - | - | - | -0.003 | 0.181 | -0.004 | 0.095 |
| Depression | - | - | - | - | - | - | -0.033 | 0.000 | -0.033 | 0.000 |
| **Goodness of fit** | | | | | | | | | | |
| Adj-Rsq, or pseudo R2 | - | | - | | - | | - | | - | |
| AIC | -46040 | | -46143 | | -46235 | | -46596 | | -46677 | |
| BIC | -46015 | | -46077 | | -46144 | | -46488 | | -46545 | |
| **Validation** | | | | | | | | | | |
| *MAE* | | | | | | | | | | |
| Training data (80%) | 0.087 | | 0.087 | | 0.087 | | 0.086 | | 0.086 | |
| Validating data (20%) | 0.085 | | 0.085 | | 0.085 | | 0.084 | | 0.084 | |
| Baseline | 0.087 | | 0.087 | | 0.086 | | 0.086 | | 0.086 | |
| 1-year | 0.099 | | 0.098 | | 0.096 | | 0.096 | | 0.095 | |
| 2-year | 0.101 | | 0.101 | | 0.100 | | 0.098 | | 0.097 | |
| 5-year | 0.103 | | 0.102 | | 0.102 | | 0.100 | | 0.100 | |
| RMSE | | | | | | | | | | |
| Training data (80%) | 0.109 | | 0.109 | | 0.109 | | 0.108 | | 0.108 | |
| Validating data (20%) | 0.107 | | 0.107 | | 0.106 | | 0.106 | | 0.106 | |
| Baseline | 0.109 | | 0.109 | | 0.108 | | 0.108 | | 0.108 | |
| 1-year | 0.120 | | 0.120 | | 0.119 | | 0.118 | | 0.117 | |
| 2-year | 0.124 | | 0.123 | | 0.123 | | 0.121 | | 0.121 | |
| 5-year | 0.127 | | 0.127 | | 0.127 | | 0.124 | | 0.125 | |
| RAE | | | | | | | | | | |
| Training data (80%) | 0.823 | | 0.820 | | 0.819 | | 0.814 | | 0.813 | |
| Validating data (20%) | 0.809 | | 0.806 | | 0.805 | | 0.797 | | 0.796 | |
| Baseline | 0.820 | | 0.818 | | 0.816 | | 0.810 | | 0.809 | |
| 1-year | 0.843 | | 0.840 | | 0.837 | | 0.824 | | 0.821 | |
| 2-year | 0.822 | | 0.819 | | 0.818 | | 0.801 | | 0.800 | |
| 5-year | 0.795 | | 0.793 | | 0.793 | | 0.776 | | 0.775 | |
| RRSE | | | | | | | | | | |
| Training data (80%) | 0.849 | | 0.847 | | 0.846 | | 0.840 | | 0.839 | |
| Validating data (20%) | 0.836 | | 0.833 | | 0.832 | | 0.826 | | 0.825 | |
| Baseline | 0.846 | | 0.844 | | 0.843 | | 0.838 | | 0.836 | |
| 1-year | 0.865 | | 0.863 | | 0.862 | | 0.849 | | 0.847 | |
| 2-year | 0.849 | | 0.849 | | 0.848 | | 0.832 | | 0.832 | |
| 5-year | 0.832 | | 0.831 | | 0.832 | | 0.815 | | 0.815 | |

| | Mapping algorithm based on 1-year follow-up data | | | | | | | | | |
|---|---|---|---|---|---|---|---|---|---|---|
| | Model 1 | | Model 2 | | Model 3 | | Model 4 | | Model 5 | |
| | coefficient | p-Value | coefficient | p-Value | coefficient | p-Value | coefficient | p-Value | coefficient | p-Value |
| **Intercept** | 0.530 | 0.000 | 0.536 | 0.000 | 0.537 | 0.000 | 0.561 | 0.000 | 0.562 | 0.000 |
| **OP summary score (transformed)** | 0.336 | 0.000 | 0.338 | 0.000 | 0.338 | 0.000 | 0.320 | 0.000 | 0.319 | 0.000 |
| **Log scale, Tobit model** | -2.075 | 0.000 | -2.079 | 0.000 | -2.080 | 0.000 | -2.104 | 0.000 | -2.104 | 0.000 |
| **phi (beta regression)** | | | | | | | | | | |
| *phi_Intercept* | | | | | | | | | | |
| *phi_OPS_transform* | | | | | | | | | | |
| *Patient characteristics* | | | | | | | | | | |
| *Age* [a] | | | | | | | | | | |
| 36-45 yrs | - | - | -0.009 | 0.000 | -0.009 | 0.000 | -0.005 | 0.037 | -0.005 | 0.034 |
| 46-55 yrs | - | - | -0.017 | 0.000 | -0.017 | 0.000 | -0.010 | 0.000 | -0.010 | 0.000 |
| 56-65 yrs | - | - | -0.027 | 0.000 | -0.028 | 0.000 | -0.018 | 0.000 | -0.018 | 0.000 |
| 65+ | - | - | -0.019 | 0.028 | -0.019 | 0.032 | -0.009 | 0.300 | -0.009 | 0.284 |
| Man [b] | - | - | 0.012 | 0.000 | 0.012 | 0.000 | 0.009 | 0.000 | 0.010 | 0.000 |
| *BMI* [c] | | | | | | | | | | |
| 40-44 | - | - | - | - | -0.017 | 0.013 | - | - | -0.010 | 0.140 |
| 45-49 | - | - | - | - | -0.034 | 0.031 | - | - | -0.024 | 0.127 |
| 50+ | - | - | - | - | 0.009 | 0.718 | - | - | 0.011 | 0.651 |
| *Comobidity* [d] | | | | | | | | | | |
| Sleep apnea | - | - | - | - | - | - | -0.021 | 0.000 | -0.021 | 0.000 |
| Hypertension | - | - | - | - | - | - | -0.011 | 0.000 | -0.011 | 0.000 |
| Diabetes | - | - | - | - | - | - | -0.013 | 0.004 | -0.012 | 0.004 |
| Dyslipidemia | - | - | - | - | - | - | 0.000 | 0.899 | 0.000 | 0.899 |
| Depression | - | - | - | - | - | - | -0.075 | 0.000 | -0.075 | 0.000 |
| **Goodness of fit** | | | | | | | | | | |
| Adj-Rsq, or pseudo R2 | - | | - | | - | | - | | - | |
| AIC | -22972 | | -23092 | | -22930 | | -23916 | | -23900 | |
| BIC | -22948 | | -23028 | | -22843 | | -23812 | | -23772 | |
| **Validation** | | | | | | | | | | |
| *MAE* | | | | | | | | | | |
| Training data (80%) | 0.092 | | 0.092 | | 0.092 | | 0.089 | | 0.089 | |
| Validating data (20%) | 0.093 | | 0.092 | | 0.092 | | 0.090 | | 0.090 | |
| Baseline | 0.087 | | 0.087 | | 0.089 | | 0.088 | | 0.088 | |
| 1-year | 0.092 | | 0.092 | | 0.092 | | 0.089 | | 0.089 | |
| 2-year | 0.096 | | 0.096 | | 0.095 | | 0.092 | | 0.092 | |
| 5-year | 0.100 | | 0.099 | | 0.099 | | 0.096 | | 0.095 | |
| RMSE | | | | | | | | | | |
| Training data (80%) | 0.118 | | 0.118 | | 0.118 | | 0.115 | | 0.115 | |
| Validating data (20%) | 0.118 | | 0.118 | | 0.118 | | 0.115 | | 0.115 | |
| Baseline | 0.111 | | 0.111 | | 0.113 | | 0.111 | | 0.112 | |
| 1-year | 0.118 | | 0.118 | | 0.118 | | 0.115 | | 0.115 | |
| 2-year | 0.123 | | 0.123 | | 0.122 | | 0.119 | | 0.119 | |
| 5-year | 0.129 | | 0.128 | | 0.128 | | 0.124 | | 0.124 | |
| RAE | | | | | | | | | | |
| Training data (80%) | 0.822 | | 0.818 | | 0.817 | | 0.794 | | 0.794 | |
| Validating data (20%) | 0.820 | | 0.815 | | 0.815 | | 0.795 | | 0.795 | |
| Baseline | 0.827 | | 0.827 | | 0.839 | | 0.829 | | 0.835 | |
| 1-year | 0.821 | | 0.817 | | 0.817 | | 0.794 | | 0.794 | |
| 2-year | 0.798 | | 0.794 | | 0.794 | | 0.768 | | 0.768 | |
| 5-year | 0.777 | | 0.769 | | 0.769 | | 0.744 | | 0.743 | |
| RRSE | | | | | | | | | | |
| Training data (80%) | 0.859 | | 0.856 | | 0.855 | | 0.835 | | 0.835 | |
| Validating data (20%) | 0.853 | | 0.850 | | 0.850 | | 0.831 | | 0.831 | |
| Baseline | 0.861 | | 0.861 | | 0.867 | | 0.861 | | 0.863 | |
| 1-year | 0.858 | | 0.855 | | 0.854 | | 0.835 | | 0.835 | |
| 2-year | 0.845 | | 0.842 | | 0.842 | | 0.818 | | 0.819 | |
| 5-year | 0.832 | | 0.826 | | 0.827 | | 0.803 | | 0.802 | |

| | Mapping algorithm based on 2-year follow-up data | | | | | | | | | |
|---|---|---|---|---|---|---|---|---|---|---|
| | Model 1 | | Model 2 | | Model 3 | | Model 4 | | Model 5 | |
| | coefficient | p-Value | coefficient | p-Value | coefficient | p-Value | coefficient | p-Value | coefficient | p-Value |
| **Intercept** | 0.526 | 0.000 | 0.533 | 0.000 | 0.536 | 0.000 | 0.560 | 0.000 | 0.562 | 0.000 |
| **OP summary score (transformed)** | 0.329 | 0.000 | 0.332 | 0.000 | 0.329 | 0.000 | 0.311 | 0.000 | 0.309 | 0.000 |
| **Log scale, Tobit model** | -2.049 | 0.000 | -2.052 | 0.000 | -2.053 | 0.000 | -2.081 | 0.000 | -2.082 | 0.000 |
| **phi (beta regression)** | | | | | | | | | | |
| *phi_Intercept* | | | | | | | | | | |
| *phi_OPS_transform* | | | | | | | | | | |
| *Patient characteristics* | | | | | | | | | | |
| *Age* [a] | | | | | | | | | | |
| 36-45 yrs | - | - | -0.012 | 0.000 | -0.012 | 0.000 | -0.006 | 0.035 | -0.007 | 0.031 |
| 46-55 yrs | - | - | -0.018 | 0.000 | -0.018 | 0.000 | -0.009 | 0.006 | -0.009 | 0.005 |
| 56-65 yrs | - | - | -0.026 | 0.000 | -0.026 | 0.000 | -0.015 | 0.000 | -0.015 | 0.000 |
| 65+ | - | - | -0.022 | 0.010 | -0.023 | 0.007 | -0.012 | 0.154 | -0.013 | 0.136 |
| Man [b] | - | - | 0.013 | 0.000 | 0.013 | 0.000 | 0.010 | 0.000 | 0.010 | 0.000 |
| *BMI* [c] | | | | | | | | | | |
| 40-44 | - | - | - | - | -0.027 | 0.002 | - | - | -0.019 | 0.022 |
| 45-49 | - | - | - | - | -0.024 | 0.185 | - | - | -0.014 | 0.416 |
| 50+ | - | - | - | - | -0.103 | 0.004 | - | - | -0.106 | 0.002 |
| *Comobidity* [d] | | | | | | | | | | |
| Sleep apnea | - | - | - | - | - | - | -0.021 | 0.001 | -0.019 | 0.004 |
| Hypertension | - | - | - | - | - | - | -0.009 | 0.005 | -0.009 | 0.007 |
| Diabetes | - | - | - | - | - | - | -0.010 | 0.059 | -0.010 | 0.048 |
| Dyslipidemia | - | - | - | - | - | - | -0.005 | 0.302 | -0.005 | 0.328 |
| Depression | - | - | - | - | - | - | -0.082 | 0.000 | -0.082 | 0.000 |
| **Goodness of fit** | | | | | | | | | | |
| Adj-Rsq, or pseudo R2 | - | | - | | - | | - | | - | |
| AIC | -14518 | | -14587 | | -14253 | | -15028 | | -14980 | |
| BIC | -14496 | | -14526 | | -14170 | | -14930 | | -14860 | |
| **Validation** | | | | | | | | | | |
| *MAE* | | | | | | | | | | |
| Training data (80%) | 0.097 | | 0.097 | | 0.096 | | 0.093 | | 0.093 | |
| Validating data (20%) | 0.097 | | 0.097 | | 0.096 | | 0.093 | | 0.093 | |
| Baseline | 0.088 | | 0.088 | | 0.093 | | 0.088 | | 0.092 | |
| 1-year | 0.094 | | 0.094 | | 0.094 | | 0.091 | | 0.091 | |
| 2-year | 0.097 | | 0.097 | | 0.096 | | 0.093 | | 0.093 | |
| 5-year | 0.100 | | 0.099 | | 0.099 | | 0.095 | | 0.095 | |
| RMSE | | | | | | | | | | |
| Training data (80%) | 0.123 | | 0.122 | | 0.122 | | 0.119 | | 0.119 | |
| Validating data (20%) | 0.122 | | 0.122 | | 0.121 | | 0.118 | | 0.118 | |
| Baseline | 0.112 | | 0.112 | | 0.118 | | 0.112 | | 0.117 | |
| 1-year | 0.119 | | 0.118 | | 0.118 | | 0.115 | | 0.115 | |
| 2-year | 0.123 | | 0.122 | | 0.122 | | 0.118 | | 0.118 | |
| 5-year | 0.127 | | 0.126 | | 0.126 | | 0.122 | | 0.122 | |
| RAE | | | | | | | | | | |
| Training data (80%) | 0.801 | | 0.797 | | 0.799 | | 0.772 | | 0.772 | |
| Validating data (20%) | 0.789 | | 0.786 | | 0.783 | | 0.755 | | 0.755 | |
| Baseline | 0.831 | | 0.832 | | 0.863 | | 0.835 | | 0.863 | |
| 1-year | 0.821 | | 0.818 | | 0.818 | | 0.795 | | 0.796 | |
| 2-year | 0.798 | | 0.795 | | 0.796 | | 0.769 | | 0.769 | |
| 5-year | 0.772 | | 0.767 | | 0.768 | | 0.740 | | 0.740 | |
| RRSE | | | | | | | | | | |
| Training data (80%) | 0.846 | | 0.844 | | 0.844 | | 0.820 | | 0.821 | |
| Validating data (20%) | 0.832 | | 0.829 | | 0.826 | | 0.802 | | 0.802 | |
| Baseline | 0.860 | | 0.862 | | 0.881 | | 0.863 | | 0.882 | |
| 1-year | 0.858 | | 0.855 | | 0.855 | | 0.835 | | 0.836 | |
| 2-year | 0.843 | | 0.841 | | 0.841 | | 0.817 | | 0.817 | |
| 5-year | 0.828 | | 0.823 | | 0.824 | | 0.799 | | 0.799 | |

| | Mapping algorithm based on 5-year follow-up data | | | | | | | | | |
|---|---|---|---|---|---|---|---|---|---|---|
| | Model 1 | | Model 2 | | Model 3 | | Model 4 | | Model 5 | |
| | coefficient | p-Value | coefficient | p-Value | coefficient | p-Value | coefficient | p-Value | coefficient | p-Value |
| **Intercept** | 0.502 | 0.000 | 0.517 | 0.000 | 0.519 | 0.000 | 0.552 | 0.000 | 0.554 | 0.000 |
| **OP summary score (transformed)** | 0.331 | 0.000 | 0.333 | 0.000 | 0.332 | 0.000 | 0.305 | 0.000 | 0.304 | 0.000 |
| **Log scale, Tobit model** | -2.042 | 0.000 | -2.047 | 0.000 | -2.050 | 0.000 | -2.081 | 0.000 | -2.085 | 0.000 |
| **phi (beta regression)** | | | | | | | | | | |
| *phi_Intercept* | | | | | | | | | | |
| *phi_OPS_transform* | | | | | | | | | | |
| *Patient characteristics* | | | | | | | | | | |
| *Age* [a] | | | | | | | | | | |
| 36-45 yrs | - | - | -0.019 | 0.001 | -0.020 | 0.000 | -0.017 | 0.002 | -0.016 | 0.002 |
| 46-55 yrs | - | - | -0.022 | 0.000 | -0.022 | 0.000 | -0.016 | 0.002 | -0.016 | 0.003 |
| 56-65 yrs | - | - | -0.032 | 0.000 | -0.033 | 0.000 | -0.024 | 0.000 | -0.025 | 0.000 |
| 65+ | - | - | -0.031 | 0.001 | -0.031 | 0.000 | -0.024 | 0.007 | -0.025 | 0.005 |
| Man [b] | - | - | 0.020 | 0.000 | 0.022 | 0.000 | 0.017 | 0.000 | 0.018 | 0.000 |
| *BMI* [c] | | | | | | | | | | |
| 40-44 | - | - | - | - | -0.018 | 0.086 | - | - | -0.013 | 0.184 |
| 45-49 | - | - | - | - | -0.018 | 0.371 | - | - | -0.011 | 0.586 |
| 50+ | - | - | - | - | 0.054 | 0.188 | - | - | 0.061 | 0.122 |
| *Comobidity* [d] | | | | | | | | | | |
| Sleep apnea | - | - | - | - | - | - | 0.000 | 0.983 | 0.000 | 0.971 |
| Hypertension | - | - | - | - | - | - | -0.009 | 0.048 | -0.009 | 0.050 |
| Diabetes | - | - | - | - | - | - | -0.013 | 0.071 | -0.012 | 0.109 |
| Dyslipidemia | - | - | - | - | - | - | 0.000 | 0.985 | 0.001 | 0.875 |
| Depression | - | - | - | - | - | - | -0.088 | 0.000 | -0.088 | 0.000 |
| **Goodness of fit** | | | | | | | | | | |
| Adj-Rsq, or pseudo R2 | - | | - | | - | | - | | - | |
| AIC | -6464 | | -6510 | | -6275 | | -6670 | | -6652 | |
| BIC | -6444 | | -6456 | | -6202 | | -6583 | | -6546 | |
| **Validation** | | | | | | | | | | |
| *MAE* | | | | | | | | | | |
| Training data (80%) | 0.101 | | 0.100 | | 0.100 | | 0.096 | | 0.096 | |
| Validating data (20%) | 0.100 | | 0.100 | | 0.100 | | 0.097 | | 0.097 | |
| Baseline | 0.093 | | 0.092 | | 0.095 | | 0.091 | | 0.093 | |
| 1-year | 0.100 | | 0.098 | | 0.098 | | 0.095 | | 0.095 | |
| 2-year | 0.101 | | 0.100 | | 0.100 | | 0.097 | | 0.097 | |
| 5-year | 0.101 | | 0.100 | | 0.100 | | 0.096 | | 0.096 | |
| RMSE | | | | | | | | | | |
| Training data (80%) | 0.125 | | 0.125 | | 0.124 | | 0.120 | | 0.120 | |
| Validating data (20%) | 0.125 | | 0.124 | | 0.124 | | 0.122 | | 0.122 | |
| Baseline | 0.118 | | 0.117 | | 0.120 | | 0.115 | | 0.117 | |
| 1-year | 0.122 | | 0.120 | | 0.120 | | 0.117 | | 0.117 | |
| 2-year | 0.124 | | 0.123 | | 0.123 | | 0.120 | | 0.120 | |
| 5-year | 0.125 | | 0.125 | | 0.124 | | 0.121 | | 0.120 | |
| RAE | | | | | | | | | | |
| Training data (80%) | 0.775 | | 0.770 | | 0.770 | | 0.739 | | 0.738 | |
| Validating data (20%) | 0.765 | | 0.761 | | 0.761 | | 0.744 | | 0.743 | |
| Baseline | 0.861 | | 0.856 | | 0.876 | | 0.855 | | 0.870 | |
| 1-year | 0.829 | | 0.824 | | 0.824 | | 0.802 | | 0.802 | |
| 2-year | 0.806 | | 0.802 | | 0.803 | | 0.776 | | 0.777 | |
| 5-year | 0.773 | | 0.768 | | 0.768 | | 0.740 | | 0.739 | |
| RRSE | | | | | | | | | | |
| Training data (80%) | 0.823 | | 0.819 | | 0.819 | | 0.792 | | 0.791 | |
| Validating data (20%) | 0.824 | | 0.818 | | 0.820 | | 0.804 | | 0.803 | |
| Baseline | 0.873 | | 0.874 | | 0.889 | | 0.874 | | 0.889 | |
| 1-year | 0.863 | | 0.860 | | 0.860 | | 0.841 | | 0.841 | |
| 2-year | 0.846 | | 0.843 | | 0.844 | | 0.820 | | 0.821 | |
| 5-year | 0.823 | | 0.819 | | 0.820 | | 0.795 | | 0.794 | |

**Reference group**
**a 18-34 years**
**b Women**
**c <=39**
**d No disease**

# Table S7A. Mapping algorithm based on beta regression models, Type A, transfored SF-6D index applied

**Mapping algorithm based on baseline data**

| | Model 1 | | Model 2 | | Model 3 | | Model 4 | | Model 5 | |
|---|---|---|---|---|---|---|---|---|---|---|
| | coefficient | p-Value | coefficient | p-Value | coefficient | p-Value | coefficient | p-Value | coefficient | p-Value |
| **Intercept** | 0.162 | 0.000 | 0.178 | 0.000 | 0.206 | 0.000 | 0.204 | 0.000 | 0.230 | 0.000 |
| **OP summary score (transformed)** | 1.266 | 0.000 | 1.297 | 0.000 | 1.295 | 0.000 | 1.270 | 0.000 | 1.268 | 0.000 |
| **Log scale, Tobit model** | | | | | | | | | | |
| **phi (beta regression)** | - | - | - | - | - | - | - | - | - | - |
| *phi_Intercept* | 3.016 | 0.000 | 3.024 | 0.000 | 3.029 | 0.000 | 3.036 | 0.000 | 3.041 | 0.000 |
| *phi_OPS_transform* | -0.630 | 0.000 | -0.640 | 0.000 | -0.646 | 0.000 | -0.640 | 0.000 | -0.645 | 0.000 |
| *Patient characteristics* | | | | | | | | | | |
| *Age* [a] | | | | | | | | | | |
| 36-45 yrs | - | - | 0.000 | 0.935 | -0.005 | 0.488 | 0.013 | 0.049 | 0.008 | 0.245 |
| 46-55 yrs | - | - | -0.036 | 0.000 | -0.043 | 0.000 | -0.013 | 0.094 | -0.021 | 0.006 |
| 56-65 yrs | - | - | -0.072 | 0.000 | -0.079 | 0.000 | -0.038 | 0.000 | -0.047 | 0.000 |
| 65+ | - | - | -0.048 | 0.215 | -0.058 | 0.132 | -0.011 | 0.772 | -0.023 | 0.554 |
| Man [b] | - | - | -0.042 | 0.000 | -0.031 | 0.000 | -0.033 | 0.000 | -0.025 | 0.000 |
| *BMI* [c] | | | | | | | | | | |
| 40-44 | - | - | - | - | -0.032 | 0.000 | - | - | -0.031 | 0.000 |
| 45-49 | - | - | - | - | -0.047 | 0.000 | - | - | -0.044 | 0.000 |
| 50+ | - | - | - | - | -0.092 | 0.000 | - | - | -0.083 | 0.000 |
| *Comobidity* [d] | | | | | | | | | | |
| Sleep apnea | - | - | - | - | - | - | -0.064 | 0.000 | -0.057 | 0.000 |
| Hypertension | - | - | - | - | - | - | -0.006 | 0.382 | -0.003 | 0.655 |
| Diabetes | - | - | - | - | - | - | -0.040 | 0.000 | -0.040 | 0.000 |
| Dyslipidemia | - | - | - | - | - | - | -0.011 | 0.297 | -0.014 | 0.191 |
| Depression | - | - | - | - | - | - | -0.109 | 0.000 | -0.108 | 0.000 |
| **Goodness of fit** | | | | | | | | | | |
| Adj-Rsq, or pseudo R2 | 0.012 | | 0.012 | | 0.012 | | 0.012 | | 0.033 | |
| AIC | -46500 | | -46610 | | -46702 | | -46966 | | -47042 | |
| BIC | -46467 | | -46536 | | -46603 | | -46850 | | -46902 | |
| **Validation** | | | | | | | | | | |
| *MAE* | | | | | | | | | | |
| Training data (80%) | 0.087 | | 0.086 | | 0.086 | | 0.086 | | 0.086 | |
| Validating data (20%) | 0.085 | | 0.085 | | 0.084 | | 0.084 | | 0.084 | |
| Baseline | 0.086 | | 0.086 | | 0.086 | | 0.086 | | 0.085 | |
| 1-year | 0.105 | | 0.104 | | 0.104 | | 0.104 | | 0.103 | |
| 2-year | 0.107 | | 0.106 | | 0.106 | | 0.105 | | 0.105 | |
| 5-year | 0.107 | | 0.106 | | 0.106 | | 0.105 | | 0.105 | |
| RMSE | | | | | | | | | | |
| Training data (80%) | 0.108 | | 0.108 | | 0.108 | | 0.108 | | 0.107 | |
| Validating data (20%) | 0.106 | | 0.106 | | 0.106 | | 0.105 | | 0.105 | |
| Baseline | 0.108 | | 0.108 | | 0.108 | | 0.107 | | 0.107 | |
| 1-year | 0.125 | | 0.124 | | 0.124 | | 0.123 | | 0.123 | |
| 2-year | 0.127 | | 0.127 | | 0.126 | | 0.125 | | 0.125 | |
| 5-year | 0.129 | | 0.129 | | 0.129 | | 0.127 | | 0.127 | |
| RAE | | | | | | | | | | |
| Training data (80%) | 0.819 | | 0.815 | | 0.814 | | 0.810 | | 0.809 | |
| Validating data (20%) | 0.804 | | 0.801 | | 0.800 | | 0.794 | | 0.793 | |
| Baseline | 0.816 | | 0.812 | | 0.811 | | 0.807 | | 0.806 | |
| 1-year | 0.873 | | 0.870 | | 0.871 | | 0.862 | | 0.863 | |
| 2-year | 0.852 | | 0.850 | | 0.852 | | 0.842 | | 0.843 | |
| 5-year | 0.824 | | 0.821 | | 0.823 | | 0.812 | | 0.814 | |
| RRSE | | | | | | | | | | |
| Training data (80%) | 0.842 | | 0.840 | | 0.838 | | 0.834 | | 0.833 | |
| Validating data (20%) | 0.829 | | 0.826 | | 0.825 | | 0.820 | | 0.819 | |
| Baseline | 0.839 | | 0.837 | | 0.836 | | 0.831 | | 0.830 | |
| 1-year | 0.885 | | 0.883 | | 0.883 | | 0.875 | | 0.876 | |
| 2-year | 0.867 | | 0.866 | | 0.867 | | 0.857 | | 0.858 | |
| 5-year | 0.848 | | 0.846 | | 0.848 | | 0.837 | | 0.838 | |

**Mapping algorithm based on 1-year follow-up data**

| | Model 1 | | Model 2 | | Model 3 | | Model 4 | | Model 5 | |
|---|---|---|---|---|---|---|---|---|---|---|
| | coefficient | p-Value | coefficient | p-Value | coefficient | p-Value | coefficient | p-Value | coefficient | p-Value |
| **Intercept** | -0.118 | 0.000 | -0.066 | 0.000 | -0.058 | 0.001 | 0.080 | 0.000 | 0.083 | 0.000 |
| **OP summary score (transformed)** | 1.962 | 0.000 | 1.967 | 0.000 | 1.961 | 0.000 | 1.846 | 0.000 | 1.844 | 0.000 |
| **Log scale, Tobit model** | | | | | | | | | | |
| **phi (beta regression)** | - | - | - | - | - | - | - | - | - | - |
| *phi_Intercept* | 3.006 | 0.000 | 3.038 | 0.000 | 3.037 | 0.000 | 3.062 | 0.000 | 3.062 | 0.000 |
| *phi_OPS_transform* | -1.309 | 0.000 | -1.343 | 0.000 | -1.341 | 0.000 | -1.343 | 0.000 | -1.343 | 0.000 |
| *Patient characteristics* | | | | | | | | | | |
| *Age* [a] | | | | | | | | | | |
| 36-45 yrs | - | - | -0.060 | 0.001 | -0.061 | 0.001 | -0.029 | 0.107 | -0.030 | 0.093 |
| 46-55 yrs | - | - | -0.112 | 0.000 | -0.114 | 0.000 | -0.049 | 0.010 | -0.050 | 0.009 |
| 56-65 yrs | - | - | -0.201 | 0.000 | -0.204 | 0.000 | -0.134 | 0.000 | -0.136 | 0.000 |
| 65+ | - | - | -0.095 | 0.171 | -0.089 | 0.204 | -0.022 | 0.748 | -0.024 | 0.736 |
| Man [b] | - | - | 0.090 | 0.000 | 0.093 | 0.000 | 0.080 | 0.000 | 0.080 | 0.000 |
| *BMI* [c] | | | | | | | | | | |
| 40-44 | - | - | - | - | -0.104 | 0.008 | - | - | -0.045 | 0.255 |
| 45-49 | - | - | - | - | -0.154 | 0.055 | - | - | -0.103 | 0.202 |
| 50+ | - | - | - | - | 0.086 | 0.448 | - | - | 0.096 | 0.393 |
| *Comobidity* [d] | | | | | | | | | | |
| Sleep apnea | - | - | - | - | - | - | -0.141 | 0.000 | -0.137 | 0.000 |
| Hypertension | - | - | - | - | - | - | -0.082 | 0.000 | -0.081 | 0.000 |
| Diabetes | - | - | - | - | - | - | -0.104 | 0.001 | -0.102 | 0.001 |
| Dyslipidemia | - | - | - | - | - | - | 0.019 | 0.536 | 0.018 | 0.542 |
| Depression | - | - | - | - | - | - | -0.401 | 0.000 | -0.400 | 0.000 |
| **Goodness of fit** | | | | | | | | | | |
| Adj-Rsq, or pseudo R2 | 0.032 | | 0.032 | | 0.037 | | 0.037 | | 0.032 | |
| AIC | -35079 | | -35172 | | -34923 | | -35526 | | -35503 | |
| BIC | -35047 | | -35100 | | -34828 | | -35414 | | -35368 | |
| **Validation** | | | | | | | | | | |
| *MAE* | | | | | | | | | | |
| Training data (80%) | 0.095 | | 0.095 | | 0.095 | | 0.093 | | 0.093 | |
| Validating data (20%) | 0.096 | | 0.096 | | 0.096 | | 0.095 | | 0.095 | |
| Baseline | 0.091 | | 0.091 | | 0.095 | | 0.093 | | 0.094 | |
| 1-year | 0.095 | | 0.095 | | 0.095 | | 0.093 | | 0.093 | |
| 2-year | 0.099 | | 0.099 | | 0.098 | | 0.097 | | 0.097 | |
| 5-year | 0.102 | | 0.102 | | 0.101 | | 0.100 | | 0.099 | |
| RMSE | | | | | | | | | | |
| Training data (80%) | 0.120 | | 0.120 | | 0.119 | | 0.117 | | 0.117 | |
| Validating data (20%) | 0.120 | | 0.120 | | 0.119 | | 0.118 | | 0.118 | |
| Baseline | 0.116 | | 0.116 | | 0.121 | | 0.118 | | 0.120 | |
| 1-year | 0.120 | | 0.120 | | 0.119 | | 0.117 | | 0.118 | |
| 2-year | 0.125 | | 0.124 | | 0.124 | | 0.122 | | 0.122 | |
| 5-year | 0.131 | | 0.129 | | 0.129 | | 0.127 | | 0.127 | |
| RAE | | | | | | | | | | |
| Training data (80%) | 0.840 | | 0.837 | | 0.837 | | 0.821 | | 0.822 | |
| Validating data (20%) | 0.842 | | 0.840 | | 0.840 | | 0.828 | | 0.828 | |
| Baseline | 0.861 | | 0.863 | | 0.890 | | 0.881 | | 0.891 | |
| 1-year | 0.840 | | 0.838 | | 0.838 | | 0.823 | | 0.823 | |
| 2-year | 0.819 | | 0.816 | | 0.817 | | 0.800 | | 0.801 | |
| 5-year | 0.796 | | 0.790 | | 0.790 | | 0.774 | | 0.774 | |
| RRSE | | | | | | | | | | |
| Training data (80%) | 0.869 | | 0.866 | | 0.866 | | 0.852 | | 0.852 | |
| Validating data (20%) | 0.866 | | 0.863 | | 0.863 | | 0.850 | | 0.850 | |
| Baseline | 0.893 | | 0.896 | | 0.915 | | 0.913 | | 0.921 | |
| 1-year | 0.868 | | 0.866 | | 0.865 | | 0.852 | | 0.852 | |
| 2-year | 0.856 | | 0.854 | | 0.854 | | 0.837 | | 0.838 | |
| 5-year | 0.844 | | 0.839 | | 0.839 | | 0.825 | | 0.825 | |

**Mapping algorithm based on 2-year follow-up data**

| | Model 1 | | Model 2 | | Model 3 | | Model 4 | | Model 5 | |
|---|---|---|---|---|---|---|---|---|---|---|
| | coefficient | p-Value | coefficient | p-Value | coefficient | p-Value | coefficient | p-Value | coefficient | p-Value |
| **Intercept** | -0.080 | 0.000 | -0.035 | 0.070 | -0.025 | 0.214 | 0.105 | 0.000 | 0.111 | 0.000 |
| **OP summary score (transformed)** | 1.794 | 0.000 | 1.810 | 0.000 | 1.801 | 0.000 | 1.690 | 0.000 | 1.685 | 0.000 |
| **Log scale, Tobit model** | | | | | | | | | | |
| **phi (beta regression)** | - | - | - | - | - | - | - | - | - | - |
| *phi_Intercept* | 2.846 | 0.000 | 2.862 | 0.000 | 2.856 | 0.000 | 2.909 | 0.000 | 2.911 | 0.000 |
| *phi_OPS_transform* | -1.140 | 0.000 | -1.156 | 0.000 | -1.151 | 0.000 | -1.180 | 0.000 | -1.182 | 0.000 |
| *Patient characteristics* | | | | | | | | | | |
| *Age* [a] | | | | | | | | | | |
| 36-45 yrs | - | - | -0.041 | 0.056 | -0.039 | 0.076 | -0.003 | 0.880 | -0.004 | 0.865 |
| 46-55 yrs | - | - | -0.110 | 0.000 | -0.106 | 0.000 | -0.038 | 0.093 | -0.039 | 0.082 |
| 56-65 yrs | - | - | -0.150 | 0.000 | -0.149 | 0.000 | -0.076 | 0.006 | -0.077 | 0.006 |
| 65+ | - | - | -0.118 | 0.047 | -0.126 | 0.035 | -0.064 | 0.293 | -0.065 | 0.287 |
| Man [b] | - | - | 0.062 | 0.002 | 0.070 | 0.001 | 0.049 | 0.019 | 0.054 | 0.010 |
| *BMI* [c] | | | | | | | | | | |
| 40-44 | - | - | - | - | -0.158 | 0.001 | - | - | -0.104 | 0.023 |
| 45-49 | - | - | - | - | -0.032 | 0.686 | - | - | 0.063 | 0.430 |
| 50+ | - | - | - | - | -0.472 | 0.002 | - | - | -0.519 | 0.001 |
| *Comobidity* [d] | | | | | | | | | | |
| Sleep apnea | - | - | - | - | - | - | -0.131 | 0.002 | -0.126 | 0.003 |
| Hypertension | - | - | - | - | - | - | -0.066 | 0.004 | -0.067 | 0.004 |
| Diabetes | - | - | - | - | - | - | -0.085 | 0.012 | -0.089 | 0.009 |
| Dyslipidemia | - | - | - | - | - | - | 0.007 | 0.837 | 0.010 | 0.780 |
| Depression | - | - | - | - | - | - | -0.407 | 0.000 | -0.405 | 0.000 |
| **Goodness of fit** | | | | | | | | | | |
| Adj-Rsq, or pseudo R2 | 0.031 | | 0.032 | | 0.035 | | 0.035 | | 0.030 | |
| AIC | -20957 | | -20998 | | -20574 | | -21077 | | -21019 | |
| BIC | -20927 | | -20930 | | -20484 | | -20972 | | -20891 | |
| **Validation** | | | | | | | | | | |
| *MAE* | | | | | | | | | | |
| Training data (80%) | 0.100 | | 0.100 | | 0.100 | | 0.098 | | 0.098 | |
| Validating data (20%) | 0.100 | | 0.100 | | 0.099 | | 0.098 | | 0.098 | |
| Baseline | 0.090 | | 0.090 | | 0.098 | | 0.092 | | 0.098 | |
| 1-year | 0.097 | | 0.097 | | 0.097 | | 0.095 | | 0.095 | |
| 2-year | 0.100 | | 0.100 | | 0.100 | | 0.098 | | 0.098 | |
| 5-year | 0.103 | | 0.102 | | 0.102 | | 0.100 | | 0.099 | |
| RMSE | | | | | | | | | | |
| Training data (80%) | 0.124 | | 0.124 | | 0.124 | | 0.121 | | 0.121 | |
| Validating data (20%) | 0.124 | | 0.124 | | 0.123 | | 0.121 | | 0.121 | |
| Baseline | 0.114 | | 0.114 | | 0.124 | | 0.116 | | 0.124 | |
| 1-year | 0.120 | | 0.120 | | 0.120 | | 0.118 | | 0.118 | |
| 2-year | 0.124 | | 0.124 | | 0.124 | | 0.121 | | 0.121 | |
| 5-year | 0.129 | | 0.128 | | 0.128 | | 0.125 | | 0.125 | |
| RAE | | | | | | | | | | |
| Training data (80%) | 0.825 | | 0.822 | | 0.824 | | 0.805 | | 0.806 | |
| Validating data (20%) | 0.812 | | 0.810 | | 0.806 | | 0.790 | | 0.790 | |
| Baseline | 0.849 | | 0.848 | | 0.906 | | 0.868 | | 0.919 | |
| 1-year | 0.844 | | 0.841 | | 0.842 | | 0.826 | | 0.827 | |
| 2-year | 0.822 | | 0.820 | | 0.820 | | 0.802 | | 0.803 | |
| 5-year | 0.796 | | 0.790 | | 0.791 | | 0.774 | | 0.773 | |
| RRSE | | | | | | | | | | |
| Training data (80%) | 0.857 | | 0.855 | | 0.855 | | 0.838 | | 0.838 | |
| Validating data (20%) | 0.844 | | 0.842 | | 0.839 | | 0.823 | | 0.822 | |
| Baseline | 0.877 | | 0.878 | | 0.922 | | 0.897 | | 0.942 | |
| 1-year | 0.869 | | 0.866 | | 0.866 | | 0.852 | | 0.852 | |
| 2-year | 0.854 | | 0.852 | | 0.852 | | 0.835 | | 0.835 | |
| 5-year | 0.839 | | 0.835 | | 0.836 | | 0.820 | | 0.820 | |

**Mapping algorithm based on 5-year follow-up data**

| | Model 1 | | Model 2 | | Model 3 | | Model 4 | | Model 5 | |
|---|---|---|---|---|---|---|---|---|---|---|
| | coefficient | p-Value | coefficient | p-Value | coefficient | p-Value | coefficient | p-Value | coefficient | p-Value |
| **Intercept** | -0.101 | 0.000 | -0.004 | 0.887 | 0.008 | 0.758 | 0.142 | 0.000 | 0.144 | 0.000 |
| **OP summary score (transformed)** | 1.587 | 0.000 | 1.599 | 0.000 | 1.600 | 0.000 | 1.480 | 0.000 | 1.480 | 0.000 |
| **Log scale, Tobit model** | | | | | | | | | | |
| **phi (beta regression)** | - | - | - | - | - | - | - | - | - | - |
| *phi_Intercept* | 3.216 | 0.000 | 3.250 | 0.000 | 3.266 | 0.000 | 3.284 | 0.000 | 3.294 | 0.000 |
| *phi_OPS_transform* | -1.520 | 0.000 | -1.555 | 0.000 | -1.572 | 0.000 | -1.563 | 0.000 | -1.567 | 0.000 |
| *Patient characteristics* | | | | | | | | | | |
| *Age* [a] | | | | | | | | | | |
| 36-45 yrs | - | - | -0.113 | 0.000 | -0.129 | 0.000 | -0.118 | 0.000 | -0.118 | 0.000 |
| 46-55 yrs | - | - | -0.124 | 0.000 | -0.133 | 0.000 | -0.106 | 0.000 | -0.106 | 0.000 |
| 56-65 yrs | - | - | -0.196 | 0.000 | -0.203 | 0.000 | -0.157 | 0.000 | -0.159 | 0.000 |
| 65+ | - | - | -0.173 | 0.001 | -0.180 | 0.001 | -0.141 | 0.011 | -0.142 | 0.011 |
| Man [b] | - | - | 0.062 | 0.016 | 0.071 | 0.007 | 0.060 | 0.026 | 0.064 | 0.018 |
| *BMI* [c] | | | | | | | | | | |
| 40-44 | - | - | - | - | -0.075 | 0.076 | - | - | -0.047 | 0.268 |
| 45-49 | - | - | - | - | -0.029 | 0.702 | - | - | 0.066 | 0.397 |
| 50+ | - | - | - | - | 0.105 | 0.568 | - | - | 0.156 | 0.408 |
| *Comobidity* [d] | | | | | | | | | | |
| Sleep apnea | - | - | - | - | - | - | 0.005 | 0.928 | -0.005 | 0.924 |
| Hypertension | - | - | - | - | - | - | -0.038 | 0.151 | -0.035 | 0.178 |
| Diabetes | - | - | - | - | - | - | -0.076 | 0.056 | -0.070 | 0.082 |
| Dyslipidemia | - | - | - | - | - | - | 0.029 | 0.473 | 0.027 | 0.505 |
| Depression | - | - | - | - | - | - | -0.331 | 0.000 | -0.331 | 0.000 |
| **Goodness of fit** | | | | | | | | | | |
| Adj-Rsq, or pseudo R2 | 0.028 | | 0.028 | | 0.032 | | 0.032 | | | |
| AIC | -8419 | | -8453 | | -8156 | | -8408 | | -8358 | |
| BIC | -8392 | | -8392 | | -8076 | | -8315 | | -8245 | |
| **Validation** | | | | | | | | | | |
| *MAE* | | | | | | | | | | |
| Training data (80%) | 0.104 | | 0.103 | | 0.103 | | 0.101 | | 0.100 | |
| Validating data (20%) | 0.104 | | 0.103 | | 0.103 | | 0.102 | | 0.102 | |
| Baseline | 0.094 | | 0.092 | | 0.094 | | 0.091 | | 0.092 | |
| 1-year | 0.103 | | 0.102 | | 0.102 | | 0.101 | | 0.101 | |
| 2-year | 0.105 | | 0.104 | | 0.104 | | 0.102 | | 0.102 | |
| 5-year | 0.104 | | 0.103 | | 0.103 | | 0.101 | | 0.101 | |
| RMSE | | | | | | | | | | |
| Training data (80%) | 0.127 | | 0.126 | | 0.126 | | 0.123 | | 0.123 | |
| Validating data (20%) | 0.127 | | 0.126 | | 0.126 | | 0.124 | | 0.124 | |
| Baseline | 0.118 | | 0.116 | | 0.119 | | 0.115 | | 0.116 | |
| 1-year | 0.124 | | 0.123 | | 0.122 | | 0.121 | | 0.121 | |
| 2-year | 0.126 | | 0.125 | | 0.125 | | 0.123 | | 0.123 | |
| 5-year | 0.127 | | 0.126 | | 0.126 | | 0.123 | | 0.123 | |
| RAE | | | | | | | | | | |
| Training data (80%) | 0.797 | | 0.793 | | 0.794 | | 0.775 | | 0.774 | |
| Validating data (20%) | 0.795 | | 0.788 | | 0.787 | | 0.776 | | 0.775 | |
| Baseline | 0.866 | | 0.858 | | 0.874 | | 0.861 | | 0.870 | |
| 1-year | 0.851 | | 0.849 | | 0.848 | | 0.836 | | 0.836 | |
| 2-year | 0.829 | | 0.827 | | 0.828 | | 0.812 | | 0.813 | |
| 5-year | 0.796 | | 0.792 | | 0.792 | | 0.775 | | 0.774 | |
| RRSE | | | | | | | | | | |
| Training data (80%) | 0.833 | | 0.829 | | 0.829 | | 0.811 | | 0.810 | |
| Validating data (20%) | 0.836 | | 0.830 | | 0.831 | | 0.819 | | 0.818 | |
| Baseline | 0.878 | | 0.876 | | 0.888 | | 0.880 | | 0.889 | |
| 1-year | 0.873 | | 0.870 | | 0.870 | | 0.857 | | 0.857 | |
| 2-year | 0.856 | | 0.853 | | 0.854 | | 0.837 | | 0.838 | |
| 5-year | 0.833 | | 0.829 | | 0.830 | | 0.812 | | 0.812 | |

**Reference group**
**a 18-34 years**
**b Women**
**c <=39**
**d No disease**

## Table S5B. Mapping algorithm based on OLS models, Type B,  transfored SF-6D index applied

| | Mapping algorithm based on baseline data | | | | | | | | | | Mapping algorithm based on 1-year follow-up data | | | | | | | | | |
|---|---|---|---|---|---|---|---|---|---|---|---|---|---|---|---|---|---|---|---|---|
| | Model 1 | | Model 2 | | Model 3 | | Model 4 | | Model 5 | | Model 1 | | Model 2 | | Model 3 | | Model 4 | | Model 5 | |
| | coefficient | p-Value | coefficient | p-Value | coefficient | p-Value | coefficient | p-Value | coefficient | p-Value | coefficient | p-Value | coefficient | p-Value | coefficient | p-Value | coefficient | p-Value | coefficient | p-Value |
| **Intercept** | 0.783 | 0.000 | 0.788 | 0.000 | 0.795 | 0.000 | 0.790 | 0.000 | 0.797 | 0.000 | 0.854 | 0.000 | 0.861 | 0.000 | 0.862 | 0.000 | 0.868 | 0.000 | 0.868 | 0.000 |
| **OP dimensions** [a] | | | | | | | | | | | | | | | | | | | | |
| *Private gatherings in my own home (OP1)* | | | | | | | | | | | | | | | | | | | | |
| Limited difficulties | -0.023 | 0.000 | -0.023 | 0.000 | -0.024 | 0.000 | -0.023 | 0.000 | -0.024 | 0.000 | -0.022 | 0.000 | -0.023 | 0.000 | -0.023 | 0.000 | -0.021 | 0.000 | -0.021 | 0.000 |
| Some difficulties | -0.041 | 0.000 | -0.041 | 0.000 | -0.043 | 0.000 | -0.041 | 0.000 | -0.043 | 0.000 | -0.036 | 0.000 | -0.036 | 0.000 | -0.037 | 0.000 | -0.035 | 0.000 | -0.035 | 0.000 |
| Significant difficulties | -0.080 | 0.000 | -0.079 | 0.000 | -0.082 | 0.000 | -0.078 | 0.000 | -0.081 | 0.000 | -0.080 | 0.000 | -0.079 | 0.000 | -0.079 | 0.000 | -0.074 | 0.000 | -0.074 | 0.000 |
| *Private gatherings in a friend's or relative's home (OP2)* | | | | | | | | | | | | | | | | | | | | |
| Limited difficulties | -0.001 | 0.738 | -0.001 | 0.847 | 0.000 | 0.906 | 0.000 | 0.965 | 0.000 | 0.976 | -0.017 | 0.000 | -0.017 | 0.000 | -0.016 | 0.000 | -0.015 | 0.000 | -0.015 | 0.000 |
| Some difficulties | -0.001 | 0.691 | -0.001 | 0.728 | -0.002 | 0.665 | -0.001 | 0.851 | -0.001 | 0.788 | -0.024 | 0.000 | -0.024 | 0.000 | -0.023 | 0.000 | -0.018 | 0.000 | -0.018 | 0.000 |
| Significant difficulties | -0.014 | 0.000 | -0.014 | 0.000 | -0.015 | 0.000 | -0.013 | 0.001 | -0.014 | 0.000 | -0.036 | 0.000 | -0.036 | 0.000 | -0.036 | 0.000 | -0.027 | 0.001 | -0.027 | 0.001 |
| *Going to a restaurant (OP3)* | | | | | | | | | | | | | | | | | | | | |
| Limited difficulties | -0.008 | 0.004 | -0.008 | 0.002 | -0.008 | 0.006 | -0.009 | 0.002 | -0.008 | 0.004 | -0.001 | 0.772 | -0.001 | 0.688 | -0.001 | 0.715 | -0.004 | 0.203 | -0.004 | 0.204 |
| Some difficulties | -0.006 | 0.056 | -0.007 | 0.022 | -0.005 | 0.083 | -0.007 | 0.017 | -0.005 | 0.061 | -0.008 | 0.013 | -0.009 | 0.007 | -0.009 | 0.009 | -0.012 | 0.000 | -0.012 | 0.000 |
| Significant difficulties | -0.013 | 0.000 | -0.014 | 0.000 | -0.012 | 0.001 | -0.014 | 0.000 | -0.011 | 0.001 | -0.028 | 0.000 | -0.028 | 0.000 | -0.029 | 0.000 | -0.035 | 0.000 | -0.035 | 0.000 |
| *Going to community activities, courses etc (OP4)* | | | | | | | | | | | | | | | | | | | | |
| Limited difficulties | -0.021 | 0.000 | -0.021 | 0.000 | -0.022 | 0.000 | -0.021 | 0.000 | -0.021 | 0.000 | -0.035 | 0.000 | -0.035 | 0.000 | -0.035 | 0.000 | -0.033 | 0.000 | -0.033 | 0.000 |
| Some difficulties | -0.045 | 0.000 | -0.044 | 0.000 | -0.044 | 0.000 | -0.043 | 0.000 | -0.043 | 0.000 | -0.077 | 0.000 | -0.076 | 0.000 | -0.076 | 0.000 | -0.071 | 0.000 | -0.071 | 0.000 |
| Significant difficulties | -0.076 | 0.000 | -0.076 | 0.000 | -0.075 | 0.000 | -0.074 | 0.000 | -0.073 | 0.000 | -0.100 | 0.000 | -0.098 | 0.000 | -0.098 | 0.000 | -0.090 | 0.000 | -0.091 | 0.000 |
| *Vacations away from home (OP5)* | | | | | | | | | | | | | | | | | | | | |
| Limited difficulties | -0.013 | 0.000 | -0.014 | 0.000 | -0.013 | 0.000 | -0.013 | 0.000 | -0.013 | 0.000 | -0.014 | 0.000 | -0.015 | 0.000 | -0.015 | 0.000 | -0.013 | 0.000 | -0.013 | 0.000 |
| Some difficulties | -0.018 | 0.000 | -0.018 | 0.000 | -0.017 | 0.000 | -0.018 | 0.000 | -0.017 | 0.000 | -0.016 | 0.000 | -0.018 | 0.000 | -0.017 | 0.000 | -0.018 | 0.000 | -0.018 | 0.000 |
| Significant difficulties | -0.029 | 0.000 | -0.030 | 0.000 | -0.029 | 0.000 | -0.030 | 0.000 | -0.029 | 0.000 | -0.015 | 0.021 | -0.018 | 0.006 | -0.017 | 0.008 | -0.020 | 0.001 | -0.020 | 0.002 |
| *Trying on and buying clothes (OP6)* | | | | | | | | | | | | | | | | | | | | |
| Limited difficulties | -0.008 | 0.071 | -0.008 | 0.071 | -0.008 | 0.080 | -0.008 | 0.069 | -0.008 | 0.076 | -0.017 | 0.000 | -0.018 | 0.000 | -0.018 | 0.000 | -0.016 | 0.000 | -0.016 | 0.000 |
| Some difficulties | 0.000 | 0.988 | 0.000 | 0.930 | -0.001 | 0.876 | 0.000 | 0.991 | 0.000 | 0.947 | -0.027 | 0.000 | -0.027 | 0.000 | -0.026 | 0.000 | -0.024 | 0.000 | -0.023 | 0.000 |
| Significant difficulties | -0.004 | 0.327 | -0.005 | 0.223 | -0.005 | 0.179 | -0.005 | 0.250 | -0.005 | 0.199 | -0.029 | 0.000 | -0.029 | 0.000 | -0.028 | 0.000 | -0.025 | 0.000 | -0.024 | 0.000 |
| *Bathing in public places (beach, public pool, etc) (OP7)* | | | | | | | | | | | | | | | | | | | | |
| Limited difficulties | 0.011 | 0.004 | 0.011 | 0.005 | 0.011 | 0.004 | 0.011 | 0.005 | 0.011 | 0.004 | -0.003 | 0.230 | -0.004 | 0.172 | -0.004 | 0.126 | -0.004 | 0.083 | -0.004 | 0.089 |
| Some difficulties | 0.019 | 0.000 | 0.018 | 0.000 | 0.018 | 0.000 | 0.017 | 0.000 | 0.018 | 0.000 | -0.004 | 0.143 | -0.004 | 0.077 | -0.005 | 0.057 | -0.006 | 0.020 | -0.006 | 0.022 |
| Significant difficulties | 0.017 | 0.000 | 0.015 | 0.000 | 0.016 | 0.000 | 0.014 | 0.000 | 0.015 | 0.000 | -0.009 | 0.007 | -0.010 | 0.003 | -0.010 | 0.002 | -0.012 | 0.000 | -0.012 | 0.000 |
| *Intimate relations (OP8)* | | | | | | | | | | | | | | | | | | | | |
| Limited difficulties | -0.011 | 0.000 | -0.011 | 0.000 | -0.011 | 0.000 | -0.010 | 0.000 | -0.011 | 0.000 | -0.020 | 0.000 | -0.019 | 0.000 | -0.019 | 0.000 | -0.018 | 0.000 | -0.018 | 0.000 |
| Some difficulties | -0.025 | 0.000 | -0.025 | 0.000 | -0.026 | 0.000 | -0.024 | 0.000 | -0.025 | 0.000 | -0.034 | 0.000 | -0.034 | 0.000 | -0.034 | 0.000 | -0.032 | 0.000 | -0.032 | 0.000 |
| Significant difficulties | -0.049 | 0.000 | -0.048 | 0.000 | -0.048 | 0.000 | -0.045 | 0.000 | -0.046 | 0.000 | -0.042 | 0.000 | -0.041 | 0.000 | -0.042 | 0.000 | -0.038 | 0.000 | -0.039 | 0.000 |
| **Patient characteristics** | | | | | | | | | | | | | | | | | | | | |
| *Age* [a] | | | | | | | | | | | | | | | | | | | | |
| 36-45 yrs | - | - | 0.001 | 0.724 | -0.001 | 0.601 | 0.004 | 0.028 | 0.002 | 0.198 | - | - | -0.008 | 0.000 | -0.008 | 0.000 | -0.004 | 0.042 | -0.004 | 0.039 |
| 46-55 yrs | - | - | -0.007 | 0.000 | -0.009 | 0.000 | -0.001 | 0.427 | -0.004 | 0.044 | - | - | -0.015 | 0.000 | -0.015 | 0.000 | -0.009 | 0.000 | -0.009 | 0.000 |
| 56-65 yrs | - | - | -0.013 | 0.000 | -0.015 | 0.000 | -0.005 | 0.054 | -0.007 | 0.004 | - | - | -0.023 | 0.000 | -0.024 | 0.000 | -0.015 | 0.000 | -0.015 | 0.000 |
| 65+ | - | - | -0.012 | 0.139 | -0.015 | 0.077 | -0.004 | 0.658 | -0.006 | 0.440 | - | - | -0.016 | 0.046 | -0.016 | 0.054 | -0.007 | 0.365 | -0.008 | 0.342 |
| Man [b] | - | - | -0.001 | 0.411 | 0.001 | 0.548 | 0.000 | 0.807 | 0.002 | 0.183 | - | - | 0.013 | 0.000 | 0.013 | 0.000 | 0.010 | 0.000 | 0.011 | 0.000 |
| *BMI* [d] | | | | | | | | | | | | | | | | | | | | |
| 40-44 | - | - | - | - | -0.008 | 0.000 | - | - | -0.007 | 0.000 | - | - | - | - | -0.015 | 0.018 | - | - | -0.009 | 0.153 |
| 45-49 | - | - | - | - | -0.013 | 0.000 | - | - | -0.013 | 0.000 | - | - | - | - | -0.036 | 0.016 | - | - | -0.026 | 0.071 |
| 50+ | - | - | - | - | -0.024 | 0.000 | - | - | -0.022 | 0.000 | - | - | - | - | 0.011 | 0.620 | - | - | 0.013 | 0.577 |
| *Comobidity e* | | | | | | | | | | | | | | | | | | | | |
| Sleep apnea | - | - | - | - | - | - | -0.015 | 0.000 | -0.014 | 0.000 | - | - | - | - | - | - | -0.020 | 0.000 | -0.019 | 0.000 |
| Hypertension | - | - | - | - | - | - | -0.002 | 0.235 | -0.001 | 0.503 | - | - | - | - | - | - | -0.009 | 0.000 | -0.009 | 0.000 |
| Diabetes | - | - | - | - | - | - | -0.007 | 0.001 | -0.007 | 0.001 | - | - | - | - | - | - | -0.011 | 0.007 | -0.011 | 0.008 |
| Dyslipidemia | - | - | - | - | - | - | -0.003 | 0.271 | -0.004 | 0.142 | - | - | - | - | - | - | 0.000 | 0.998 | 0.000 | 0.998 |
| Depression | - | - | - | - | - | - | -0.029 | 0.000 | -0.029 | 0.000 | - | - | - | - | - | - | -0.070 | 0.000 | -0.069 | 0.000 |
| **Goodness of fit** | | | | | | | | | | | | | | | | | | | | |
| Adj-Rsq, or pseudo R2 | 0.318 | | 0.319 | | 0.322 | | 0.328 | | 0.330 | | 0.277 | | 0.281 | | 0.282 | | 0.313 | | 0.313 | |
| AIC | -48353 | | -48394 | | -48507 | | -48774 | | -48872 | | -31475 | | -31589 | | -31379 | | -32342 | | -32322 | |
| BIC | -48138 | | -48137 | | -48225 | | -48475 | | -48549 | | -31267 | | -31342 | | -31108 | | -32055 | | -32010 | |
| **Validation** | | | | | | | | | | | | | | | | | | | | |
| *MAE* | | | | | | | | | | | | | | | | | | | | |
| Training data (80%) | 0.085 | | 0.085 | | 0.085 | | 0.085 | | 0.085 | | 0.092 | | 0.092 | | 0.092 | | 0.089 | | 0.089 | |
| Validating data (20%) | 0.084 | | 0.083 | | 0.083 | | 0.083 | | 0.082 | | 0.093 | | 0.092 | | 0.092 | | 0.090 | | 0.090 | |
| Baseline | 0.085 | | 0.085 | | 0.085 | | 0.084 | | 0.084 | | 0.086 | | 0.086 | | 0.087 | | 0.086 | | 0.087 | |
| 1-year | 0.111 | | 0.110 | | 0.108 | | 0.108 | | 0.106 | | 0.092 | | 0.092 | | 0.092 | | 0.090 | | 0.090 | |
| 2-year | 0.111 | | 0.111 | | 0.109 | | 0.108 | | 0.107 | | 0.095 | | 0.095 | | 0.095 | | 0.092 | | 0.092 | |
| 5-year | 0.109 | | 0.109 | | 0.108 | | 0.106 | | 0.105 | | 0.099 | | 0.098 | | 0.098 | | 0.095 | | 0.095 | |
| RMSE | | | | | | | | | | | | | | | | | | | | |
| Training data (80%) | 0.106 | | 0.106 | | 0.106 | | 0.106 | | 0.105 | | 0.117 | | 0.117 | | 0.117 | | 0.114 | | 0.114 | |
| Validating data (20%) | 0.104 | | 0.104 | | 0.104 | | 0.103 | | 0.103 | | 0.117 | | 0.117 | | 0.117 | | 0.114 | | 0.114 | |
| Baseline | 0.106 | | 0.106 | | 0.106 | | 0.105 | | 0.105 | | 0.110 | | 0.110 | | 0.111 | | 0.109 | | 0.110 | |
| 1-year | 0.129 | | 0.129 | | 0.127 | | 0.127 | | 0.125 | | 0.117 | | 0.117 | | 0.117 | | 0.114 | | 0.114 | |
| 2-year | 0.130 | | 0.130 | | 0.128 | | 0.127 | | 0.126 | | 0.122 | | 0.121 | | 0.121 | | 0.118 | | 0.118 | |
| 5-year | 0.129 | | 0.129 | | 0.128 | | 0.127 | | 0.126 | | 0.127 | | 0.126 | | 0.126 | | 0.123 | | 0.122 | |
| RAE | | | | | | | | | | | | | | | | | | | | |
| Training data (80%) | 0.805 | | 0.804 | | 0.802 | | 0.798 | | 0.796 | | 0.812 | | 0.810 | | 0.809 | | 0.789 | | 0.789 | |
| Validating data (20%) | 0.792 | | 0.789 | | 0.788 | | 0.782 | | 0.781 | | 0.810 | | 0.807 | | 0.807 | | 0.789 | | 0.789 | |
| Baseline | 0.802 | | 0.801 | | 0.799 | | 0.795 | | 0.793 | | 0.815 | | 0.815 | | 0.826 | | 0.815 | | 0.820 | |
| 1-year | 0.878 | | 0.876 | | 0.872 | | 0.862 | | 0.858 | | 0.811 | | 0.809 | | 0.809 | | 0.789 | | 0.789 | |
| 2-year | 0.858 | | 0.858 | | 0.855 | | 0.843 | | 0.839 | | 0.790 | | 0.787 | | 0.788 | | 0.764 | | 0.764 | |
| 5-year | 0.827 | | 0.827 | | 0.825 | | 0.809 | | 0.806 | | 0.765 | | 0.760 | | 0.761 | | 0.738 | | 0.737 | |
| RRSE | | | | | | | | | | | | | | | | | | | | |
| Training data (80%) | 0.826 | | 0.825 | | 0.823 | | 0.819 | | 0.818 | | 0.850 | | 0.848 | | 0.847 | | 0.828 | | 0.828 | |
| Validating data (20%) | 0.815 | | 0.813 | | 0.812 | | 0.808 | | 0.806 | | 0.844 | | 0.842 | | 0.842 | | 0.824 | | 0.825 | |
| Baseline | 0.824 | | 0.823 | | 0.821 | | 0.817 | | 0.816 | | 0.849 | | 0.850 | | 0.856 | | 0.848 | | 0.851 | |
| 1-year | 0.890 | | 0.889 | | 0.885 | | 0.876 | | 0.872 | | 0.849 | | 0.846 | | 0.846 | | 0.828 | | 0.828 | |
| 2-year | 0.872 | | 0.871 | | 0.869 | | 0.857 | | 0.854 | | 0.835 | | 0.833 | | 0.833 | | 0.811 | | 0.811 | |
| 5-year | 0.848 | | 0.847 | | 0.846 | | 0.832 | | 0.830 | | 0.823 | | 0.819 | | 0.819 | | 0.797 | | 0.796 | |

| | Mapping algorithm based on 2-year follow-up data | | | | | | | | | | Mapping algorithm based on 5-year follow-up data | | | | | | | | | |
|---|---|---|---|---|---|---|---|---|---|---|---|---|---|---|---|---|---|---|---|---|
| | Model 1 | | Model 2 | | Model 3 | | Model 4 | | Model 5 | | Model 1 | | Model 2 | | Model 3 | | Model 4 | | Model 5 | |
| | coefficient | p-Value | coefficient | p-Value | coefficient | p-Value | coefficient | p-Value | coefficient | p-Value | coefficient | p-Value | coefficient | p-Value | coefficient | p-Value | coefficient | p-Value | coefficient | p-Value |
| **Intercept** | 0.846 | 0.000 | 0.854 | 0.000 | 0.854 | 0.000 | 0.860 | 0.000 | 0.860 | 0.000 | 0.827 | 0.000 | 0.840 | 0.000 | 0.840 | 0.000 | 0.848 | 0.000 | 0.848 | 0.000 |
| **OP dimensions** [a] | | | | | | | | | | | | | | | | | | | | |
| *Private gatherings in my own home (OP1)* | | | | | | | | | | | | | | | | | | | | |
| Limited difficulties | -0.022 | 0.000 | -0.023 | 0.000 | -0.023 | 0.000 | -0.022 | 0.000 | -0.022 | 0.000 | -0.015 | 0.023 | -0.015 | 0.022 | -0.017 | 0.015 | -0.012 | 0.071 | -0.014 | 0.030 |
| Some difficulties | -0.038 | 0.000 | -0.038 | 0.000 | -0.039 | 0.000 | -0.036 | 0.000 | -0.037 | 0.000 | -0.032 | 0.000 | -0.030 | 0.001 | -0.034 | 0.000 | -0.029 | 0.001 | -0.031 | 0.001 |
| Significant difficulties | -0.076 | 0.000 | -0.075 | 0.000 | -0.075 | 0.000 | -0.071 | 0.000 | -0.072 | 0.000 | -0.082 | 0.000 | -0.081 | 0.000 | -0.086 | 0.000 | -0.076 | 0.000 | -0.079 | 0.000 |
| *Private gatherings in a friend's or relative's home (OP2)* | | | | | | | | | | | | | | | | | | | | |
| Limited difficulties | -0.021 | 0.000 | -0.022 | 0.000 | -0.022 | 0.000 | -0.021 | 0.000 | -0.021 | 0.000 | -0.017 | 0.014 | -0.019 | 0.007 | -0.018 | 0.014 | -0.018 | 0.011 | -0.016 | 0.019 |
| Some difficulties | -0.030 | 0.000 | -0.031 | 0.000 | -0.032 | 0.000 | -0.025 | 0.000 | -0.026 | 0.000 | -0.025 | 0.007 | -0.026 | 0.004 | -0.025 | 0.008 | -0.021 | 0.023 | -0.019 | 0.033 |
| Significant difficulties | -0.036 | 0.000 | -0.037 | 0.000 | -0.037 | 0.000 | -0.026 | 0.008 | -0.026 | 0.008 | -0.041 | 0.004 | -0.040 | 0.004 | -0.038 | 0.009 | -0.030 | 0.030 | -0.027 | 0.056 |
| *Going to a restaurant (OP3)* | | | | | | | | | | | | | | | | | | | | |
| Limited difficulties | 0.003 | 0.411 | 0.003 | 0.458 | 0.002 | 0.545 | 0.001 | 0.774 | 0.001 | 0.765 | -0.001 | 0.864 | -0.001 | 0.830 | -0.001 | 0.897 | -0.002 | 0.709 | -0.002 | 0.805 |
| Some difficulties | 0.006 | 0.238 | 0.005 | 0.274 | 0.005 | 0.246 | 0.001 | 0.821 | 0.002 | 0.724 | 0.002 | 0.835 | 0.001 | 0.893 | 0.001 | 0.857 | -0.004 | 0.614 | -0.002 | 0.776 |
| Significant difficulties | -0.003 | 0.684 | -0.004 | 0.613 | -0.005 | 0.555 | -0.012 | 0.141 | -0.012 | 0.139 | -0.004 | 0.780 | -0.005 | 0.682 | -0.008 | 0.519 | -0.016 | 0.214 | -0.017 | 0.168 |
| *Going to community activities, courses etc (OP4)* | | | | | | | | | | | | | | | | | | | | |
| Limited difficulties | -0.039 | 0.000 | -0.038 | 0.000 | -0.038 | 0.000 | -0.035 | 0.000 | -0.035 | 0.000 | -0.028 | 0.000 | -0.028 | 0.000 | -0.029 | 0.000 | -0.028 | 0.000 | -0.027 | 0.000 |
| Some difficulties | -0.080 | 0.000 | -0.078 | 0.000 | -0.078 | 0.000 | -0.073 | 0.000 | -0.072 | 0.000 | -0.060 | 0.000 | -0.060 | 0.000 | -0.059 | 0.000 | -0.053 | 0.000 | -0.053 | 0.000 |
| Significant difficulties | -0.123 | 0.000 | -0.122 | 0.000 | -0.120 | 0.000 | -0.109 | 0.000 | -0.108 | 0.000 | -0.102 | 0.000 | -0.101 | 0.000 | -0.095 | 0.000 | -0.081 | 0.000 | -0.080 | 0.000 |
| *Vacations away from home (OP5)* | | | | | | | | | | | | | | | | | | | | |
| Limited difficulties | -0.012 | 0.006 | -0.012 | 0.004 | -0.011 | 0.009 | -0.011 | 0.010 | -0.010 | 0.014 | -0.015 | 0.020 | -0.014 | 0.032 | -0.013 | 0.052 | -0.012 | 0.064 | -0.013 | 0.049 |
| Some difficulties | -0.016 | 0.002 | -0.017 | 0.001 | -0.016 | 0.002 | -0.017 | 0.001 | -0.017 | 0.001 | -0.015 | 0.050 | -0.015 | 0.053 | -0.015 | 0.061 | -0.016 | 0.038 | -0.018 | 0.022 |
| Significant difficulties | -0.003 | 0.662 | -0.005 | 0.488 | -0.006 | 0.475 | -0.008 | 0.279 | -0.009 | 0.257 | -0.021 | 0.066 | -0.023 | 0.047 | -0.016 | 0.174 | -0.016 | 0.158 | -0.015 | 0.187 |
| *Trying on and buying clothes (OP6)* | | | | | | | | | | | | | | | | | | | | |
| Limited difficulties | -0.015 | 0.000 | -0.015 | 0.000 | -0.015 | 0.000 | -0.014 | 0.000 | -0.014 | 0.000 | -0.019 | 0.001 | -0.020 | 0.000 | -0.021 | 0.000 | -0.019 | 0.001 | -0.018 | 0.001 |
| Some difficulties | -0.026 | 0.000 | -0.026 | 0.000 | -0.025 | 0.000 | -0.025 | 0.000 | -0.025 | 0.000 | -0.028 | 0.000 | -0.028 | 0.000 | -0.028 | 0.000 | -0.027 | 0.000 | -0.026 | 0.000 |
| Significant difficulties | -0.030 | 0.000 | -0.030 | 0.000 | -0.028 | 0.000 | -0.029 | 0.000 | -0.026 | 0.000 | -0.036 | 0.000 | -0.036 | 0.000 | -0.039 | 0.000 | -0.036 | 0.000 | -0.037 | 0.000 |
| *Bathing in public places (beach, public pool, etc) (OP7)* | | | | | | | | | | | | | | | | | | | | |
| Limited difficulties | -0.009 | 0.010 | -0.009 | 0.011 | -0.009 | 0.011 | -0.010 | 0.005 | -0.010 | 0.005 | 0.001 | 0.816 | 0.002 | 0.733 | 0.004 | 0.512 | 0.003 | 0.657 | 0.003 | 0.593 |
| Some difficulties | -0.006 | 0.102 | -0.006 | 0.066 | -0.007 | 0.050 | -0.008 | 0.016 | -0.008 | 0.023 | 0.000 | 0.958 | -0.001 | 0.830 | -0.002 | 0.777 | -0.003 | 0.599 | -0.003 | 0.657 |
| Significant difficulties | -0.007 | 0.109 | -0.008 | 0.057 | -0.008 | 0.061 | -0.011 | 0.007 | -0.011 | 0.012 | -0.007 | 0.295 | -0.009 | 0.192 | -0.009 | 0.224 | -0.009 | 0.190 | -0.008 | 0.243 |
| *Intimate relations (OP8)* | | | | | | | | | | | | | | | | | | | | |
| Limited difficulties | -0.020 | 0.000 | -0.020 | 0.000 | -0.019 | 0.000 | -0.016 | 0.000 | -0.017 | 0.000 | -0.031 | 0.000 | -0.030 | 0.000 | -0.029 | 0.000 | -0.028 | 0.000 | -0.027 | 0.000 |
| Some difficulties | -0.034 | 0.000 | -0.033 | 0.000 | -0.033 | 0.000 | -0.029 | 0.000 | -0.029 | 0.000 | -0.043 | 0.000 | -0.042 | 0.000 | -0.042 | 0.000 | -0.039 | 0.000 | -0.039 | 0.000 |
| Significant difficulties | -0.049 | 0.000 | -0.047 | 0.000 | -0.046 | 0.000 | -0.042 | 0.000 | -0.043 | 0.000 | -0.059 | 0.000 | -0.056 | 0.000 | -0.057 | 0.000 | -0.054 | 0.000 | -0.054 | 0.000 |
| **Patient characteristics** | | | | | | | | | | | | | | | | | | | | |
| *Age* [a] | | | | | | | | | | | | | | | | | | | | |
| 36-45 yrs | - | - | -0.010 | 0.001 | -0.010 | 0.001 | -0.005 | 0.081 | -0.005 | 0.069 | - | - | -0.016 | 0.002 | -0.017 | 0.001 | -0.015 | 0.004 | -0.014 | 0.005 |
| 46-55 yrs | - | - | -0.014 | 0.000 | -0.014 | 0.000 | -0.006 | 0.037 | -0.006 | 0.034 | - | - | -0.020 | 0.000 | -0.019 | 0.000 | -0.014 | 0.004 | -0.014 | 0.007 |
| 56-65 yrs | - | - | -0.020 | 0.000 | -0.020 | 0.000 | -0.011 | 0.002 | -0.012 | 0.001 | - | - | -0.027 | 0.000 | -0.027 | 0.000 | -0.020 | 0.000 | -0.021 | 0.000 |
| 65+ | - | - | -0.015 | 0.054 | -0.017 | 0.035 | -0.008 | 0.329 | -0.008 | 0.301 | - | - | -0.024 | 0.005 | -0.025 | 0.003 | -0.020 | 0.021 | -0.021 | 0.015 |
| Man [b] | - | - | 0.015 | 0.000 | 0.015 | 0.000 | 0.012 | 0.000 | 0.012 | 0.000 | - | - | 0.022 | 0.000 | 0.023 | 0.000 | 0.018 | 0.000 | 0.019 | 0.000 |
| *BMI* [d] | | | | | | | | | | | | | | | | | | | | |
| 40-44 | - | - | - | - | -0.025 | 0.003 | - | - | -0.018 | 0.025 | - | - | - | - | -0.016 | 0.098 | - | - | -0.013 | 0.185 |
| 45-49 | - | - | - | - | -0.024 | 0.159 | - | - | -0.016 | 0.352 | - | - | - | - | -0.019 | 0.329 | - | - | -0.012 | 0.507 |
| 50+ | - | - | - | - | -0.113 | 0.001 | - | - | -0.116 | 0.000 | - | - | - | - | 0.041 | 0.296 | - | - | 0.052 | 0.180 |
| *Comobidity e* | | | | | | | | | | | | | | | | | | | | |
| Sleep apnea | - | - | - | - | - | - | -0.019 | 0.002 | -0.017 | 0.007 | - | - | - | - | - | - | 0.001 | 0.952 | 0.001 | 0.949 |
| Hypertension | - | - | - | - | - | - | -0.008 | 0.006 | -0.008 | 0.008 | - | - | - | - | - | - | -0.008 | 0.067 | -0.008 | 0.068 |
| Diabetes | - | - | - | - | - | - | -0.007 | 0.149 | -0.007 | 0.126 | - | - | - | - | - | - | -0.012 | 0.104 | -0.010 | 0.162 |
| Dyslipidemia | - | - | - | - | - | - | -0.005 | 0.316 | -0.005 | 0.340 | - | - | - | - | - | - | 0.000 | 0.953 | 0.001 | 0.841 |
| Depression | - | - | - | - | - | - | -0.076 | 0.000 | -0.076 | 0.000 | - | - | - | - | - | - | -0.084 | 0.000 | -0.084 | 0.000 |
| **Goodness of fit** | | | | | | | | | | | | | | | | | | | | |
| Adj-Rsq, or pseudo R2 | 0.301 | | 0.304 | | 0.303 | | 0.338 | | 0.338 | | 0.333 | | 0.338 | | 0.337 | | 0.378 | | 0.379 | |
| AIC | -19248 | | -19308 | | -18928 | | -19687 | | -19633 | | -7910 | | -7953 | | -7679 | | -8078 | | -8048 | |
| BIC | -19052 | | -19074 | | -18673 | | -19417 | | -19339 | | -7736 | | -7745 | | -7452 | | -7838 | | -7789 | |
| **Validation** | | | | | | | | | | | | | | | | | | | | |
| *MAE* | | | | | | | | | | | | | | | | | | | | |
| Training data (80%) | 0.096 | | 0.096 | | 0.096 | | 0.093 | | 0.093 | | 0.100 | | 0.099 | | 0.099 | | 0.096 | | 0.095 | |
| Validating data (20%) | 0.097 | | 0.097 | | 0.096 | | 0.093 | | 0.093 | | 0.100 | | 0.100 | | 0.100 | | 0.097 | | 0.097 | |
| Baseline | 0.087 | | 0.087 | | 0.091 | | 0.087 | | 0.091 | | 0.091 | | 0.090 | | 0.092 | | 0.089 | | 0.091 | |
| 1-year | 0.094 | | 0.094 | | 0.094 | | 0.091 | | 0.091 | | 0.100 | | 0.098 | | 0.098 | | 0.095 | | 0.095 | |
| 2-year | 0.097 | | 0.096 | | 0.096 | | 0.093 | | 0.093 | | 0.101 | | 0.100 | | 0.100 | | 0.097 | | 0.097 | |
| 5-year | 0.098 | | 0.098 | | 0.098 | | 0.095 | | 0.095 | | 0.100 | | 0.100 | | 0.099 | | 0.096 | | 0.096 | |
| RMSE | | | | | | | | | | | | | | | | | | | | |
| Training data (80%) | 0.121 | | 0.121 | | 0.121 | | 0.118 | | 0.118 | | 0.124 | | 0.124 | | 0.123 | | 0.120 | | 0.119 | |
| Validating data (20%) | 0.121 | | 0.121 | | 0.120 | | 0.117 | | 0.117 | | 0.124 | | 0.123 | | 0.124 | | 0.121 | | 0.121 | |
| Baseline | 0.110 | | 0.110 | | 0.116 | | 0.110 | | 0.115 | | 0.115 | | 0.114 | | 0.117 | | 0.112 | | 0.114 | |
| 1-year | 0.118 | | 0.117 | | 0.117 | | 0.115 | | 0.115 | | 0.121 | | 0.120 | | 0.120 | | 0.117 | | 0.117 | |
| 2-year | 0.121 | | 0.121 | | 0.121 | | 0.118 | | 0.117 | | 0.123 | | 0.122 | | 0.122 | | 0.119 | | 0.119 | |
| 5-year | 0.126 | | 0.125 | | 0.125 | | 0.121 | | 0.121 | | 0.124 | | 0.124 | | 0.123 | | 0.120 | | 0.120 | |
| RAE | | | | | | | | | | | | | | | | | | | | |
| Training data (80%) | 0.792 | | 0.789 | | 0.791 | | 0.766 | | 0.766 | | 0.768 | | 0.764 | | 0.764 | | 0.736 | | 0.735 | |
| Validating data (20%) | 0.784 | | 0.782 | | 0.778 | | 0.754 | | 0.754 | | 0.764 | | 0.762 | | 0.761 | | 0.742 | | 0.741 | |
| Baseline | 0.819 | | 0.820 | | 0.852 | | 0.820 | | 0.851 | | 0.842 | | 0.839 | | 0.856 | | 0.838 | | 0.852 | |
| 1-year | 0.814 | | 0.812 | | 0.812 | | 0.790 | | 0.791 | | 0.826 | | 0.822 | | 0.822 | | 0.800 | | 0.800 | |
| 2-year | 0.790 | | 0.788 | | 0.788 | | 0.763 | | 0.764 | | 0.803 | | 0.800 | | 0.800 | | 0.775 | | 0.776 | |
| 5-year | 0.763 | | 0.759 | | 0.761 | | 0.735 | | 0.735 | | 0.767 | | 0.763 | | 0.764 | | 0.737 | | 0.736 | |
| RRSE | | | | | | | | | | | | | | | | | | | | |
| Training data (80%) | 0.836 | | 0.834 | | 0.834 | | 0.812 | | 0.813 | | 0.815 | | 0.812 | | 0.812 | | 0.787 | | 0.786 | |
| Validating data (20%) | 0.826 | | 0.824 | | 0.820 | | 0.798 | | 0.797 | | 0.819 | | 0.815 | | 0.816 | | 0.800 | | 0.799 | |
| Baseline | 0.849 | | 0.850 | | 0.872 | | 0.849 | | 0.872 | | 0.857 | | 0.858 | | 0.871 | | 0.858 | | 0.871 | |
| 1-year | 0.851 | | 0.848 | | 0.848 | | 0.830 | | 0.830 | | 0.858 | | 0.855 | | 0.854 | | 0.837 | | 0.837 | |
| 2-year | 0.834 | | 0.832 | | 0.831 | | 0.809 | | 0.810 | | 0.839 | | 0.837 | | 0.837 | | 0.816 | | 0.816 | |
| 5-year | 0.820 | | 0.816 | | 0.818 | | 0.794 | | 0.794 | | 0.816 | | 0.812 | | 0.813 | | 0.789 | | 0.788 | |

**Reference group**
**a no difficulties**
**b 18-34 years**
**c Women**
**d <=39**
**e No disease**

# Table S6B. Mapping algorithm based on Tobit models, Type B,  transfored SF-6D index applied

| | Mapping algorithm based on baseline data | | | | | | | | | |
|---|---|---|---|---|---|---|---|---|---|---|
| | Model 1 | | Model 2 | | Model 3 | | Model 4 | | Model 5 | |
| | coefficient | p-Value | coefficient | p-Value | coefficient | p-Value | coefficient | p-Value | coefficient | p-Value |
| **Intercept** | 0.784 | 0.000 | 0.789 | 0.000 | 0.796 | 0.000 | 0.791 | 0.000 | 0.798 | 0.000 |
| **OP dimensions [a]** | | | | | | | | | | |
| *Private gatherings in my own hon* | | | | | | | | | | |
| Limited difficulties | -0.023 | 0.000 | -0.023 | 0.000 | -0.024 | 0.000 | -0.022 | 0.000 | -0.024 | 0.000 |
| Some difficulties | -0.041 | 0.000 | -0.041 | 0.000 | -0.043 | 0.000 | -0.041 | 0.000 | -0.043 | 0.000 |
| Significant difficulties | -0.080 | 0.000 | -0.079 | 0.000 | -0.082 | 0.000 | -0.078 | 0.000 | -0.081 | 0.000 |
| *Private gatherings in a friend's or* | | | | | | | | | | |
| Limited difficulties | -0.001 | 0.697 | -0.001 | 0.803 | -0.001 | 0.862 | 0.000 | 0.920 | 0.000 | 0.980 |
| Some difficulties | -0.002 | 0.659 | -0.001 | 0.695 | -0.002 | 0.634 | -0.001 | 0.817 | -0.001 | 0.754 |
| Significant difficulties | -0.014 | 0.000 | -0.014 | 0.000 | -0.015 | 0.000 | -0.013 | 0.001 | -0.014 | 0.000 |
| *Going to a restaurant (OP3)* | | | | | | | | | | |
| Limited difficulties | -0.008 | 0.004 | -0.008 | 0.003 | -0.007 | 0.006 | -0.009 | 0.002 | -0.008 | 0.004 |
| Some difficulties | -0.005 | 0.060 | -0.006 | 0.024 | -0.005 | 0.090 | -0.007 | 0.018 | -0.005 | 0.066 |
| Significant difficulties | -0.013 | 0.000 | -0.014 | 0.000 | -0.012 | 0.001 | -0.014 | 0.000 | -0.012 | 0.001 |
| *Going to community activities, co* | | | | | | | | | | |
| Limited difficulties | -0.021 | 0.000 | -0.021 | 0.000 | -0.022 | 0.000 | -0.021 | 0.000 | -0.021 | 0.000 |
| Some difficulties | -0.045 | 0.000 | -0.044 | 0.000 | -0.044 | 0.000 | -0.043 | 0.000 | -0.043 | 0.000 |
| Significant difficulties | -0.076 | 0.000 | -0.076 | 0.000 | -0.075 | 0.000 | -0.074 | 0.000 | -0.073 | 0.000 |
| *Vacations away from home (OP5,* | | | | | | | | | | |
| Limited difficulties | -0.014 | 0.000 | -0.014 | 0.000 | -0.013 | 0.000 | -0.013 | 0.000 | -0.013 | 0.000 |
| Some difficulties | -0.018 | 0.000 | -0.018 | 0.000 | -0.017 | 0.000 | -0.018 | 0.000 | -0.017 | 0.000 |
| Significant difficulties | -0.029 | 0.000 | -0.030 | 0.000 | -0.028 | 0.000 | -0.030 | 0.000 | -0.029 | 0.000 |
| *Trying on and buying clothes (OP* | | | | | | | | | | |
| Limited difficulties | -0.009 | 0.048 | -0.009 | 0.048 | -0.009 | 0.055 | -0.009 | 0.047 | -0.009 | 0.052 |
| Some difficulties | -0.001 | 0.851 | -0.001 | 0.795 | -0.001 | 0.743 | -0.001 | 0.871 | -0.001 | 0.810 |
| Significant difficulties | -0.005 | 0.250 | -0.006 | 0.165 | -0.006 | 0.130 | -0.005 | 0.187 | -0.006 | 0.146 |
| *Bathing in public places (beach, p* | | | | | | | | | | |
| Limited difficulties | 0.011 | 0.005 | 0.010 | 0.007 | 0.011 | 0.005 | 0.010 | 0.007 | 0.011 | 0.005 |
| Some difficulties | 0.019 | 0.000 | 0.018 | 0.000 | 0.018 | 0.000 | 0.017 | 0.000 | 0.017 | 0.000 |
| Significant difficulties | 0.016 | 0.000 | 0.015 | 0.000 | 0.015 | 0.000 | 0.014 | 0.000 | 0.014 | 0.000 |
| *Intimate relations (OP8)* | | | | | | | | | | |
| Limited difficulties | -0.011 | 0.000 | -0.011 | 0.000 | -0.011 | 0.000 | -0.010 | 0.000 | -0.011 | 0.000 |
| Some difficulties | -0.025 | 0.000 | -0.025 | 0.000 | -0.026 | 0.000 | -0.024 | 0.000 | -0.025 | 0.000 |
| Significant difficulties | -0.049 | 0.000 | -0.048 | 0.000 | -0.048 | 0.000 | -0.046 | 0.000 | -0.046 | 0.000 |
| **Patient characteristics** | | | | | | | | | | |
| *Age [a]* | | | | | | | | | | |
| 36-45 yrs | - | - | 0.000 | 0.737 | -0.001 | 0.590 | 0.004 | 0.029 | 0.002 | 0.203 |
| 46-55 yrs | - | - | -0.007 | 0.000 | -0.009 | 0.000 | -0.001 | 0.428 | -0.004 | 0.044 |
| 56-65 yrs | - | - | -0.013 | 0.000 | -0.014 | 0.000 | -0.005 | 0.060 | -0.007 | 0.005 |
| 65+ | - | - | -0.012 | 0.136 | -0.015 | 0.075 | -0.004 | 0.653 | -0.006 | 0.436 |
| Man [b] | - | - | -0.001 | 0.416 | 0.001 | 0.542 | 0.000 | 0.798 | 0.002 | 0.179 |
| *BMI [d]* | | | | | | | | | | |
| 40-44 | - | - | - | - | -0.008 | 0.000 | - | - | -0.007 | 0.000 |
| 45-49 | - | - | - | - | -0.013 | 0.000 | - | - | -0.013 | 0.000 |
| 50+ | - | - | - | - | -0.024 | 0.000 | - | - | -0.022 | 0.000 |
| *Comobidity e* | | | | | | | | | | |
| Sleep apnea | - | - | - | - | - | - | -0.015 | 0.000 | -0.014 | 0.000 |
| Hypertension | - | - | - | - | - | - | -0.002 | 0.231 | -0.001 | 0.496 |
| Diabetes | - | - | - | - | - | - | -0.007 | 0.001 | -0.007 | 0.001 |
| Dyslipidemia | - | - | - | - | - | - | -0.003 | 0.252 | -0.004 | 0.130 |
| Depression | - | - | - | - | - | - | -0.029 | 0.000 | -0.029 | 0.000 |
| **Goodness of fit** | | | | | | | | | | |
| Adj-Rsq, or pseudo R2 | -0.309 | | -0.310 | | -0.313 | | -0.321 | | -0.324 | |
| AIC | -47611 | | -47651 | | -47763 | | -48030 | | -48129 | |
| BIC | -47395 | | -47394 | | -47481 | | -47732 | | -47805 | |
| **Validation** | | | | | | | | | | |
| *MAE* | | | | | | | | | | |
| Training data (80%) | 0.085 | | 0.085 | | 0.085 | | 0.085 | | 0.084 | |
| Validating data (20%) | 0.084 | | 0.083 | | 0.083 | | 0.082 | | 0.082 | |
| Baseline | 0.085 | | 0.085 | | 0.085 | | 0.084 | | 0.084 | |
| 1-year | 0.110 | | 0.110 | | 0.107 | | 0.108 | | 0.105 | |
| 2-year | 0.110 | | 0.110 | | 0.108 | | 0.108 | | 0.106 | |
| 5-year | 0.108 | | 0.108 | | 0.107 | | 0.106 | | 0.105 | |
| RMSE | | | | | | | | | | |
| Training data (80%) | 0.106 | | 0.106 | | 0.106 | | 0.106 | | 0.105 | |
| Validating data (20%) | 0.104 | | 0.104 | | 0.104 | | 0.103 | | 0.103 | |
| Baseline | 0.106 | | 0.106 | | 0.106 | | 0.105 | | 0.105 | |
| 1-year | 0.129 | | 0.128 | | 0.126 | | 0.126 | | 0.124 | |
| 2-year | 0.130 | | 0.130 | | 0.128 | | 0.127 | | 0.126 | |
| 5-year | 0.129 | | 0.129 | | 0.128 | | 0.126 | | 0.126 | |
| RAE | | | | | | | | | | |
| Training data (80%) | 0.804 | | 0.803 | | 0.802 | | 0.798 | | 0.796 | |
| Validating data (20%) | 0.791 | | 0.789 | | 0.788 | | 0.782 | | 0.781 | |
| Baseline | 0.802 | | 0.801 | | 0.799 | | 0.795 | | 0.793 | |
| 1-year | 0.876 | | 0.875 | | 0.871 | | 0.861 | | 0.856 | |
| 2-year | 0.857 | | 0.857 | | 0.853 | | 0.841 | | 0.837 | |
| 5-year | 0.826 | | 0.825 | | 0.823 | | 0.808 | | 0.805 | |
| RRSE | | | | | | | | | | |
| Training data (80%) | 0.826 | | 0.825 | | 0.823 | | 0.819 | | 0.818 | |
| Validating data (20%) | 0.815 | | 0.813 | | 0.812 | | 0.807 | | 0.806 | |
| Baseline | 0.824 | | 0.823 | | 0.821 | | 0.817 | | 0.816 | |
| 1-year | 0.889 | | 0.888 | | 0.884 | | 0.875 | | 0.871 | |
| 2-year | 0.870 | | 0.870 | | 0.868 | | 0.855 | | 0.852 | |
| 5-year | 0.847 | | 0.846 | | 0.845 | | 0.831 | | 0.829 | |

| | Mapping algorithm based on 1-year follow-up data | | | | | | | | | |
|---|---|---|---|---|---|---|---|---|---|---|
| | Model 1 | | Model 2 | | Model 3 | | Model 4 | | Model 5 | |
| | coefficient | p-Value | coefficient | p-Value | coefficient | p-Value | coefficient | p-Value | coefficient | p-Value |
| **Intercept** | 0.862 | 0.000 | 0.869 | 0.000 | 0.869 | 0.000 | 0.875 | 0.000 | 0.876 | 0.000 |
| **OP dimensions [a]** | | | | | | | | | | |
| *Private gatherings in my own hon* | | | | | | | | | | |
| Limited difficulties | -0.023 | 0.000 | -0.023 | 0.000 | -0.024 | 0.000 | -0.021 | 0.000 | -0.021 | 0.000 |
| Some difficulties | -0.036 | 0.000 | -0.037 | 0.000 | -0.038 | 0.000 | -0.035 | 0.000 | -0.035 | 0.000 |
| Significant difficulties | -0.081 | 0.000 | -0.081 | 0.000 | -0.081 | 0.000 | -0.074 | 0.000 | -0.075 | 0.000 |
| *Private gatherings in a friend's or* | | | | | | | | | | |
| Limited difficulties | -0.017 | 0.000 | -0.017 | 0.000 | -0.016 | 0.000 | -0.015 | 0.000 | -0.015 | 0.000 |
| Some difficulties | -0.024 | 0.000 | -0.024 | 0.000 | -0.023 | 0.000 | -0.018 | 0.000 | -0.018 | 0.000 |
| Significant difficulties | -0.036 | 0.000 | -0.036 | 0.000 | -0.035 | 0.000 | -0.026 | 0.003 | -0.026 | 0.003 |
| *Going to a restaurant (OP3)* | | | | | | | | | | |
| Limited difficulties | -0.001 | 0.639 | -0.002 | 0.561 | -0.002 | 0.588 | -0.004 | 0.148 | -0.004 | 0.149 |
| Some difficulties | -0.009 | 0.009 | -0.010 | 0.005 | -0.010 | 0.007 | -0.013 | 0.000 | -0.013 | 0.000 |
| Significant difficulties | -0.030 | 0.000 | -0.030 | 0.000 | -0.030 | 0.000 | -0.036 | 0.000 | -0.037 | 0.000 |
| *Going to community activities, co* | | | | | | | | | | |
| Limited difficulties | -0.036 | 0.000 | -0.036 | 0.000 | -0.036 | 0.000 | -0.034 | 0.000 | -0.034 | 0.000 |
| Some difficulties | -0.078 | 0.000 | -0.077 | 0.000 | -0.077 | 0.000 | -0.072 | 0.000 | -0.072 | 0.000 |
| Significant difficulties | -0.101 | 0.000 | -0.099 | 0.000 | -0.100 | 0.000 | -0.091 | 0.000 | -0.091 | 0.000 |
| *Vacations away from home (OP5,* | | | | | | | | | | |
| Limited difficulties | -0.014 | 0.000 | -0.014 | 0.000 | -0.014 | 0.000 | -0.013 | 0.000 | -0.013 | 0.000 |
| Some difficulties | -0.015 | 0.000 | -0.017 | 0.000 | -0.016 | 0.000 | -0.017 | 0.000 | -0.017 | 0.000 |
| Significant difficulties | -0.014 | 0.036 | -0.017 | 0.012 | -0.016 | 0.016 | -0.020 | 0.003 | -0.020 | 0.004 |
| *Trying on and buying clothes (OP* | | | | | | | | | | |
| Limited difficulties | -0.019 | 0.000 | -0.020 | 0.000 | -0.020 | 0.000 | -0.018 | 0.000 | -0.018 | 0.000 |
| Some difficulties | -0.028 | 0.000 | -0.028 | 0.000 | -0.028 | 0.000 | -0.025 | 0.000 | -0.024 | 0.000 |
| Significant difficulties | -0.031 | 0.000 | -0.031 | 0.000 | -0.029 | 0.000 | -0.026 | 0.000 | -0.026 | 0.000 |
| *Bathing in public places (beach, p* | | | | | | | | | | |
| Limited difficulties | -0.006 | 0.025 | -0.006 | 0.018 | -0.007 | 0.012 | -0.007 | 0.007 | -0.007 | 0.008 |
| Some difficulties | -0.006 | 0.015 | -0.007 | 0.008 | -0.007 | 0.005 | -0.008 | 0.001 | -0.008 | 0.002 |
| Significant difficulties | -0.012 | 0.001 | -0.012 | 0.000 | -0.013 | 0.000 | -0.015 | 0.000 | -0.014 | 0.000 |
| *Intimate relations (OP8)* | | | | | | | | | | |
| Limited difficulties | -0.022 | 0.000 | -0.022 | 0.000 | -0.022 | 0.000 | -0.021 | 0.000 | -0.021 | 0.000 |
| Some difficulties | -0.037 | 0.000 | -0.036 | 0.000 | -0.037 | 0.000 | -0.034 | 0.000 | -0.034 | 0.000 |
| Significant difficulties | -0.044 | 0.000 | -0.043 | 0.000 | -0.043 | 0.000 | -0.040 | 0.000 | -0.041 | 0.000 |
| **Patient characteristics** | | | | | | | | | | |
| *Age [a]* | | | | | | | | | | |
| 36-45 yrs | - | - | -0.007 | 0.002 | -0.007 | 0.001 | -0.003 | 0.147 | -0.003 | 0.137 |
| 46-55 yrs | - | - | -0.014 | 0.000 | -0.014 | 0.000 | -0.007 | 0.001 | -0.008 | 0.001 |
| 56-65 yrs | - | - | -0.023 | 0.000 | -0.024 | 0.000 | -0.014 | 0.000 | -0.014 | 0.000 |
| 65+ | - | - | -0.016 | 0.070 | -0.015 | 0.082 | -0.006 | 0.504 | -0.006 | 0.478 |
| Man [b] | - | - | 0.014 | 0.000 | 0.014 | 0.000 | 0.012 | 0.000 | 0.012 | 0.000 |
| *BMI [d]* | | | | | | | | | | |
| 40-44 | - | - | - | - | -0.017 | 0.014 | - | - | -0.010 | 0.128 |
| 45-49 | - | - | - | - | -0.038 | 0.015 | - | - | -0.028 | 0.070 |
| 50+ | - | - | - | - | 0.010 | 0.673 | - | - | 0.012 | 0.627 |
| *Comobidity e* | | | | | | | | | | |
| Sleep apnea | - | - | - | - | - | - | -0.022 | 0.000 | -0.021 | 0.000 |
| Hypertension | - | - | - | - | - | - | -0.011 | 0.000 | -0.011 | 0.000 |
| Diabetes | - | - | - | - | - | - | -0.012 | 0.006 | -0.012 | 0.007 |
| Dyslipidemia | - | - | - | - | - | - | 0.000 | 0.942 | 0.000 | 0.940 |
| Depression | - | - | - | - | - | - | -0.072 | 0.000 | -0.072 | 0.000 |
| **Goodness of fit** | | | | | | | | | | |
| Adj-Rsq, or pseudo R2 | -0.407 | | -0.414 | | -0.404 | | -0.460 | | -0.460 | |
| AIC | -23307 | | -23411 | | -23249 | | -24173 | | -24158 | |
| BIC | -23099 | | -23163 | | -22978 | | -23886 | | -23847 | |
| **Validation** | | | | | | | | | | |
| *MAE* | | | | | | | | | | |
| Training data (80%) | 0.091 | | 0.091 | | 0.091 | | 0.088 | | 0.088 | |
| Validating data (20%) | 0.092 | | 0.091 | | 0.091 | | 0.089 | | 0.089 | |
| Baseline | 0.086 | | 0.086 | | 0.088 | | 0.086 | | 0.087 | |
| 1-year | 0.091 | | 0.091 | | 0.091 | | 0.089 | | 0.089 | |
| 2-year | 0.095 | | 0.094 | | 0.094 | | 0.092 | | 0.092 | |
| 5-year | 0.099 | | 0.098 | | 0.098 | | 0.095 | | 0.094 | |
| RMSE | | | | | | | | | | |
| Training data (80%) | 0.117 | | 0.117 | | 0.117 | | 0.114 | | 0.114 | |
| Validating data (20%) | 0.117 | | 0.117 | | 0.117 | | 0.114 | | 0.114 | |
| Baseline | 0.110 | | 0.110 | | 0.112 | | 0.110 | | 0.110 | |
| 1-year | 0.117 | | 0.117 | | 0.117 | | 0.114 | | 0.114 | |
| 2-year | 0.122 | | 0.122 | | 0.121 | | 0.118 | | 0.118 | |
| 5-year | 0.128 | | 0.127 | | 0.126 | | 0.123 | | 0.123 | |
| RAE | | | | | | | | | | |
| Training data (80%) | 0.812 | | 0.808 | | 0.808 | | 0.787 | | 0.788 | |
| Validating data (20%) | 0.809 | | 0.805 | | 0.805 | | 0.787 | | 0.787 | |
| Baseline | 0.817 | | 0.818 | | 0.829 | | 0.818 | | 0.824 | |
| 1-year | 0.811 | | 0.808 | | 0.807 | | 0.787 | | 0.787 | |
| 2-year | 0.789 | | 0.785 | | 0.785 | | 0.761 | | 0.762 | |
| 5-year | 0.766 | | 0.759 | | 0.760 | | 0.738 | | 0.737 | |
| RRSE | | | | | | | | | | |
| Training data (80%) | 0.851 | | 0.848 | | 0.847 | | 0.829 | | 0.829 | |
| Validating data (20%) | 0.844 | | 0.842 | | 0.842 | | 0.824 | | 0.825 | |
| Baseline | 0.852 | | 0.853 | | 0.859 | | 0.852 | | 0.855 | |
| 1-year | 0.849 | | 0.847 | | 0.846 | | 0.828 | | 0.828 | |
| 2-year | 0.836 | | 0.833 | | 0.834 | | 0.812 | | 0.812 | |
| 5-year | 0.825 | | 0.821 | | 0.821 | | 0.800 | | 0.798 | |

| | Mapping algorithm based on 2-year follow-up data | | | | | | | | | |
|---|---|---|---|---|---|---|---|---|---|---|
| | Model 1 | | Model 2 | | Model 3 | | Model 4 | | Model 5 | |
| | coefficient | p-Value | coefficient | p-Value | coefficient | p-Value | coefficient | p-Value | coefficient | p-Value |
| **Intercept** | 0.853 | 0.000 | 0.860 | 0.000 | 0.860 | 0.000 | 0.866 | 0.000 | 0.866 | 0.000 |
| **OP dimensions [a]** | | | | | | | | | | |
| *Private gatherings in my own hon* | | | | | | | | | | |
| Limited difficulties | -0.023 | 0.000 | -0.023 | 0.000 | -0.024 | 0.000 | -0.023 | 0.000 | -0.023 | 0.000 |
| Some difficulties | -0.039 | 0.000 | -0.039 | 0.000 | -0.040 | 0.000 | -0.037 | 0.000 | -0.037 | 0.000 |
| Significant difficulties | -0.077 | 0.000 | -0.076 | 0.000 | -0.077 | 0.000 | -0.073 | 0.000 | -0.073 | 0.000 |
| *Private gatherings in a friend's or* | | | | | | | | | | |
| Limited difficulties | -0.023 | 0.000 | -0.023 | 0.000 | -0.023 | 0.000 | -0.022 | 0.000 | -0.022 | 0.000 |
| Some difficulties | -0.030 | 0.000 | -0.032 | 0.000 | -0.033 | 0.000 | -0.026 | 0.000 | -0.026 | 0.000 |
| Significant difficulties | -0.036 | 0.000 | -0.038 | 0.000 | -0.038 | 0.000 | -0.026 | 0.011 | -0.026 | 0.012 |
| *Going to a restaurant (OP3)* | | | | | | | | | | |
| Limited difficulties | 0.004 | 0.406 | 0.003 | 0.455 | 0.003 | 0.543 | 0.001 | 0.768 | 0.001 | 0.758 |
| Some difficulties | 0.006 | 0.256 | 0.005 | 0.292 | 0.005 | 0.271 | 0.001 | 0.853 | 0.002 | 0.756 |
| Significant difficulties | -0.004 | 0.675 | -0.004 | 0.610 | -0.005 | 0.549 | -0.012 | 0.142 | -0.012 | 0.140 |
| *Going to community activities, co* | | | | | | | | | | |
| Limited difficulties | -0.040 | 0.000 | -0.039 | 0.000 | -0.039 | 0.000 | -0.036 | 0.000 | -0.036 | 0.000 |
| Some difficulties | -0.081 | 0.000 | -0.079 | 0.000 | -0.079 | 0.000 | -0.074 | 0.000 | -0.073 | 0.000 |
| Significant difficulties | -0.125 | 0.000 | -0.123 | 0.000 | -0.122 | 0.000 | -0.109 | 0.000 | -0.109 | 0.000 |
| *Vacations away from home (OP5,* | | | | | | | | | | |
| Limited difficulties | -0.011 | 0.013 | -0.012 | 0.008 | -0.011 | 0.017 | -0.010 | 0.020 | -0.010 | 0.026 |
| Some difficulties | -0.015 | 0.005 | -0.016 | 0.003 | -0.015 | 0.005 | -0.016 | 0.002 | -0.016 | 0.002 |
| Significant difficulties | -0.003 | 0.706 | -0.005 | 0.543 | -0.005 | 0.529 | -0.008 | 0.321 | -0.008 | 0.298 |
| *Trying on and buying clothes (OP* | | | | | | | | | | |
| Limited difficulties | -0.016 | 0.000 | -0.016 | 0.000 | -0.016 | 0.000 | -0.015 | 0.000 | -0.015 | 0.000 |
| Some difficulties | -0.027 | 0.000 | -0.027 | 0.000 | -0.026 | 0.000 | -0.026 | 0.000 | -0.025 | 0.000 |
| Significant difficulties | -0.031 | 0.000 | -0.031 | 0.000 | -0.029 | 0.000 | -0.029 | 0.000 | -0.027 | 0.000 |
| *Bathing in public places (beach, p* | | | | | | | | | | |
| Limited difficulties | -0.012 | 0.001 | -0.012 | 0.001 | -0.012 | 0.001 | -0.013 | 0.001 | -0.013 | 0.001 |
| Some difficulties | -0.009 | 0.016 | -0.009 | 0.010 | -0.010 | 0.007 | -0.011 | 0.002 | -0.011 | 0.003 |
| Significant difficulties | -0.010 | 0.032 | -0.011 | 0.016 | -0.011 | 0.017 | -0.014 | 0.002 | -0.013 | 0.002 |
| *Intimate relations (OP8)* | | | | | | | | | | |
| Limited difficulties | -0.022 | 0.000 | -0.022 | 0.000 | -0.021 | 0.000 | -0.018 | 0.000 | -0.018 | 0.000 |
| Some difficulties | -0.036 | 0.000 | -0.035 | 0.000 | -0.035 | 0.000 | -0.030 | 0.000 | -0.031 | 0.000 |
| Significant difficulties | -0.050 | 0.000 | -0.048 | 0.000 | -0.048 | 0.000 | -0.043 | 0.000 | -0.044 | 0.000 |
| **Patient characteristics** | | | | | | | | | | |
| *Age [a]* | | | | | | | | | | |
| 36-45 yrs | - | - | -0.009 | 0.003 | -0.009 | 0.004 | -0.004 | 0.177 | -0.004 | 0.160 |
| 46-55 yrs | - | - | -0.014 | 0.000 | -0.013 | 0.000 | -0.005 | 0.098 | -0.005 | 0.092 |
| 56-65 yrs | - | - | -0.020 | 0.000 | -0.020 | 0.000 | -0.010 | 0.006 | -0.011 | 0.005 |
| 65+ | - | - | -0.016 | 0.059 | -0.017 | 0.039 | -0.008 | 0.359 | -0.008 | 0.333 |
| Man [b] | - | - | 0.016 | 0.000 | 0.016 | 0.000 | 0.013 | 0.000 | 0.013 | 0.000 |
| *BMI [d]* | | | | | | | | | | |
| 40-44 | - | - | - | - | -0.026 | 0.003 | - | - | -0.019 | 0.024 |
| 45-49 | - | - | - | - | -0.025 | 0.160 | - | - | -0.016 | 0.352 |
| 50+ | - | - | - | - | -0.115 | 0.001 | - | - | -0.118 | 0.001 |
| *Comobidity e* | | | | | | | | | | |
| Sleep apnea | - | - | - | - | - | - | -0.021 | 0.002 | -0.018 | 0.005 |
| Hypertension | - | - | - | - | - | - | -0.009 | 0.005 | -0.009 | 0.007 |
| Diabetes | - | - | - | - | - | - | -0.007 | 0.145 | -0.008 | 0.119 |
| Dyslipidemia | - | - | - | - | - | - | -0.005 | 0.338 | -0.004 | 0.366 |
| Depression | - | - | - | - | - | - | -0.078 | 0.000 | -0.078 | 0.000 |
| **Goodness of fit** | | | | | | | | | | |
| Adj-Rsq, or pseudo R2 | -0.481 | | -0.488 | | -0.455 | | -0.527 | | -0.523 | |
| AIC | -14785 | | -14842 | | -14503 | | -15221 | | -15177 | |
| BIC | -14589 | | -14608 | | -14248 | | -14951 | | -14884 | |
| **Validation** | | | | | | | | | | |
| *MAE* | | | | | | | | | | |
| Training data (80%) | 0.096 | | 0.095 | | 0.095 | | 0.092 | | 0.092 | |
| Validating data (20%) | 0.096 | | 0.096 | | 0.095 | | 0.092 | | 0.092 | |
| Baseline | 0.087 | | 0.087 | | 0.092 | | 0.087 | | 0.091 | |
| 1-year | 0.093 | | 0.092 | | 0.092 | | 0.090 | | 0.090 | |
| 2-year | 0.096 | | 0.095 | | 0.095 | | 0.092 | | 0.092 | |
| 5-year | 0.098 | | 0.098 | | 0.098 | | 0.095 | | 0.094 | |
| RMSE | | | | | | | | | | |
| Training data (80%) | 0.121 | | 0.121 | | 0.121 | | 0.118 | | 0.118 | |
| Validating data (20%) | 0.121 | | 0.121 | | 0.120 | | 0.117 | | 0.117 | |
| Baseline | 0.110 | | 0.110 | | 0.116 | | 0.110 | | 0.115 | |
| 1-year | 0.117 | | 0.117 | | 0.117 | | 0.115 | | 0.115 | |
| 2-year | 0.121 | | 0.121 | | 0.121 | | 0.117 | | 0.117 | |
| 5-year | 0.126 | | 0.125 | | 0.125 | | 0.122 | | 0.121 | |
| RAE | | | | | | | | | | |
| Training data (80%) | 0.789 | | 0.786 | | 0.788 | | 0.764 | | 0.764 | |
| Validating data (20%) | 0.781 | | 0.779 | | 0.775 | | 0.751 | | 0.751 | |
| Baseline | 0.820 | | 0.821 | | 0.855 | | 0.823 | | 0.854 | |
| 1-year | 0.811 | | 0.809 | | 0.809 | | 0.788 | | 0.789 | |
| 2-year | 0.787 | | 0.785 | | 0.785 | | 0.761 | | 0.761 | |
| 5-year | 0.761 | | 0.757 | | 0.759 | | 0.735 | | 0.734 | |
| RRSE | | | | | | | | | | |
| Training data (80%) | 0.836 | | 0.834 | | 0.835 | | 0.813 | | 0.813 | |
| Validating data (20%) | 0.826 | | 0.824 | | 0.819 | | 0.798 | | 0.797 | |
| Baseline | 0.851 | | 0.853 | | 0.875 | | 0.853 | | 0.876 | |
| 1-year | 0.850 | | 0.848 | | 0.848 | | 0.830 | | 0.830 | |
| 2-year | 0.834 | | 0.832 | | 0.831 | | 0.810 | | 0.810 | |
| 5-year | 0.822 | | 0.818 | | 0.819 | | 0.796 | | 0.795 | |

| | Mapping algorithm based on 5-year follow-up data | | | | | | | | | |
|---|---|---|---|---|---|---|---|---|---|---|
| | Model 1 | | Model 2 | | Model 3 | | Model 4 | | Model 5 | |
| | coefficient | p-Value | coefficient | p-Value | coefficient | p-Value | coefficient | p-Value | coefficient | p-Value |
| **Intercept** | 0.832 | 0.000 | 0.845 | 0.000 | 0.845 | 0.000 | 0.854 | 0.000 | 0.853 | 0.000 |
| **OP dimensions [a]** | | | | | | | | | | |
| *Private gatherings in my own hon* | | | | | | | | | | |
| Limited difficulties | -0.016 | 0.022 | -0.016 | 0.022 | -0.017 | 0.014 | -0.012 | 0.068 | -0.015 | 0.029 |
| Some difficulties | -0.032 | 0.001 | -0.031 | 0.001 | -0.034 | 0.000 | -0.029 | 0.001 | -0.031 | 0.001 |
| Significant difficulties | -0.084 | 0.000 | -0.083 | 0.000 | -0.088 | 0.000 | -0.078 | 0.000 | -0.080 | 0.000 |
| *Private gatherings in a friend's or* | | | | | | | | | | |
| Limited difficulties | -0.018 | 0.016 | -0.019 | 0.008 | -0.018 | 0.016 | -0.018 | 0.012 | -0.016 | 0.021 |
| Some difficulties | -0.025 | 0.010 | -0.026 | 0.006 | -0.024 | 0.012 | -0.020 | 0.032 | -0.019 | 0.044 |
| Significant difficulties | -0.040 | 0.005 | -0.040 | 0.006 | -0.037 | 0.011 | -0.030 | 0.039 | -0.026 | 0.068 |
| *Going to a restaurant (OP3)* | | | | | | | | | | |
| Limited difficulties | 0.000 | 0.945 | -0.001 | 0.913 | 0.000 | 0.985 | -0.002 | 0.796 | -0.001 | 0.893 |
| Some difficulties | 0.002 | 0.835 | 0.001 | 0.891 | 0.002 | 0.850 | -0.004 | 0.627 | -0.002 | 0.789 |
| Significant difficulties | -0.005 | 0.716 | -0.006 | 0.624 | -0.010 | 0.480 | -0.017 | 0.191 | -0.019 | 0.151 |
| *Going to community activities, co* | | | | | | | | | | |
| Limited difficulties | -0.029 | 0.000 | -0.029 | 0.000 | -0.030 | 0.000 | -0.029 | 0.000 | -0.028 | 0.000 |
| Some difficulties | -0.061 | 0.000 | -0.061 | 0.000 | -0.060 | 0.000 | -0.053 | 0.000 | -0.053 | 0.000 |
| Significant difficulties | -0.103 | 0.000 | -0.102 | 0.000 | -0.096 | 0.000 | -0.082 | 0.000 | -0.081 | 0.000 |
| *Vacations away from home (OP5,* | | | | | | | | | | |
| Limited difficulties | -0.015 | 0.030 | -0.013 | 0.047 | -0.012 | 0.074 | -0.011 | 0.089 | -0.012 | 0.069 |
| Some difficulties | -0.014 | 0.073 | -0.014 | 0.077 | -0.014 | 0.087 | -0.015 | 0.057 | -0.017 | 0.035 |
| Significant difficulties | -0.020 | 0.087 | -0.022 | 0.065 | -0.015 | 0.217 | -0.015 | 0.198 | -0.014 | 0.231 |
| *Trying on and buying clothes (OP* | | | | | | | | | | |
| Limited difficulties | -0.021 | 0.000 | -0.022 | 0.000 | -0.022 | 0.000 | -0.021 | 0.000 | -0.020 | 0.000 |
| Some difficulties | -0.030 | 0.000 | -0.030 | 0.000 | -0.030 | 0.000 | -0.028 | 0.000 | -0.028 | 0.000 |
| Significant difficulties | -0.038 | 0.000 | -0.038 | 0.000 | -0.041 | 0.000 | -0.038 | 0.000 | -0.039 | 0.000 |
| *Bathing in public places (beach, p* | | | | | | | | | | |
| Limited difficulties | 0.000 | 0.930 | 0.000 | 0.975 | 0.002 | 0.729 | 0.001 | 0.896 | 0.001 | 0.824 |
| Some difficulties | -0.002 | 0.705 | -0.003 | 0.609 | -0.003 | 0.570 | -0.005 | 0.415 | -0.004 | 0.465 |
| Significant difficulties | -0.009 | 0.222 | -0.010 | 0.147 | -0.010 | 0.176 | -0.010 | 0.146 | -0.009 | 0.191 |
| *Intimate relations (OP8)* | | | | | | | | | | |
| Limited difficulties | -0.033 | 0.000 | -0.032 | 0.000 | -0.030 | 0.000 | -0.029 | 0.000 | -0.029 | 0.000 |
| Some difficulties | -0.045 | 0.000 | -0.044 | 0.000 | -0.044 | 0.000 | -0.040 | 0.000 | -0.040 | 0.000 |
| Significant difficulties | -0.060 | 0.000 | -0.058 | 0.000 | -0.059 | 0.000 | -0.055 | 0.000 | -0.055 | 0.000 |
| **Patient characteristics** | | | | | | | | | | |
| *Age [a]* | | | | | | | | | | |
| 36-45 yrs | - | - | -0.016 | 0.003 | -0.017 | 0.002 | -0.015 | 0.006 | -0.014 | 0.007 |
| 46-55 yrs | - | - | -0.020 | 0.000 | -0.019 | 0.000 | -0.014 | 0.008 | -0.013 | 0.011 |
| 56-65 yrs | - | - | -0.026 | 0.000 | -0.027 | 0.000 | -0.020 | 0.001 | -0.020 | 0.001 |
| 65+ | - | - | -0.023 | 0.008 | -0.025 | 0.005 | -0.019 | 0.035 | -0.020 | 0.025 |
| Man [b] | - | - | 0.022 | 0.000 | 0.024 | 0.000 | 0.019 | 0.000 | 0.019 | 0.000 |
| *BMI [d]* | | | | | | | | | | |
| 40-44 | - | - | - | - | -0.016 | 0.107 | - | - | -0.013 | 0.198 |
| 45-49 | - | - | - | - | -0.020 | 0.326 | - | - | -0.013 | 0.503 |
| 50+ | - | - | - | - | 0.040 | 0.329 | - | - | 0.051 | 0.202 |
| *Comobidity e* | | | | | | | | | | |
| Sleep apnea | - | - | - | - | - | - | 0.000 | 0.981 | 0.000 | 0.975 |
| Hypertension | - | - | - | - | - | - | -0.009 | 0.060 | -0.009 | 0.062 |
| Diabetes | - | - | - | - | - | - | -0.012 | 0.092 | -0.011 | 0.144 |
| Dyslipidemia | - | - | - | - | - | - | 0.001 | 0.934 | 0.002 | 0.820 |
| Depression | - | - | - | - | - | - | -0.086 | 0.000 | -0.086 | 0.000 |
| **Goodness of fit** | | | | | | | | | | |
| Adj-Rsq, or pseudo R2 | -0.564 | | -0.576 | | -0.520 | | -0.610 | | -0.607 | |
| AIC | -6528 | | -6568 | | -6324 | | -6700 | | -6682 | |
| BIC | -6354 | | -6360 | | -6098 | | -6460 | | -6423 | |
| **Validation** | | | | | | | | | | |
| *MAE* | | | | | | | | | | |
| Training data (80%) | 0.099 | | 0.099 | | 0.098 | | 0.095 | | 0.095 | |
| Validating data (20%) | 0.100 | | 0.099 | | 0.099 | | 0.096 | | 0.096 | |
| Baseline | 0.091 | | 0.090 | | 0.092 | | 0.089 | | 0.091 | |
| 1-year | 0.098 | | 0.097 | | 0.097 | | 0.094 | | 0.094 | |
| 2-year | 0.100 | | 0.099 | | 0.099 | | 0.096 | | 0.096 | |
| 5-year | 0.099 | | 0.099 | | 0.099 | | 0.095 | | 0.095 | |
| RMSE | | | | | | | | | | |
| Training data (80%) | 0.124 | | 0.124 | | 0.123 | | 0.120 | | 0.119 | |
| Validating data (20%) | 0.124 | | 0.123 | | 0.123 | | 0.121 | | 0.121 | |
| Baseline | 0.115 | | 0.114 | | 0.117 | | 0.113 | | 0.114 | |
| 1-year | 0.120 | | 0.119 | | 0.119 | | 0.117 | | 0.117 | |
| 2-year | 0.123 | | 0.122 | | 0.122 | | 0.119 | | 0.119 | |
| 5-year | 0.124 | | 0.124 | | 0.123 | | 0.120 | | 0.120 | |
| RAE | | | | | | | | | | |
| Training data (80%) | 0.764 | | 0.761 | | 0.761 | | 0.733 | | 0.732 | |
| Validating data (20%) | 0.761 | | 0.758 | | 0.758 | | 0.739 | | 0.738 | |
| Baseline | 0.844 | | 0.841 | | 0.858 | | 0.841 | | 0.855 | |
| 1-year | 0.823 | | 0.818 | | 0.818 | | 0.797 | | 0.797 | |
| 2-year | 0.799 | | 0.796 | | 0.797 | | 0.772 | | 0.772 | |
| 5-year | 0.764 | | 0.760 | | 0.760 | | 0.734 | | 0.733 | |
| RRSE | | | | | | | | | | |
| Training data (80%) | 0.815 | | 0.812 | | 0.812 | | 0.787 | | 0.786 | |
| Validating data (20%) | 0.819 | | 0.815 | | 0.816 | | 0.800 | | 0.799 | |
| Baseline | 0.859 | | 0.861 | | 0.873 | | 0.860 | | 0.874 | |
| 1-year | 0.856 | | 0.854 | | 0.853 | | 0.837 | | 0.837 | |
| 2-year | 0.838 | | 0.836 | | 0.836 | | 0.815 | | 0.815 | |
| 5-year | 0.816 | | 0.812 | | 0.813 | | 0.790 | | 0.788 | |

**Reference group**
**a no difficulties**
**b 18-34 years**
**c Women**
**d <=39**
**e No disease**

# Table S7B. Mapping algorithm based on beta regression models, Type B, transfored SF-6D index applied

**Mapping algorithm based on baseline data**

| | Model 1 coefficient | Model 1 p-Value | Model 2 coefficient | Model 2 p-Value | Model 3 coefficient | Model 3 p-Value | Model 4 coefficient | Model 4 p-Value | Model 5 coefficient | Model 5 p-Value |
|---|---|---|---|---|---|---|---|---|---|---|
| **Intercept** | 1.425 | 0.000 | 1.444 | 0.000 | 1.480 | 0.000 | 1.451 | 0.000 | 1.486 | 0.000 |
| **OP dimensions** [a] | | | | | | | | | | |
| *Private gatherings in my own hor* | | | | | | | | | | |
| Limited difficulties | -0.118 | 0.000 | -0.117 | 0.000 | -0.122 | 0.000 | -0.116 | 0.000 | -0.120 | 0.000 |
| Some difficulties | -0.200 | 0.000 | -0.199 | 0.000 | -0.209 | 0.000 | -0.197 | 0.000 | -0.206 | 0.000 |
| Significant difficulties | -0.356 | 0.000 | -0.354 | 0.000 | -0.367 | 0.000 | -0.349 | 0.000 | -0.361 | 0.000 |
| *Private gatherings in a friend's or* | | | | | | | | | | |
| Limited difficulties | -0.029 | 0.074 | -0.027 | 0.095 | -0.026 | 0.109 | -0.026 | 0.110 | -0.024 | 0.125 |
| Some difficulties | -0.030 | 0.073 | -0.029 | 0.081 | -0.030 | 0.069 | -0.028 | 0.099 | -0.029 | 0.085 |
| Significant difficulties | -0.087 | 0.000 | -0.087 | 0.000 | -0.092 | 0.000 | -0.083 | 0.000 | -0.087 | 0.000 |
| *Going to a restaurant (OP3)* | | | | | | | | | | |
| Limited difficulties | -0.036 | 0.009 | -0.037 | 0.007 | -0.033 | 0.015 | -0.038 | 0.006 | -0.034 | 0.012 |
| Some difficulties | -0.020 | 0.149 | -0.025 | 0.082 | -0.017 | 0.234 | -0.025 | 0.078 | -0.018 | 0.211 |
| Significant difficulties | -0.049 | 0.002 | -0.055 | 0.001 | -0.041 | 0.010 | -0.053 | 0.001 | -0.040 | 0.012 |
| *Going to community activities, co* | | | | | | | | | | |
| Limited difficulties | -0.126 | 0.000 | -0.126 | 0.000 | -0.127 | 0.000 | -0.123 | 0.000 | -0.124 | 0.000 |
| Some difficulties | -0.233 | 0.000 | -0.232 | 0.000 | -0.230 | 0.000 | -0.227 | 0.000 | -0.225 | 0.000 |
| Significant difficulties | -0.363 | 0.000 | -0.362 | 0.000 | -0.357 | 0.000 | -0.353 | 0.000 | -0.349 | 0.000 |
| *Vacations away from home (OP5,* | | | | | | | | | | |
| Limited difficulties | -0.072 | 0.000 | -0.072 | 0.000 | -0.072 | 0.000 | -0.071 | 0.000 | -0.071 | 0.000 |
| Some difficulties | -0.090 | 0.000 | -0.090 | 0.000 | -0.088 | 0.000 | -0.091 | 0.000 | -0.089 | 0.000 |
| Significant difficulties | -0.134 | 0.000 | -0.136 | 0.000 | -0.131 | 0.000 | -0.138 | 0.000 | -0.133 | 0.000 |
| *Trying on and buying clothes (OP(* | | | | | | | | | | |
| Limited difficulties | -0.129 | 0.000 | -0.130 | 0.000 | -0.128 | 0.000 | -0.128 | 0.000 | -0.127 | 0.000 |
| Some difficulties | -0.086 | 0.000 | -0.088 | 0.000 | -0.089 | 0.000 | -0.086 | 0.000 | -0.087 | 0.000 |
| Significant difficulties | -0.109 | 0.000 | -0.113 | 0.000 | -0.115 | 0.000 | -0.111 | 0.000 | -0.114 | 0.000 |
| *Bathing in public places (beach, p* | | | | | | | | | | |
| Limited difficulties | 0.028 | 0.147 | 0.027 | 0.169 | 0.028 | 0.142 | 0.027 | 0.155 | 0.029 | 0.132 |
| Some difficulties | 0.068 | 0.000 | 0.065 | 0.000 | 0.065 | 0.000 | 0.061 | 0.000 | 0.062 | 0.000 |
| Significant difficulties | 0.055 | 0.002 | 0.050 | 0.005 | 0.051 | 0.004 | 0.046 | 0.010 | 0.047 | 0.008 |
| *Intimate relations (OP8)* | | | | | | | | | | |
| Limited difficulties | -0.078 | 0.000 | -0.076 | 0.000 | -0.078 | 0.000 | -0.074 | 0.000 | -0.075 | 0.000 |
| Some difficulties | -0.142 | 0.000 | -0.140 | 0.000 | -0.143 | 0.000 | -0.136 | 0.000 | -0.139 | 0.000 |
| Significant difficulties | -0.242 | 0.000 | -0.237 | 0.000 | -0.240 | 0.000 | -0.226 | 0.000 | -0.230 | 0.000 |
| **Patient characteristics** | | | | | | | | | | |
| *Age* [a] | | | | | | | | | | |
| 36-45 yrs | - | - | 0.005 | 0.496 | -0.002 | 0.842 | 0.019 | 0.012 | 0.012 | 0.108 |
| 46-55 yrs | - | - | -0.023 | 0.003 | -0.032 | 0.000 | 0.003 | 0.725 | -0.008 | 0.361 |
| 56-65 yrs | - | - | -0.047 | 0.000 | -0.057 | 0.000 | -0.009 | 0.431 | -0.021 | 0.080 |
| 65+ | - | - | -0.052 | 0.186 | -0.064 | 0.104 | -0.012 | 0.766 | -0.025 | 0.524 |
| Man [b] | - | - | -0.006 | 0.397 | 0.005 | 0.497 | 0.003 | 0.698 | 0.012 | 0.122 |
| *BMI* [d] | | | | | | | | | | |
| 40-44 | - | - | - | - | -0.040 | 0.000 | - | - | -0.039 | 0.000 |
| 45-49 | - | - | - | - | -0.064 | 0.000 | - | - | -0.061 | 0.000 |
| 50+ | - | - | - | - | -0.113 | 0.000 | - | - | -0.104 | 0.000 |
| *Comobidity e* | | | | | | | | | | |
| Sleep apnea | - | - | - | - | - | - | -0.074 | 0.000 | -0.065 | 0.000 |
| Hypertension | - | - | - | - | - | - | -0.011 | 0.169 | -0.007 | 0.387 |
| Diabetes | - | - | - | - | - | - | -0.035 | 0.001 | -0.036 | 0.000 |
| Dyslipidemia | - | - | - | - | - | - | -0.015 | 0.181 | -0.019 | 0.092 |
| Depression | - | - | - | - | - | - | -0.129 | 0.000 | -0.128 | 0.000 |
| **Goodness of fit** | | | | | | | | | | |
| Adj-Rsq, or pseudo R2 | 0.287 | | 0.287 | | 0.290 | | 0.295 | | 0.297 | |
| AIC | -46923 | | -46947 | | -47063 | | -47301 | | -47402 | |
| BIC | -46708 | | -46690 | | -46781 | | -47002 | | -47079 | |
| **Validation** | | | | | | | | | | |
| *MAE* | | | | | | | | | | |
| Training data (80%) | 0.085 | | 0.085 | | 0.085 | | 0.085 | | 0.084 | |
| Validating data (20%) | 0.084 | | 0.083 | | 0.083 | | 0.083 | | 0.082 | |
| Baseline | 0.085 | | 0.085 | | 0.085 | | 0.084 | | 0.084 | |
| 1-year | 0.104 | | 0.103 | | 0.102 | | 0.102 | | 0.100 | |
| 2-year | 0.105 | | 0.105 | | 0.104 | | 0.103 | | 0.102 | |
| 5-year | 0.105 | | 0.105 | | 0.104 | | 0.103 | | 0.102 | |
| RMSE | | | | | | | | | | |
| Training data (80%) | 0.106 | | 0.106 | | 0.106 | | 0.106 | | 0.106 | |
| Validating data (20%) | 0.105 | | 0.104 | | 0.104 | | 0.104 | | 0.103 | |
| Baseline | 0.106 | | 0.106 | | 0.106 | | 0.105 | | 0.105 | |
| 1-year | 0.123 | | 0.123 | | 0.122 | | 0.121 | | 0.120 | |
| 2-year | 0.125 | | 0.125 | | 0.124 | | 0.123 | | 0.122 | |
| 5-year | 0.127 | | 0.127 | | 0.127 | | 0.125 | | 0.125 | |
| RAE | | | | | | | | | | |
| Training data (80%) | 0.803 | | 0.802 | | 0.800 | | 0.797 | | 0.796 | |
| Validating data (20%) | 0.791 | | 0.789 | | 0.788 | | 0.782 | | 0.781 | |
| Baseline | 0.801 | | 0.800 | | 0.798 | | 0.794 | | 0.793 | |
| 1-year | 0.855 | | 0.854 | | 0.852 | | 0.843 | | 0.841 | |
| 2-year | 0.835 | | 0.834 | | 0.833 | | 0.822 | | 0.821 | |
| 5-year | 0.805 | | 0.804 | | 0.804 | | 0.790 | | 0.789 | |
| RRSE | | | | | | | | | | |
| Training data (80%) | 0.827 | | 0.826 | | 0.824 | | 0.821 | | 0.820 | |
| Validating data (20%) | 0.817 | | 0.815 | | 0.814 | | 0.809 | | 0.808 | |
| Baseline | 0.825 | | 0.824 | | 0.822 | | 0.819 | | 0.817 | |
| 1-year | 0.872 | | 0.871 | | 0.869 | | 0.860 | | 0.859 | |
| 2-year | 0.854 | | 0.854 | | 0.853 | | 0.842 | | 0.841 | |
| 5-year | 0.834 | | 0.833 | | 0.834 | | 0.822 | | 0.821 | |

**Mapping algorithm based on 1-year follow-up data**

| | Model 1 coefficient | Model 1 p-Value | Model 2 coefficient | Model 2 p-Value | Model 3 coefficient | Model 3 p-Value | Model 4 coefficient | Model 4 p-Value | Model 5 coefficient | Model 5 p-Value |
|---|---|---|---|---|---|---|---|---|---|---|
| **Intercept** | 1.904 | 0.000 | 1.908 | 0.000 | 1.913 | 0.000 | 1.951 | 0.000 | 1.951 | 0.000 |
| **OP dimensions** [a] | | | | | | | | | | |
| *Private gatherings in my own hor* | | | | | | | | | | |
| Limited difficulties | -0.121 | 0.000 | -0.125 | 0.000 | -0.130 | 0.000 | -0.113 | 0.000 | -0.114 | 0.000 |
| Some difficulties | -0.171 | 0.000 | -0.177 | 0.000 | -0.184 | 0.000 | -0.167 | 0.000 | -0.168 | 0.000 |
| Significant difficulties | -0.350 | 0.000 | -0.349 | 0.000 | -0.352 | 0.000 | -0.316 | 0.000 | -0.318 | 0.000 |
| *Private gatherings in a friend's or* | | | | | | | | | | |
| Limited difficulties | -0.126 | 0.000 | -0.124 | 0.000 | -0.121 | 0.000 | -0.116 | 0.000 | -0.114 | 0.000 |
| Some difficulties | -0.150 | 0.000 | -0.148 | 0.000 | -0.144 | 0.000 | -0.114 | 0.000 | -0.113 | 0.000 |
| Significant difficulties | -0.174 | 0.001 | -0.172 | 0.001 | -0.170 | 0.001 | -0.113 | 0.031 | -0.111 | 0.036 |
| *Going to a restaurant (OP3)* | | | | | | | | | | |
| Limited difficulties | -0.029 | 0.133 | -0.029 | 0.128 | -0.027 | 0.159 | -0.038 | 0.047 | -0.037 | 0.048 |
| Some difficulties | -0.073 | 0.001 | -0.075 | 0.001 | -0.072 | 0.001 | -0.090 | 0.000 | -0.089 | 0.000 |
| Significant difficulties | -0.170 | 0.000 | -0.172 | 0.000 | -0.175 | 0.000 | -0.214 | 0.000 | -0.215 | 0.000 |
| *Going to community activities, co* | | | | | | | | | | |
| Limited difficulties | -0.217 | 0.000 | -0.218 | 0.000 | -0.219 | 0.000 | -0.207 | 0.000 | -0.208 | 0.000 |
| Some difficulties | -0.416 | 0.000 | -0.415 | 0.000 | -0.415 | 0.000 | -0.388 | 0.000 | -0.387 | 0.000 |
| Significant difficulties | -0.495 | 0.000 | -0.489 | 0.000 | -0.488 | 0.000 | -0.439 | 0.000 | -0.439 | 0.000 |
| *Vacations away from home (OP5,* | | | | | | | | | | |
| Limited difficulties | -0.044 | 0.036 | -0.046 | 0.029 | -0.043 | 0.042 | -0.036 | 0.087 | -0.035 | 0.097 |
| Some difficulties | -0.031 | 0.233 | -0.035 | 0.177 | -0.032 | 0.229 | -0.040 | 0.129 | -0.039 | 0.132 |
| Significant difficulties | -0.028 | 0.480 | -0.038 | 0.346 | -0.032 | 0.437 | -0.054 | 0.174 | -0.052 | 0.193 |
| *Trying on and buying clothes (OP(* | | | | | | | | | | |
| Limited difficulties | -0.179 | 0.000 | -0.180 | 0.000 | -0.180 | 0.000 | -0.169 | 0.000 | -0.169 | 0.000 |
| Some difficulties | -0.203 | 0.000 | -0.201 | 0.000 | -0.196 | 0.000 | -0.180 | 0.000 | -0.178 | 0.000 |
| Significant difficulties | -0.221 | 0.000 | -0.219 | 0.000 | -0.206 | 0.000 | -0.194 | 0.000 | -0.188 | 0.000 |
| *Bathing in public places (beach, p* | | | | | | | | | | |
| Limited difficulties | -0.145 | 0.000 | -0.144 | 0.000 | -0.145 | 0.000 | -0.145 | 0.000 | -0.144 | 0.000 |
| Some difficulties | -0.138 | 0.000 | -0.138 | 0.000 | -0.140 | 0.000 | -0.143 | 0.000 | -0.142 | 0.000 |
| Significant difficulties | -0.160 | 0.000 | -0.160 | 0.000 | -0.160 | 0.000 | -0.168 | 0.000 | -0.166 | 0.000 |
| *Intimate relations (OP8)* | | | | | | | | | | |
| Limited difficulties | -0.200 | 0.000 | -0.196 | 0.000 | -0.196 | 0.000 | -0.190 | 0.000 | -0.190 | 0.000 |
| Some difficulties | -0.254 | 0.000 | -0.248 | 0.000 | -0.252 | 0.000 | -0.238 | 0.000 | -0.240 | 0.000 |
| Significant difficulties | -0.264 | 0.000 | -0.255 | 0.000 | -0.264 | 0.000 | -0.248 | 0.000 | -0.251 | 0.000 |
| **Patient characteristics** | | | | | | | | | | |
| *Age* [a] | | | | | | | | | | |
| 36-45 yrs | - | - | -0.010 | 0.514 | -0.012 | 0.428 | 0.016 | 0.287 | 0.015 | 0.309 |
| 46-55 yrs | - | - | -0.036 | 0.016 | -0.039 | 0.009 | 0.012 | 0.429 | 0.012 | 0.450 |
| 56-65 yrs | - | - | -0.101 | 0.000 | -0.105 | 0.000 | -0.035 | 0.078 | -0.036 | 0.069 |
| 65+ | - | - | -0.061 | 0.274 | -0.058 | 0.297 | 0.021 | 0.703 | 0.020 | 0.720 |
| Man [b] | - | - | 0.090 | 0.000 | 0.093 | 0.000 | 0.081 | 0.000 | 0.083 | 0.000 |
| *BMI* [d] | | | | | | | | | | |
| 40-44 | - | - | - | - | -0.130 | 0.002 | - | - | -0.082 | 0.049 |
| 45-49 | - | - | - | - | -0.231 | 0.015 | - | - | -0.166 | 0.075 |
| 50+ | - | - | - | - | -0.021 | 0.885 | - | - | -0.010 | 0.947 |
| *Comobidity e* | | | | | | | | | | |
| Sleep apnea | - | - | - | - | - | - | -0.168 | 0.000 | -0.163 | 0.000 |
| Hypertension | - | - | - | - | - | - | -0.096 | 0.000 | -0.095 | 0.000 |
| Diabetes | - | - | - | - | - | - | -0.086 | 0.002 | -0.085 | 0.002 |
| Dyslipidemia | - | - | - | - | - | - | 0.023 | 0.377 | 0.023 | 0.387 |
| Depression | - | - | - | - | - | - | -0.419 | 0.000 | -0.418 | 0.000 |
| **Goodness of fit** | | | | | | | | | | |
| Adj-Rsq, or pseudo R2 | 0.158 | | 0.160 | | 0.161 | | 0.179 | | 0.179 | |
| AIC | -35230 | | -35295 | | -35068 | | -35845 | | -35826 | |
| BIC | -35023 | | -35047 | | -34797 | | -35558 | | -35514 | |
| **Validation** | | | | | | | | | | |
| *MAE* | | | | | | | | | | |
| Training data (80%) | 0.092 | | 0.091 | | 0.091 | | 0.089 | | 0.089 | |
| Validating data (20%) | 0.092 | | 0.092 | | 0.091 | | 0.090 | | 0.090 | |
| Baseline | 0.088 | | 0.088 | | 0.092 | | 0.090 | | 0.092 | |
| 1-year | 0.092 | | 0.091 | | 0.091 | | 0.089 | | 0.089 | |
| 2-year | 0.095 | | 0.095 | | 0.095 | | 0.092 | | 0.092 | |
| 5-year | 0.099 | | 0.098 | | 0.098 | | 0.096 | | 0.096 | |
| RMSE | | | | | | | | | | |
| Training data (80%) | 0.118 | | 0.118 | | 0.118 | | 0.116 | | 0.116 | |
| Validating data (20%) | 0.118 | | 0.117 | | 0.117 | | 0.115 | | 0.115 | |
| Baseline | 0.113 | | 0.113 | | 0.117 | | 0.114 | | 0.116 | |
| 1-year | 0.118 | | 0.118 | | 0.118 | | 0.116 | | 0.116 | |
| 2-year | 0.123 | | 0.123 | | 0.122 | | 0.120 | | 0.120 | |
| 5-year | 0.129 | | 0.128 | | 0.128 | | 0.126 | | 0.125 | |
| RAE | | | | | | | | | | |
| Training data (80%) | 0.815 | | 0.813 | | 0.812 | | 0.795 | | 0.795 | |
| Validating data (20%) | 0.811 | | 0.808 | | 0.808 | | 0.792 | | 0.793 | |
| Baseline | 0.833 | | 0.834 | | 0.860 | | 0.852 | | 0.866 | |
| 1-year | 0.814 | | 0.812 | | 0.811 | | 0.795 | | 0.795 | |
| 2-year | 0.791 | | 0.788 | | 0.789 | | 0.768 | | 0.769 | |
| 5-year | 0.769 | | 0.763 | | 0.765 | | 0.747 | | 0.747 | |
| RRSE | | | | | | | | | | |
| Training data (80%) | 0.858 | | 0.856 | | 0.855 | | 0.840 | | 0.840 | |
| Validating data (20%) | 0.849 | | 0.848 | | 0.849 | | 0.833 | | 0.834 | |
| Baseline | 0.867 | | 0.869 | | 0.885 | | 0.884 | | 0.891 | |
| 1-year | 0.856 | | 0.854 | | 0.854 | | 0.839 | | 0.839 | |
| 2-year | 0.843 | | 0.841 | | 0.841 | | 0.822 | | 0.823 | |
| 5-year | 0.833 | | 0.830 | | 0.831 | | 0.813 | | 0.813 | |

**Mapping algorithm based on 2-year follow-up data**

| | Model 1 coefficient | Model 1 p-Value | Model 2 coefficient | Model 2 p-Value | Model 3 coefficient | Model 3 p-Value | Model 4 coefficient | Model 4 p-Value | Model 5 coefficient | Model 5 p-Value |
|---|---|---|---|---|---|---|---|---|---|---|
| **Intercept** | 1.833 | 0.000 | 1.837 | 0.000 | 1.839 | 0.000 | 1.878 | 0.000 | 1.878 | 0.000 |
| **OP dimensions** [a] | | | | | | | | | | |
| *Private gatherings in my own hor* | | | | | | | | | | |
| Limited difficulties | -0.128 | 0.000 | -0.132 | 0.000 | -0.139 | 0.000 | -0.127 | 0.000 | -0.128 | 0.000 |
| Some difficulties | -0.208 | 0.000 | -0.210 | 0.000 | -0.215 | 0.000 | -0.198 | 0.000 | -0.200 | 0.000 |
| Significant difficulties | -0.338 | 0.000 | -0.340 | 0.000 | -0.344 | 0.000 | -0.321 | 0.000 | -0.326 | 0.000 |
| *Private gatherings in a friend's or* | | | | | | | | | | |
| Limited difficulties | -0.188 | 0.000 | -0.188 | 0.000 | -0.189 | 0.000 | -0.184 | 0.000 | -0.185 | 0.000 |
| Some difficulties | -0.195 | 0.000 | -0.198 | 0.000 | -0.201 | 0.000 | -0.164 | 0.000 | -0.166 | 0.000 |
| Significant difficulties | -0.211 | 0.000 | -0.218 | 0.000 | -0.216 | 0.000 | -0.153 | 0.009 | -0.151 | 0.010 |
| *Going to a restaurant (OP3)* | | | | | | | | | | |
| Limited difficulties | 0.023 | 0.377 | 0.022 | 0.399 | 0.019 | 0.456 | 0.012 | 0.639 | 0.013 | 0.621 |
| Some difficulties | 0.022 | 0.449 | 0.024 | 0.426 | 0.024 | 0.434 | -0.003 | 0.923 | 0.001 | 0.982 |
| Significant difficulties | -0.023 | 0.635 | -0.020 | 0.675 | -0.025 | 0.611 | -0.068 | 0.158 | -0.067 | 0.164 |
| *Going to community activities, co* | | | | | | | | | | |
| Limited difficulties | -0.226 | 0.000 | -0.227 | 0.000 | -0.225 | 0.000 | -0.208 | 0.000 | -0.208 | 0.000 |
| Some difficulties | -0.407 | 0.000 | -0.404 | 0.000 | -0.399 | 0.000 | -0.370 | 0.000 | -0.366 | 0.000 |
| Significant difficulties | -0.571 | 0.000 | -0.571 | 0.000 | -0.561 | 0.000 | -0.496 | 0.000 | -0.494 | 0.000 |
| *Vacations away from home (OP5,* | | | | | | | | | | |
| Limited difficulties | -0.038 | 0.159 | -0.041 | 0.124 | -0.037 | 0.176 | -0.032 | 0.228 | -0.030 | 0.262 |
| Some difficulties | -0.064 | 0.044 | -0.068 | 0.033 | -0.065 | 0.046 | -0.070 | 0.027 | -0.071 | 0.026 |
| Significant difficulties | -0.005 | 0.918 | -0.011 | 0.808 | -0.012 | 0.803 | -0.029 | 0.535 | -0.030 | 0.513 |
| *Trying on and buying clothes (OP(* | | | | | | | | | | |
| Limited difficulties | -0.122 | 0.000 | -0.123 | 0.000 | -0.121 | 0.000 | -0.115 | 0.000 | -0.112 | 0.000 |
| Some difficulties | -0.152 | 0.000 | -0.150 | 0.000 | -0.143 | 0.000 | -0.148 | 0.000 | -0.143 | 0.000 |
| Significant difficulties | -0.160 | 0.000 | -0.157 | 0.000 | -0.142 | 0.000 | -0.144 | 0.000 | -0.132 | 0.000 |
| *Bathing in public places (beach, p* | | | | | | | | | | |
| Limited difficulties | -0.174 | 0.000 | -0.172 | 0.000 | -0.173 | 0.000 | -0.179 | 0.000 | -0.178 | 0.000 |
| Some difficulties | -0.159 | 0.000 | -0.160 | 0.000 | -0.162 | 0.000 | -0.168 | 0.000 | -0.166 | 0.000 |
| Significant difficulties | -0.150 | 0.000 | -0.152 | 0.000 | -0.152 | 0.000 | -0.167 | 0.000 | -0.164 | 0.000 |
| *Intimate relations (OP8)* | | | | | | | | | | |
| Limited difficulties | -0.184 | 0.000 | -0.181 | 0.000 | -0.178 | 0.000 | -0.160 | 0.000 | -0.162 | 0.000 |
| Some difficulties | -0.252 | 0.000 | -0.244 | 0.000 | -0.242 | 0.000 | -0.218 | 0.000 | -0.222 | 0.000 |
| Significant difficulties | -0.282 | 0.000 | -0.270 | 0.000 | -0.268 | 0.000 | -0.245 | 0.000 | -0.251 | 0.000 |
| **Patient characteristics** | | | | | | | | | | |
| *Age* [a] | | | | | | | | | | |
| 36-45 yrs | - | - | -0.005 | 0.781 | -0.003 | 0.862 | 0.019 | 0.313 | 0.020 | 0.306 |
| 46-55 yrs | - | - | -0.041 | 0.030 | -0.040 | 0.036 | 0.005 | 0.809 | 0.005 | 0.814 |
| 56-65 yrs | - | - | -0.075 | 0.001 | -0.073 | 0.001 | -0.020 | 0.408 | -0.020 | 0.410 |
| 65+ | - | - | -0.080 | 0.120 | -0.089 | 0.085 | -0.036 | 0.495 | -0.037 | 0.479 |
| Man [b] | - | - | 0.099 | 0.000 | 0.102 | 0.000 | 0.086 | 0.000 | 0.088 | 0.000 |
| *BMI* [d] | | | | | | | | | | |
| 40-44 | - | - | - | - | -0.162 | 0.002 | - | - | -0.118 | 0.020 |
| 45-49 | - | - | - | - | -0.167 | 0.102 | - | - | -0.106 | 0.298 |
| 50+ | - | - | - | - | -0.573 | 0.004 | - | - | -0.588 | 0.002 |
| *Comobidity e* | | | | | | | | | | |
| Sleep apnea | - | - | - | - | - | - | -0.169 | 0.000 | -0.157 | 0.000 |
| Hypertension | - | - | - | - | - | - | -0.057 | 0.004 | -0.056 | 0.005 |
| Diabetes | - | - | - | - | - | - | -0.047 | 0.139 | -0.052 | 0.102 |
| Dyslipidemia | - | - | - | - | - | - | -0.007 | 0.821 | -0.005 | 0.873 |
| Depression | - | - | - | - | - | - | -0.422 | 0.000 | -0.421 | 0.000 |
| **Goodness of fit** | | | | | | | | | | |
| Adj-Rsq, or pseudo R2 | 0.177 | | 0.179 | | 0.178 | | 0.197 | | 0.197 | |
| AIC | -21149 | | -21189 | | -20773 | | -21343 | | -21285 | |
| BIC | -20953 | | -20955 | | -20517 | | -21073 | | -20991 | |
| **Validation** | | | | | | | | | | |
| *MAE* | | | | | | | | | | |
| Training data (80%) | 0.096 | | 0.095 | | 0.095 | | 0.093 | | 0.093 | |
| Validating data (20%) | 0.096 | | 0.096 | | 0.095 | | 0.092 | | 0.093 | |
| Baseline | 0.088 | | 0.088 | | 0.096 | | 0.090 | | 0.095 | |
| 1-year | 0.093 | | 0.092 | | 0.092 | | 0.090 | | 0.091 | |
| 2-year | 0.096 | | 0.095 | | 0.095 | | 0.093 | | 0.093 | |
| 5-year | 0.099 | | 0.098 | | 0.098 | | 0.096 | | 0.096 | |
| RMSE | | | | | | | | | | |
| Training data (80%) | 0.122 | | 0.122 | | 0.122 | | 0.119 | | 0.119 | |
| Validating data (20%) | 0.122 | | 0.122 | | 0.121 | | 0.118 | | 0.118 | |
| Baseline | 0.112 | | 0.112 | | 0.122 | | 0.113 | | 0.121 | |
| 1-year | 0.118 | | 0.118 | | 0.118 | | 0.116 | | 0.116 | |
| 2-year | 0.122 | | 0.122 | | 0.122 | | 0.119 | | 0.119 | |
| 5-year | 0.128 | | 0.127 | | 0.127 | | 0.124 | | 0.124 | |
| RAE | | | | | | | | | | |
| Training data (80%) | 0.791 | | 0.788 | | 0.790 | | 0.770 | | 0.771 | |
| Validating data (20%) | 0.784 | | 0.781 | | 0.777 | | 0.756 | | 0.756 | |
| Baseline | 0.831 | | 0.832 | | 0.886 | | 0.848 | | 0.892 | |
| 1-year | 0.814 | | 0.812 | | 0.812 | | 0.795 | | 0.796 | |
| 2-year | 0.790 | | 0.787 | | 0.787 | | 0.767 | | 0.768 | |
| 5-year | 0.765 | | 0.760 | | 0.763 | | 0.745 | | 0.745 | |
| RRSE | | | | | | | | | | |
| Training data (80%) | 0.843 | | 0.841 | | 0.842 | | 0.823 | | 0.824 | |
| Validating data (20%) | 0.832 | | 0.830 | | 0.826 | | 0.806 | | 0.806 | |
| Baseline | 0.863 | | 0.865 | | 0.902 | | 0.878 | | 0.913 | |
| 1-year | 0.857 | | 0.855 | | 0.856 | | 0.840 | | 0.841 | |
| 2-year | 0.841 | | 0.839 | | 0.839 | | 0.820 | | 0.820 | |
| 5-year | 0.830 | | 0.827 | | 0.829 | | 0.810 | | 0.810 | |

**Mapping algorithm based on 5-year follow-up data**

| | Model 1 coefficient | Model 1 p-Value | Model 2 coefficient | Model 2 p-Value | Model 3 coefficient | Model 3 p-Value | Model 4 coefficient | Model 4 p-Value | Model 5 coefficient | Model 5 p-Value |
|---|---|---|---|---|---|---|---|---|---|---|
| **Intercept** | 1.701 | 0.000 | 1.750 | 0.000 | 1.752 | 0.000 | 1.804 | 0.000 | 1.800 | 0.000 |
| **OP dimensions** [a] | | | | | | | | | | |
| *Private gatherings in my own hor* | | | | | | | | | | |
| Limited difficulties | -0.095 | 0.015 | -0.096 | 0.014 | -0.105 | 0.009 | -0.076 | 0.055 | -0.086 | 0.028 |
| Some difficulties | -0.170 | 0.001 | -0.165 | 0.001 | -0.185 | 0.000 | -0.151 | 0.003 | -0.162 | 0.002 |
| Significant difficulties | -0.362 | 0.000 | -0.356 | 0.000 | -0.382 | 0.000 | -0.327 | 0.000 | -0.340 | 0.000 |
| *Private gatherings in a friend's or* | | | | | | | | | | |
| Limited difficulties | -0.098 | 0.020 | -0.106 | 0.012 | -0.099 | 0.021 | -0.101 | 0.017 | -0.095 | 0.025 |
| Some difficulties | -0.109 | 0.043 | -0.116 | 0.031 | -0.108 | 0.050 | -0.085 | 0.116 | -0.079 | 0.143 |
| Significant difficulties | -0.166 | 0.034 | -0.166 | 0.035 | -0.153 | 0.058 | -0.108 | 0.173 | -0.092 | 0.248 |
| *Going to a restaurant (OP3)* | | | | | | | | | | |
| Limited difficulties | 0.024 | 0.522 | 0.021 | 0.570 | 0.024 | 0.542 | 0.014 | 0.713 | 0.018 | 0.628 |
| Some difficulties | 0.007 | 0.881 | 0.001 | 0.974 | 0.005 | 0.919 | -0.026 | 0.550 | -0.017 | 0.700 |
| Significant difficulties | -0.015 | 0.830 | -0.025 | 0.723 | -0.039 | 0.593 | -0.085 | 0.237 | -0.092 | 0.203 |
| *Going to community activities, co* | | | | | | | | | | |
| Limited difficulties | -0.173 | 0.000 | -0.173 | 0.000 | -0.173 | 0.000 | -0.165 | 0.000 | -0.162 | 0.000 |
| Some difficulties | -0.326 | 0.000 | -0.324 | 0.000 | -0.318 | 0.000 | -0.283 | 0.000 | -0.282 | 0.000 |
| Significant difficulties | -0.496 | 0.000 | -0.495 | 0.000 | -0.466 | 0.000 | -0.389 | 0.000 | -0.383 | 0.000 |
| *Vacations away from home (OP5,* | | | | | | | | | | |
| Limited difficulties | -0.052 | 0.170 | -0.045 | 0.239 | -0.038 | 0.325 | -0.034 | 0.370 | -0.039 | 0.308 |
| Some difficulties | -0.028 | 0.538 | -0.025 | 0.576 | -0.022 | 0.630 | -0.030 | 0.501 | -0.038 | 0.396 |
| Significant difficulties | -0.036 | 0.574 | -0.040 | 0.532 | -0.010 | 0.883 | -0.011 | 0.868 | -0.007 | 0.919 |
| *Trying on and buying clothes (OP(* | | | | | | | | | | |
| Limited difficulties | -0.191 | 0.000 | -0.195 | 0.000 | -0.202 | 0.000 | -0.189 | 0.000 | -0.186 | 0.000 |
| Some difficulties | -0.243 | 0.000 | -0.245 | 0.000 | -0.250 | 0.000 | -0.239 | 0.000 | -0.237 | 0.000 |
| Significant difficulties | -0.276 | 0.000 | -0.275 | 0.000 | -0.292 | 0.000 | -0.279 | 0.000 | -0.284 | 0.000 |
| *Bathing in public places (beach, p* | | | | | | | | | | |
| Limited difficulties | -0.094 | 0.008 | -0.090 | 0.011 | -0.078 | 0.031 | -0.081 | 0.022 | -0.077 | 0.030 |
| Some difficulties | -0.099 | 0.005 | -0.101 | 0.004 | -0.100 | 0.005 | -0.103 | 0.003 | -0.099 | 0.005 |
| Significant difficulties | -0.115 | 0.005 | -0.120 | 0.004 | -0.114 | 0.007 | -0.111 | 0.007 | -0.104 | 0.012 |
| *Intimate relations (OP8)* | | | | | | | | | | |
| Limited difficulties | -0.237 | 0.000 | -0.235 | 0.000 | -0.225 | 0.000 | -0.227 | 0.000 | -0.224 | 0.000 |
| Some difficulties | -0.284 | 0.000 | -0.278 | 0.000 | -0.277 | 0.000 | -0.267 | 0.000 | -0.267 | 0.000 |
| Significant difficulties | -0.336 | 0.000 | -0.326 | 0.000 | -0.329 | 0.000 | -0.318 | 0.000 | -0.319 | 0.000 |
| **Patient characteristics** | | | | | | | | | | |
| *Age* [a] | | | | | | | | | | |
| 36-45 yrs | - | - | -0.076 | 0.016 | -0.085 | 0.009 | -0.072 | 0.023 | -0.072 | 0.024 |
| 46-55 yrs | - | - | -0.079 | 0.009 | -0.078 | 0.012 | -0.050 | 0.105 | -0.048 | 0.124 |
| 56-65 yrs | - | - | -0.114 | 0.001 | -0.117 | 0.001 | -0.072 | 0.039 | -0.075 | 0.030 |
| 65+ | - | - | -0.082 | 0.112 | -0.088 | 0.097 | -0.051 | 0.346 | -0.056 | 0.299 |
| Man [b] | - | - | 0.114 | 0.000 | 0.120 | 0.000 | 0.097 | 0.000 | 0.101 | 0.000 |
| *BMI* [d] | | | | | | | | | | |
| 40-44 | - | - | - | - | -0.077 | 0.174 | - | - | -0.053 | 0.346 |
| 45-49 | - | - | - | - | -0.145 | 0.189 | - | - | -0.088 | 0.422 |
| 50+ | - | - | - | - | 0.060 | 0.794 | - | - | 0.151 | 0.510 |
| *Comobidity e* | | | | | | | | | | |
| Sleep apnea | - | - | - | - | - | - | -0.029 | 0.636 | -0.027 | 0.668 |
| Hypertension | - | - | - | - | - | - | -0.050 | 0.062 | -0.048 | 0.075 |
| Diabetes | - | - | - | - | - | - | -0.076 | 0.078 | -0.069 | 0.115 |
| Dyslipidemia | - | - | - | - | - | - | -0.001 | 0.977 | 0.003 | 0.940 |
| Depression | - | - | - | - | - | - | -0.450 | 0.000 | -0.450 | 0.000 |
| **Goodness of fit** | | | | | | | | | | |
| Adj-Rsq, or pseudo R2 | 0.208 | | 0.211 | | 0.208 | | 0.233 | | 0.233 | |
| AIC | -8210 | | -8233 | | -7935 | | -8268 | | -8217 | |
| BIC | -8036 | | -8025 | | -7708 | | -8028 | | -7958 | |
| **Validation** | | | | | | | | | | |
| *MAE* | | | | | | | | | | |
| Training data (80%) | 0.099 | | 0.098 | | 0.098 | | 0.096 | | 0.095 | |
| Validating data (20%) | 0.099 | | 0.098 | | 0.098 | | 0.096 | | 0.096 | |
| Baseline | 0.093 | | 0.092 | | 0.095 | | 0.092 | | 0.093 | |
| 1-year | 0.096 | | 0.096 | | 0.096 | | 0.094 | | 0.094 | |
| 2-year | 0.098 | | 0.098 | | 0.098 | | 0.096 | | 0.095 | |
| 5-year | 0.099 | | 0.098 | | 0.098 | | 0.096 | | 0.096 | |
| RMSE | | | | | | | | | | |
| Training data (80%) | 0.126 | | 0.125 | | 0.125 | | 0.122 | | 0.121 | |
| Validating data (20%) | 0.125 | | 0.124 | | 0.124 | | 0.122 | | 0.122 | |
| Baseline | 0.118 | | 0.117 | | 0.121 | | 0.116 | | 0.118 | |
| 1-year | 0.120 | | 0.119 | | 0.119 | | 0.117 | | 0.117 | |
| 2-year | 0.123 | | 0.122 | | 0.122 | | 0.119 | | 0.119 | |
| 5-year | 0.125 | | 0.125 | | 0.125 | | 0.122 | | 0.121 | |
| RAE | | | | | | | | | | |
| Training data (80%) | 0.762 | | 0.759 | | 0.760 | | 0.739 | | 0.739 | |
| Validating data (20%) | 0.755 | | 0.753 | | 0.754 | | 0.741 | | 0.739 | |
| Baseline | 0.856 | | 0.854 | | 0.873 | | 0.865 | | 0.875 | |
| 1-year | 0.818 | | 0.817 | | 0.817 | | 0.800 | | 0.800 | |
| 2-year | 0.794 | | 0.792 | | 0.793 | | 0.773 | | 0.774 | |
| 5-year | 0.761 | | 0.758 | | 0.759 | | 0.740 | | 0.739 | |
| RRSE | | | | | | | | | | |
| Training data (80%) | 0.823 | | 0.820 | | 0.821 | | 0.799 | | 0.798 | |
| Validating data (20%) | 0.825 | | 0.822 | | 0.823 | | 0.810 | | 0.808 | |
| Baseline | 0.870 | | 0.872 | | 0.883 | | 0.884 | | 0.892 | |
| 1-year | 0.860 | | 0.858 | | 0.857 | | 0.843 | | 0.842 | |
| 2-year | 0.842 | | 0.840 | | 0.840 | | 0.821 | | 0.822 | |
| 5-year | 0.823 | | 0.820 | | 0.821 | | 0.801 | | 0.800 | |

**Reference group**
**a no difficulties**
**b 18-34 years**
**c Women**
**d <=39**
**e No disease**

Figure S1. Distribution of SF-6D index, for baseline, 1,2, 5-year, respectively.

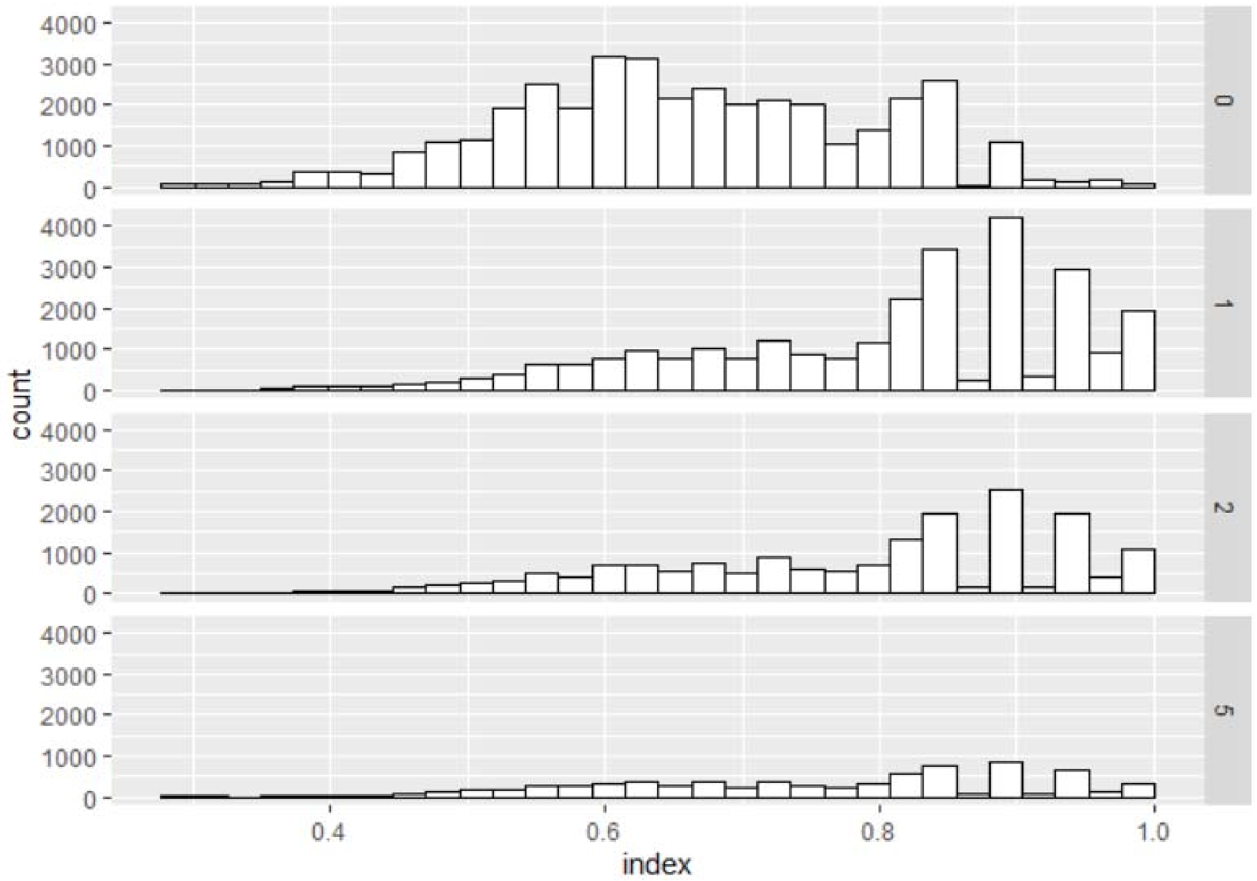

Figure S2. Boxplot of SF-6D index, for baseline, 1,2, 5-year, respectively.

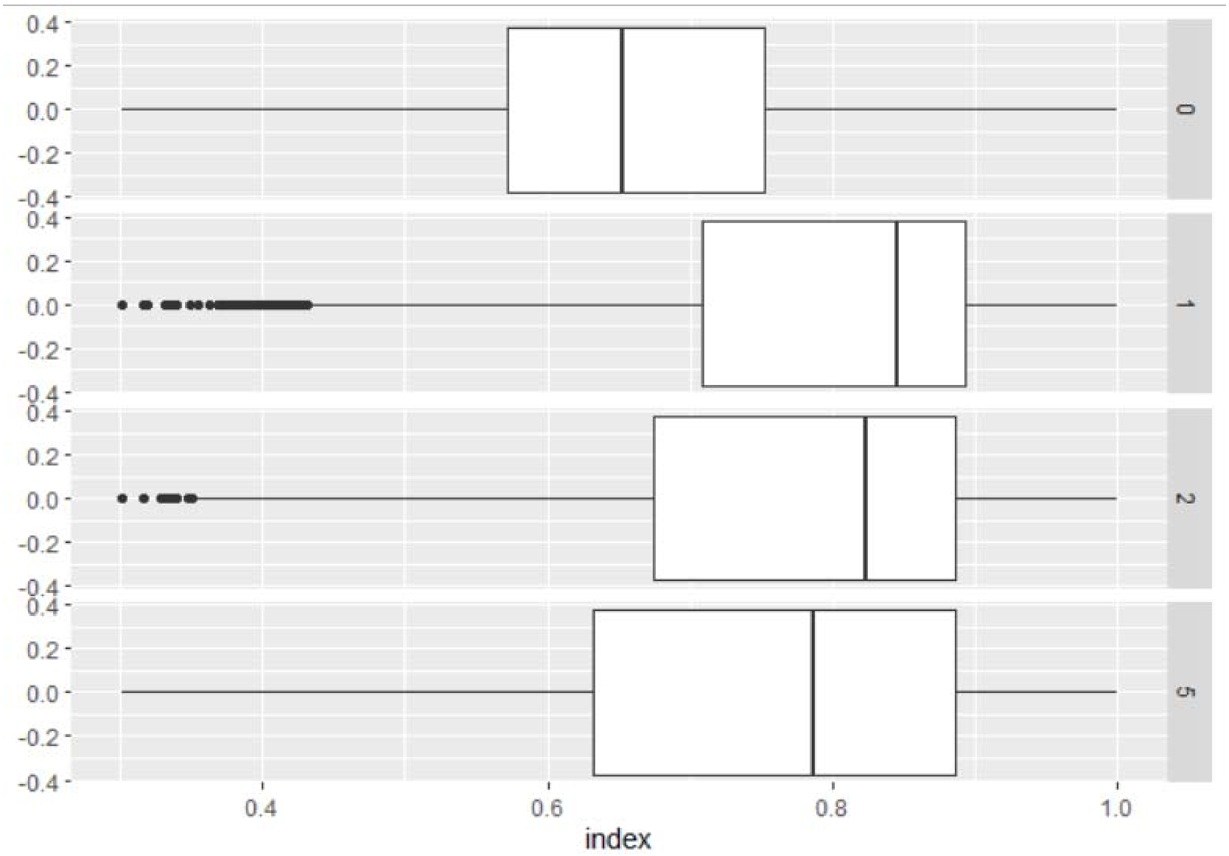

TRIPOD Checklist: Prediction Model Development and Validation

| Section/Topic | Item | | Checklist Item | Page |
|---|---|---|---|---|
| **Title and abstract** | | | | |
| Title | 1 | D;V | Identify the study as developing and/or validating a multivariable prediction model, the target population, and the outcome to be predicted. | P1 |
| Abstract | 2 | D;V | Provide a summary of objectives, study design, setting, participants, sample size, predictors, outcome, statistical analysis, results, and conclusions. | P2 |
| **Introduction** | | | | |
| Background and objectives | 3a | D;V | Explain the medical context (including whether diagnostic or prognostic) and rationale for developing or validating the multivariable prediction model, including references to existing models. | P3 para1 |
| | 3b | D;V | Specify the objectives, including whether the study describes the development or validation of the model or both. | |
| **Methods** | | | | |
| Source of data | 4a | D;V | Describe the study design or source of data (e.g., randomized trial, cohort, or registry data), separately for the development and validation data sets, if applicable. | P4 para2 |
| | 4b | D;V | Specify the key study dates, including start of accrual; end of accrual; and, if applicable, end of follow-up. | P5 para 1 |
| Participants | 5a | D;V | Specify key elements of the study setting (e.g., primary care, secondary care, general population) including number and location of centres. | P5 para 1 |
| | 5b | D;V | Describe eligibility criteria for participants. | P5 para 1 |
| | 5c | D;V | Give details of treatments received, if relevant. | Not relevant |
| Outcome | 6a | D;V | Clearly define the outcome that is predicted by the prediction model, including how and when assessed. | P5, from line 26<br>P6, line from 20 |
| | 6b | D;V | Report any actions to blind assessment of the outcome to be predicted. | Not relevant |
| Predictors | 7a | D;V | Clearly define all predictors used in developing or validating the multivariable prediction model, including how and when they were measured. | P6 line 7 to p7 line 10 |
| | 7b | D;V | Report any actions to blind assessment of predictors for the outcome and other predictors. | Not relevant |
| Sample size | 8 | D;V | Explain how the study size was arrived at. | Not relevant |
| Missing data | 9 | D;V | Describe how missing data were handled (e.g., complete-case analysis, single imputation, multiple imputation) with details of any imputation method. | P5, line 18-20 |
| Statistical analysis methods | 10a | D | Describe how predictors were handled in the analyses. | P6 line 7 to p7 line 10 |
| | 10b | D | Specify type of model, all model-building procedures (including any predictor selection), and method for internal validation. | P6 line 7 to p7 line 10 |
| | 10c | V | For validation, describe how the predictions were calculated. | P5 line 12-22 |
| | 10d | D;V | Specify all measures used to assess model performance and, if relevant, to compare multiple models. | P7, line14-17 |
| | 10e | V | Describe any model updating (e.g., recalibration) arising from the validation, if done. | Not relevant |
| Risk groups | 11 | D;V | Provide details on how risk groups were created, if done. | Not relevant |
| Development vs. validation | 12 | V | For validation, identify any differences from the development data in setting, eligibility criteria, outcome, and predictors. | |
| **Results** | | | | |
| Participants | 13a | D;V | Describe the flow of participants through the study, including the number of participants with and without the outcome and, if applicable, a summary of the follow-up time. A diagram may be helpful. | Page 5 para 1 |
| | 13b | D;V | Describe the characteristics of the participants (basic demographics, clinical features, available predictors), including the number of participants with missing data for predictors and outcome. | Page 8 para 1, table 1, table s3 and s4 |
| | 13c | V | For validation, show a comparison with the development data of the distribution of important variables (demographics, predictors and outcome). | table 1, table s3 |
| Model development | 14a | D | Specify the number of participants and outcome events in each analysis. | Table S3 |
| | 14b | D | If done, report the unadjusted association between each candidate predictor and outcome. | Model 1 in Table S5A-S8B |
| Model specification | 15a | D | Present the full prediction model to allow predictions for individuals (i.e., all regression coefficients, and model intercept or baseline survival at a given time point). | Table S5A-S8B |
| | 15b | D | Explain how to the use the prediction model. | Page 9, from line 28 |
| Model performance | 16 | D;V | Report performance measures for the prediction model. | Table 3, Table S5A-S8B, page 8-10 |
| Model-updating | 17 | V | If done, report the results from any model updating (i.e., model specification, model performance). | Not relevant |
| **Discussion** | | | | |
| Limitations | 18 | D;V | Discuss any limitations of the study (such as non-representative sample, few events per predictor, missing data). | Page 9 line 11-15 |
| Interpretation | 19a | V | For validation, discuss the results with reference to performance in the development data, and any other validation data. | Page 13 line 3-7 |
| | 19b | D;V | Give an overall interpretation of the results, considering objectives, limitations, results from similar studies, and other relevant evidence. | Page 11 para 1, page 13 line 9-14 |
| Implications | 20 | D;V | Discuss the potential clinical use of the model and implications for future research. | Page 11para 2, page 12 line 21-25 |
| **Other information** | | | | |
| Supplementary | 21 | D;V | Provide information about the availability of supplementary resources, | Supplementary |

| | | | | |
|---|---|---|---|---|
| information | | | such as study protocol, Web calculator, and data sets. | material |
| Funding | 22 | D;V | Give the source of funding and the role of the funders for the present study. | Title page |

*Items relevant only to the development of a prediction model are denoted by D, items relating solely to a validation of a prediction model are denoted by V, and items relating to both are denoted D;V. We recommend using the TRIPOD Checklist in conjunction with the TRIPOD Explanation and Elaboration document.

Table S9. Test of model with and without link function, results based on model 1

| | logit | probit | cloglog | cauchit | loglog |
|---|---|---|---|---|---|
| AIC | | | | | |
| ops without link func | -46124 | -46091 | -45980 | -46249 | -46186 |
| ops link | -46525 | -46485 | -46359 | -46702 | -46600 |
| BIC | | | | | |
| ops | -46100 | -46066 | -45955 | -46224 | -46161 |
| ops link | -46492 | -46452 | -46326 | -46669 | -46567 |
